# Miniature inverted repeat transposable elements in the genome of sugar beet and their impact on gene expression

**DOI:** 10.1038/s41598-025-32772-7

**Published:** 2025-12-19

**Authors:** Emilia Morańska, Gabriela Machaj, Wiktoria Piestrzyńska, Monika Szewczyk, Marcelina Skrabucha, Adam Sitarski, Alicja Macko-Podgórni, Dariusz Grzebelus

**Affiliations:** 1 Department of Plant Biology and Biotechnology, Faculty of Biotechnology and Horticulture, University of Agriculture in Kraków, Kraków, Poland; 2 Kutnowska Hodowla Buraka Cukrowego W Straszkowie Sp. Z O.O., Straszków, Poland

**Keywords:** *Beta vulgaris*, Differentially expressed genes, Insertional polymorphism, MITEs, DNA transposons, Computational biology and bioinformatics, Genetics, Molecular biology, Plant sciences

## Abstract

Miniature inverted-repeat transposable elements (MITEs) are the most abundant group among class II mobile elements in plant genomes. They are frequently located in gene-rich regions, which may affect gene structure and expression, leading to functional diversity. Our research aimed to perform a comprehensive global annotation of MITEs in the sugar beet genome, which has been lacking to date. We also attempted to elucidate the association between the presence of MITE insertions and the regulation of gene expression. Analysis of the MITEs distribution in sugar beet revealed that MITEs cover about 3% of the genome, with the largest group comprising *Stowaway*-like elements. Approximately 60% of all identified MITEs were located within genic regions, indicating their potential impact on gene expression regulation. *Stowaway* and *Tourist*-like elements were frequently present in introns and downstream from genes. *Tourists* were also relatively more enriched in 3’UTRs than the other MITE groups. *hAT*-like MITEs were present mainly in introns and 5’UTRs, while *Mutator*-like elements were relatively more frequently located in 5’UTRs than in the other MITE groups. Our study also showed that a considerable portion of the differentially expressed genes in plants from two F2 sugar beet families was associated with MITEs, especially *Stowaways* and *Tourists*. We provided a comprehensive landscape of MITE distribution in the sugar beet genome and described examples of MITE insertions located in the regulatory regions of genes that show significant differential expression. These results will facilitate research into the role of MITEs in their potential impact on gene expression regulation.

**Supplementary Information:**

The online version contains supplementary material available at 10.1038/s41598-025-32772-7.

## Introduction

Miniature inverted-repeat transposable elements (MITEs) belong to the non-autonomous class II mobile elements and are widely distributed and abundant in plant genomes. They are characterised by their small size (< 800 bp), high copy number and usually AT-rich sequences with no coding capacity^1^. They can be mobilised by trans-acting related autonomous class II elements. MITEs comprise several groups depending on the similarity of their terminal inverted repeats (TIRs) and target site duplications (TSDs) to those of elements representing superfamilies of class II transposable elements (DNA transposons). The first described and most widely characterised groups were *Stowaways* and *Tourists*, attributed to *Tc1/Mariner* and *PIF/Harbinger* superfamilies, respectively^2,3^. Furthermore, MITEs related to other superfamilies of DNA transposons, i.e. *Mutators* and *hATs*, were also reported^4,5^.

MITEs are often located in gene-rich regions, which may affect the function of proximal genes. Disruption of gene structure by MITE activity has been reported^6–8^, and the importance of MITE insertions for the regulation of gene expression has also been described^9–12^. The presence of a MITE copy in the promoter region may result in the epigenetic downregulation of the gene^13,14^. It has also been shown that an insertion upstream of a gene may cause its upregulation^15,16^. MITEs may contain transcription factor binding sites (TFBS)^17–19^ or cis-regulatory elements (CREs), they can also provide novel splicing sites^12,20^, transcription start sites, and polyadenylation signals^21^. Location of a MITE copy in the 3’UTR region, in turn, may significantly affect translation or RNA stability as part of the post-transcriptional regulation^21,22^. MITEs can also induce siRNA formation and gene silencing through RNA-directed methylation (RdDM)^23,24^. However, to study the effects of MITEs on gene function, their prior identification and characterisation throughout the host genome is essential.

Sugar beet (*Beta vulgaris* subsp. *vulgaris* L.) is an important temperate climate crop, accounting for nearly 30% of the world’s annual sugar production, and a source of bioethanol and animal feed. The taxonomic classification assigns the genus *Beta* to the Amaranthaceae family within Caryophylalles. Sugar beet is a diploid plant (2n = 18) with an estimated genome size of 714–758 Mb, of which the repetitive fraction constitutes around 64%^25,26^. More than 180,000 repetitive elements were identified in the EL10.1 sugar beet reference genome (NCBI accession: GCF_002917755.1), of which DNA transposons were the most abundant group (58.1%), followed by LTR elements (36.0%)^26^. To date, two groups of sugar beet MITEs have been more thoroughly characterised, i.e., VulMITEs attributed to the *Stowaway* group^27,28^ and *BvhATs* belonging to the *hAT* group^29^. Nevertheless, a comprehensive, global annotation of sugar beet MITEs has been lacking.

Here, we report on the global characterisation of sugar beet MITEs, their genomic distribution, association with genes and presence in transcripts. We also provided examples of MITE insertions located in regulatory regions of genes showing significant differential expression. Our findings will facilitate research on the role of MITEs in the host genome and extend the knowledge of the potential effect of mobile genetic elements on the regulation of gene expression.

## Materials and methods

### Plant material and nucleic acids extraction

Two sugar beet F2 families, P1 and P4, were obtained from two independent crosses involving four parental lines used in the sugar beet breeding programme of Kutnowska Hodowla Buraka Cukrowego (KHBC), Poland. Fresh roots and mature leaves from 24 plants representing the two segregating populations (12 plants each) were used for DNA and RNA isolation (Table S1 in Supplementary Materials 2).

Genomic DNA was extracted from leaves and roots using a modified CTAB method^30^. DNA samples were purified and treated with RNase using a NucleoSpin gDNA Clean-up Kit (Macherey–Nagel). Total RNA was extracted from fresh roots using PureLink® RNA Mini Kit (Thermo Fisher Scientific). mRNA was obtained from RNA samples using NEBNext® Poly(A) mRNA Magnetic Isolation Module (NEB, Herts, UK), and quality was controlled with Bioanalyzer 2100 (Agilent Technologies, Palo Alto, CA, USA).

### WGS data analysis

Library preparation and pair-end sequencing (PE150 mode) using HighSeq 4000 (Illumina; San Diego, CA, USA) next-generation sequencing platform were performed by Genomed S.A. (Poland). The quality of raw DNA reads was controlled using Trimmomatic^31^ v0.39. Adaptor sequences and low-quality reads were removed with the following parameters: LEADING:20, TRAILING:20, SLIDINGWINDOW:5:20, MINLEN:50, TrueSeq3—PE; fa.2:30:10. The reference sugar beet genome used for analysis was EL10_1.0 (NCBI accession: GCF_002917755.1). Genome indexing and DNAseq reads mapping were performed using BWA^32^ v0.7.17 with the default parameters. Duplicates were checked using the Samblaster v0.1.25 tool^33^ (Table S1 in Supplementary Materials 2).

Mapped DNAseq reads were sorted using SAMtools^34^ v1.11 and merged into one file using Picard^35^ v2.23.0. Subsequently, Freebayes^36^ v1.3.2 was used to call SNPs. The results were filtered using VCFlib^37^ v1.0.1 with the following parameters: QUAL > 20 & QUAL/AO > 10 & SAF > 0 & SAR > 0 & RPR > 1 & RPL > 1 and VCFtools^38^ v0.1.16 with the parameters: –max-missing 0.8, –remove-indels. SNP analysis was performed using BCFtools^39^ v1.11, RTG-toolkit v3.11 and VCFtools^38^ v0.1.16.

Based on the results reporting SNPs for P1 and P4 populations, we found records representing regions of homozygosity for 3 or more samples with ‘1/1’ and ‘0/0’ calls (describing two types of homozygotes, respectively), using a custom Python script^40^. We allowed up to 2 mismatches between records to achieve the longest homozygosity regions possible. The results were then converted into homozygosity bins (intervals), reporting the ‘start’ and ‘end’ positions for each bin (interval) and three samples representing each type of homozygote, respectively.

To identify genes in homozygosity bins, interval localisations were compared with genic regions according to the EL10_1.0 reference sugar beet genome annotation file (NCBI accession: GCF_002917755.1) and the sum of the genes for each bin was calculated using Bedools v.2.26.0^41^. Further, we typed the sample sets for differential expression (DE) analysis, based on homozygosity bins with 20 or more genes, using a custom Python script^40^. Each set comprised three samples of each homozygote type.

### RNAseq data analysis

Library preparation and pair-end sequencing (PE150 mode) using HighSeq 4000 (Illumina; San Diego, CA, USA) next-generation sequencing platform were performed by Genomed S.A. (Poland). The raw RNA reads were pre-processed by removing adaptor sequences and low-quality reads using Trimmomatic^31^ v0.39 with the following parameters: LEADING: 20, TRAILING: 20, SLIDINGWINDOW: 5:20, MINLEN: 50, TrueSeq3-PE; fa.2:30:10. The reference sugar beet genome used for analysis was EL10_1.0 (NCBI accession: GCF_002917755.1). Genome indexing and RNAseq reads mapping were performed using STAR^42^ v2.6.0 with the following parameters: outSAMmapqUnique: 50; outFilterMultimapNmax: 20; alignSJoverhangMin: 8; alignSJDBoverhangMin: 1; outFilterMismatchNmax: 999; outFilterMismatchNoverLmax: 0.04; alignIntronMin: 20; alignIntronMax: 1,000,000; alignMatesGapMax: 1,000,000 (Table S1 in the Supplementary Materials 2).

The protocol described by Machaj et al.^43^ was used to quantify the expression level in sample sets typed from homozygosity regions and to identify differentially expressed genes (DEGs). Kallisto^44^ v0.46.2 was used to estimate the expression of each identified transcript in each sample separately. Read counts for transcripts and genes were expressed in transcripts per million (TPM) and estimated (est) count units. Differential expression of genes for each sample set was calculated using different algorithms with three R packages: DESeq2^45^ v1.0, EBSeq^46^ v1.11.1 and edgeR^47^ v3.14.0. In each calculation, we identified genes (minimum read count: 10) differentially expressed in plants with two types of homozygotes. For general analysis, we considered only genes significantly differentially expressed at a *p-adj* parameter value less than 0.05.

### In silico identification of MITE insertions

Sugar beet MITE families were identified in the RefBeet (NCBI accession: GCF_000511025.2) and EL10_1.0 reference genome (NCBI accession: GCF_002917755.1) using MITE-Hunter^48^, as described by Macko-Podgórni et al.^20^. The final database comprised MITE consensus sequences representing 170 MITE families, grouped into five superfamilies (File S3 from Supplementary Materials). Subsequently, we used PopoolationTE2^49^ and the sugar beet MITE database to identify MITE insertion sites for P1 and P4 populations. The method required a masking reference genome step, which was performed using RepeatMasker^50^ v4.1.3 with the following parameters: -gccalc, -s, -cutoff 200, -no_is, -nolow, -norna, -gff, -u, -pa 4. Subsequently, the reference genome was indexed using BWA^32^ v0.7.17 with the -index flag, and the mapping step was performed with the BWA -mem algorithm and parameters: -M, -t 30. Identification of MITE insertion sites was conducted following a walkthrough described in the manual with default parameters. In brief, the analysis included the following steps: restoring paired-end information in bam files, generating the ppileup file (which summarises for every site in the genome the structural status inferred from paired-end read covering the given site), identifying signatures of TE insertions, estimating population frequencies and pairing up signatures of TE insertions, yielding a final list of TE insertions. Next, the file containing information about insertion sites for each MITE family was filtered to separate reference and non-reference insertions and then converted into a matrix that reports MITEs along with their coordinates for all samples in the population, using custom Python scripts^40^. The absence of a TE insertion was expressed as a frequency = 0, and the presence of an insertion was defined as the mean of paired signatures F and R frequency (for reference insertions) or frequency of FR (or F/R) signature (for non-reference insertions), respectively. In the final matrix, reference and non-reference insertions were combined and considered jointly in subsequent analyses.

The final matrix was used for the calculation of MITE insertions for each family and all samples, with the division for homo- and heterozygous insertions, assuming that the reported frequency by PopoolationTE2, higher than 0.7, defines a homozygous insertion, the frequency between 0.3 and 0.7 indicates a heterozygous insertion and a frequency below 0.3 is a homozygous empty site.

The presence of MITE insertions in the context of genic regions, divided into six categories of 2 kb upstream sequences, 5’UTRs, coding sequences (cds), introns, 3’UTRs and 2 kb downstream sequences, was determined based on the EL10_1.0 sugar beet genome annotation file (NCBI accession: GCF_002917755.1), using AGAT tool v0.7.0^51^ and a custom Python script^40^.

### Experimental verification of MITE insertion sites identified by PopoolationTE2

12 MITE insertion sites, located in introns, were selected for validation. For PCR, site-specific primers were designed using Primer3^52^ and Primer-BLAST^53^ (Table S2 in Supplementary Materials 2). Amplification was carried out in a 10 μL total volume containing 20 ng of genomic DNA, 0.5 μM each of forward and reverse primer, 0.25 mM of each dNTP (Thermo Fisher Scientific), 0.5 U Taq DNA polymerase (Thermo Fisher Scientific) and 1 × Taq buffer. The PCR amplifications were performed in an Eppendorf MasterCycler Gradient using the following thermal profile: 94 °C (120 s), 30 cycles of 94 °C (30 s), 57 °C (30 s), 68 °C (120 s) and a final step of 68 °C (600 s). PCR products were separated in 1% agarose gels run in 1 × Tris–borate-EDTA buffer (pH 8.0) at a constant current of 5 V/cm for approximately 2 h, stained with ethidium bromide (0.2 mg/mL), and analysed using the GelDoc-It imaging system (UVP). GeneRuler 100 bp + DNA Ladders (Thermo Fisher Scientific) were used to determine product sizes for each locus.

### Determination of differentially expressed genes in homozygosity bins and MITEs associated with DEGs

Finally, the results of differential expression analysis were compared with genes from the sugar beet reference genome annotation identified in homozygosity bins and MITE insertions (Fig. S1 in Supplementary Materials 1). For each bin, three plants carrying homozygous gene-associated insertions and three plants carrying corresponding empty sites were selected, and the expression levels of each MITE-associated gene were compared in the contrasting trios. Firstly, DEGs were matched to all genes in homozygosity intervals for the same sample sets. Subsequently, using a custom Python script^40^, MITEs identified in the genic regions were matched to DEGs determined in homozygosity bins. Heatmap visualisation of DEGs and associated MITEs was performed using the matplotlib and seaborn libraries in Python^40^.

A permutation test was performed to assess whether the observed association between MITE insertions and DEGs was higher than would be expected by chance. Firstly, the observed number of DEGs associated with MITEs was calculated, considering overlap or location within ± 2 kb of at least one MITE copy. Then, DEG labels were randomly reassigned 1,000 times across all genes, and the number of MITE-associated DEGs was recalculated in each iteration. The empirical *p*-value was computed as a fraction of permutation results where the number of MITE/DEG associations was equal to or greater than the observed value:

$$p = \frac{{\sum\limits_{i = 1}^{N} {1\left( {P_{i} \ge P_{{{\mathrm{obs}}}} + 1} \right)} }}{N + 1}$$

where: *P*_obs_ – the observed number of DEGs associated with MITEs; *P*_i_ – the number of DEGs associated with MITEs from permutation; N – the number of permutations.

## Results

### Abundance and genomic localisation of MITEs in the sugar beet reference genome

**Fig. 1:**
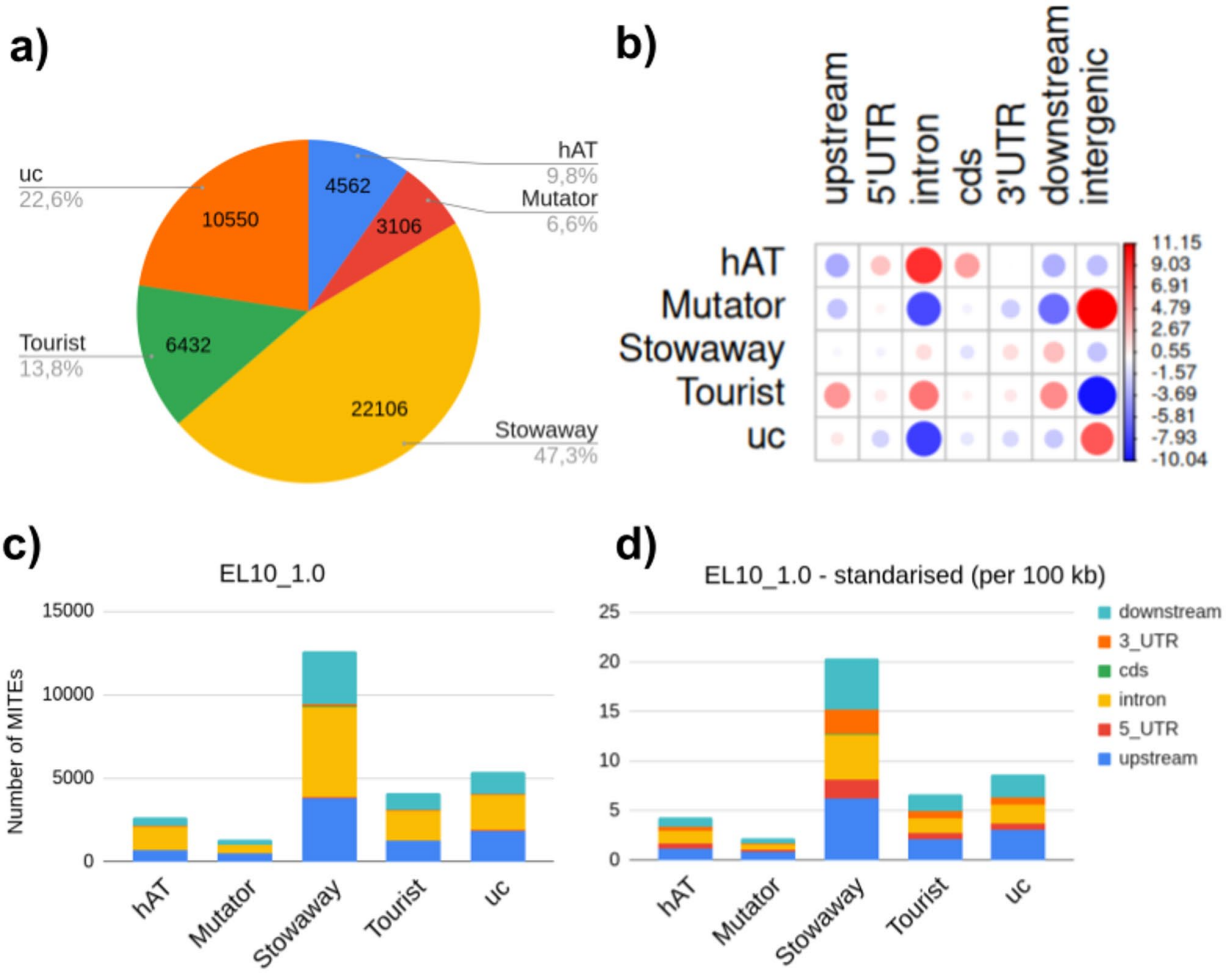
MITEs identified in EL10_1.0 sugar beet reference genome: **a** MITE copy numbers. **b** Relative abundance of MITE copies attributed to superfamilies in different genomic regions. The colour scale reflects deviations from average values (*p*-value < 2.2 × 10^–16^). Circle size is proportional to the contribution of each test to the total Pearson chi-squared score. **c** The number of MITE copies within genic regions (in up- and downstream regions, CDS, introns and UTRs). **d** The number of insertions per 100 kb (standardised to the cumulative length of each region). ‘uc’ stands for unclassified MITEs.

We identified MITE families in two sugar beet reference genomes (RefBeet, GCF_000511025.2; EL10_1.0, GCF_002917755.1), and divided them into groups based on their relationships to class II TE superfamilies, as revealed by their TIR and TSD similarity. We used consensus sequences representing each family to identify individual copies along the EL10_1.0 reference assembly of the sugar beet genome (File S3 from Supplementary Materials). In total, MITEs belonging to 147 families were estimated to occupy about 2.8% of the sugar beet EL10_1.0 genome (46,756 copies spanning 14.94 Mb, Fig. 1a, Table 1, Table S3 in Supplementary Materials 2). The largest genome fraction was attributed to *Stowaways* (5.07 Mb), followed by unclassified elements (4.3 Mb). *Tourists* and *hAT*-like MITEs occupied 2.27 and 2.07 Mb, respectively. *Mutators* were the sparsest superfamily (1.24 Mb, Table 1). More than half of all MITEs (26,141 copies; 56%) were inserted within genic regions, defined as 2 kb upstream and downstream of genes, and including the gene body (Table S4 in Supplementary Materials 2). *Stowaways* constituted the highest number of MITEs, accounting for nearly half of all identified copies (22,106, 47.3%) and distributed among 38 families. The largest family, comprising 2,564 copies, was attributed to that group. *Stowaway*-like elements were relatively enriched downstream from genes and in introns. *Tourists* accounted for around 14% of copies (6,432), distributed among 20 families, with the most abundant family comprising 1,294 copies. *Tourist*-like elements were more frequently positioned in introns. In particular, *Tourists* were relatively more enriched upstream and downstream of genes than the other MITE groups. *hAT*-like elements represented approximately 10% of copies (4,562), divided into 28 families. The largest family in this group consisted of 487 copies. *hAT*-like MITEs were present mainly in introns, as well as in coding regions and 5’UTRs. The least abundant MITE superfamily was *Mutator* (ca. 7%, 3,106, 24 families), with the most numerous family comprising 497 copies. MITEs belonging to that group were mainly positioned in intergenic regions (Table S4 in Supplementary Materials 2, Fig. 1a, b). Approximately 23% of copies (10,550) were reported as unclassified MITEs (‘uc’), belonging to 37 families. The most numerous family in this group consisted of 1,958 copies. Unclassified MITEs were present mainly in intergenic regions.

**Table 1:** Abundance of MITEs identified in the EL10_1.0 reference sugar beet genome and in 12 plants form two F2 families, P1 and P4.

MITE superfamilies	EL10_1.0	P1	P4
*Genome coverage [Mb]*			
*hAT*	2.07	3.02 ± 0.06**	3.02 ± 0.08
*Mutator*	1.24	1.50 ± 0.03	1.48 ± 0.03
*Stowaway*	5.07	4.60 ± 0.09	4.58 ± 0.12
*Tourist*	2.27	2.87 ± 0.06	2.92 ± 0.09
uc*	4.30	5.05 ± 0.09	4.98 ± 0.15
Total	14.94	17.04 ± 0.32	16.97 ± 0.46
*% of Genome*			
*hAT*	0.38	0.56 ± 0.01	0.56 ± 0.01
*Mutator*	0.23	0.28 ± 0.01	0.28 ± 0.01
*Stowaway*	0.94	0.85 ± 0.02	0.85 ± 0.02
*Tourist*	0.42	0.53 ± 0.01	0.54 ± 0.02
uc*	0.79	0.93 ± 0.02	0.92 ± 0.03
Total	2.76	3.15 ± 0.06	3.14 ± 0.08

* unclassified MITEs.

** mean ± standard deviation.

The number of copies adjusted for the cumulative length of each defined genic context indicated a nearly twofold enrichment of *Mutators* and unclassified elements upstream from genes, as compared to introns. Considering this standardisation, about 1.5 times as many insertions located in upstream regions were recorded for *Tourists* and *Stowaways* (Fig. 1c, d).

MITE density was similar across all EL10_1.0 chromosomes, ranging from 8.52 for Chr. 1 to 9.55 per 100 kb for Chr. 3 (average: 9.00; Table S5 in Supplementary Materials 2; Fig. S3 in Supplementary Materials 1). The highest number of MITEs was identified on the Chr. 6 (5,64), while the fewest MITE copies were found on chromosome 9 (4,61) (Fig. S2a in Supplementary Materials 1). Most chromosomes showed increased MITE densities in the distal regions of chromosome arms, while on Chr. 9 a high-density peak was observed closer to the centre of the chromosome (Fig. S3 in Supplementary Materials 1).

### Abundance and genomic localisation of MITEs in the two F2 populations of sugar beet

For WGS, we obtained more than 118 M and 100 M read pairs per sample, for P1 and P4, respectively. More than 96% (93.42–97.71%, mean = 96.34% and 94.51–99.09%, mean = 96.85%, for P1 and P4, respectively) uniquely mapped to the reference sugar beet genome (EL10_1.0, GCF_002917755.1) and were used for mining MITEs and SNP calling (Table S1 in Supplementary Materials 2).

**Fig. 2:**
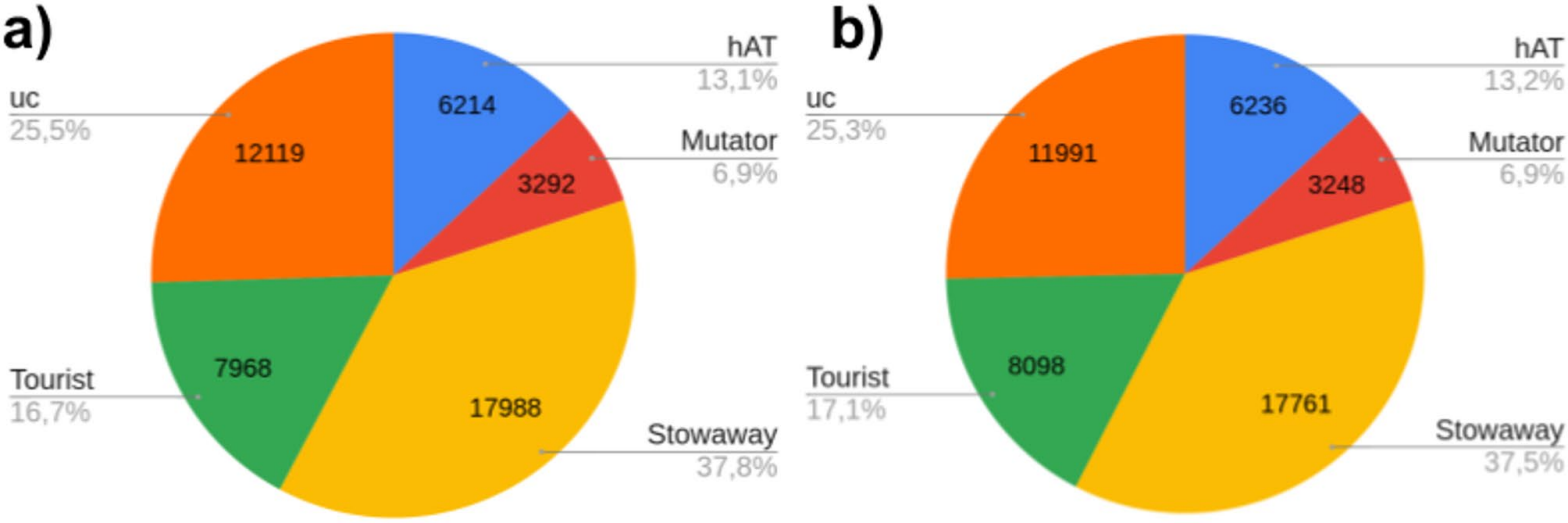
Average MITE copy numbers for 12 genomes among P1 (**a**) and P4 (**b**) sugar beet F2 families. ‘uc’ stands for unclassified MITEs.

In total, 60,426 and 61,906 MITE copies were identified in 12 genomes of *B. vulgaris* from each of the two F2 families, P1 and P4, ranging from 46,503 to 49,075 (mean = 47,581) and from 45,779 to 49,398 (mean = 47,335) copies per individual genome, respectively. MITEs were estimated to occupy on average 3.15% of the sugar beet genome in both F2 families, considering the EL10_1.0 reference genome assembly length (Table 1; Table S4-S9 in Supplementary Materials 2). The largest genome fraction was attributed to unclassified elements (approximately 5 Mb) and *Stowaways* (about 4.6 Mb), followed by *hAT*-like elements (3.02 Mb) and *Tourists* (approximately 2.9 Mb), meanwhile *Mutators* represented the least part of the genome (about 1.5 Mb) in both F2 families (Table 1). The distribution of MITEs from different superfamilies followed a similar pattern to the reference genome in both segregating populations (Fig. 2). The highest average number of MITEs was attributed to the *Stowaway* superfamily, accounting for over a third of all identified copies (17,988, 37.8% in P1 and 17,761, 37.5% in P4). Approximately 25–26% of copies were reported as unclassified MITEs (‘uc’), while *Tourists* and *hAT*-like elements grouped ca. 17% and 13% copies, respectively. The least numerous MITE superfamily was *Mutator* (ca. 7%) (Table 1, Fig. 2, Fig. S5a, b in Supplementary Materials 1, Table S4 in Supplementary Materials 2).

MITE density patterns in both F2 families generally followed that observed in the reference genome, averaging 11.63 and 11.92 per 100 kb in P1 and P4, respectively (Fig. S3 in Supplementary Materials 1). The highest density was observed on Chr. 3 in both F2 families (12.86 for P1 and 13.00 for P4), whereas the lowest values were noted on Chr. 6 in P1 (11.01) and on Chr. 1 in P4 (11.17; Table S5 in Supplementary Materials 2). The MITE copy number was similar for all sugar beet chromosomes in both F2 families (Fig. S2b, c in Supplementary Materials 1). The highest number of MITEs was identified on Chr. 5 (7,352 for P1 and 7,699 for P4), while the fewest MITE copies were found on Chr. 9 (5,802 for P1 and 6,440 for P4). The calculated proportion of homo- to heterozygous insertion sites indicated an underrepresentation of heterozygotes in each group of MITEs (Fig. S6, S7 in Supplementary Materials 1; Table S10, S11 in Supplementary Materials 2).

We further validated the results of in silico predictions for MITE copies residing in 12 randomly chosen insertion sites localised within introns, using a PCR reaction according to the method described by Stelmach et al.^52^ (Table S2 in Supplementary Materials 2). Approximately 73% of PCR-verified variants matched the bioinformatic predictions, while 20% pointed at occasional discrepancies in the assumed interpretation of the threshold values of the frequency calculated by PopoolationTE2 as indicating a homozygote, where PCR verification revealed the presence of a heterozygous insertion or vice versa (Fig. S8 in Supplementary Materials 1). Nevertheless, it should be noted that the pipeline correctly predicted the presence of MITE insertions in all of these cases. Incorrect bioinformatics prediction regarding the presence or absence of an insertion occurred for less than 6% of the analysed sites (Table 2). This demonstrated that the applied in silico strategy reliably identified MITE insertional polymorphisms and allowed for credible selection of plants with homozygous MITE insertions vs. those with homozygous empty sites, for comparison with the results of differential gene expression.

**Table 2:** PCR verification of in silico results for 12 MITE insertion sites in the sugar beet genome.

Comparison of in silico/PCR results	Accession/insertion sites combination	
	Number	%
homozygous empty/homozygous empty	26	9.85
homozygous occupied/homozygous occupied	131	49.62
heterozygous/heterozygous (empty + occupied)	35	13.26
Total correct calls	192	72.73
heterozygous/homozygous occupied	37	14.02
homozygous occupied/heterozygous	15	5.68
homozygous empty/heterozygous	6	2.27
homozygous empty/homozygous occupied	5	1.89
homozygous occupied/homozygous empty	5	1.89
Total incorrect calls	68	25.76
No amplification	4	1.52
Total	264	100

**Fig. 3:**
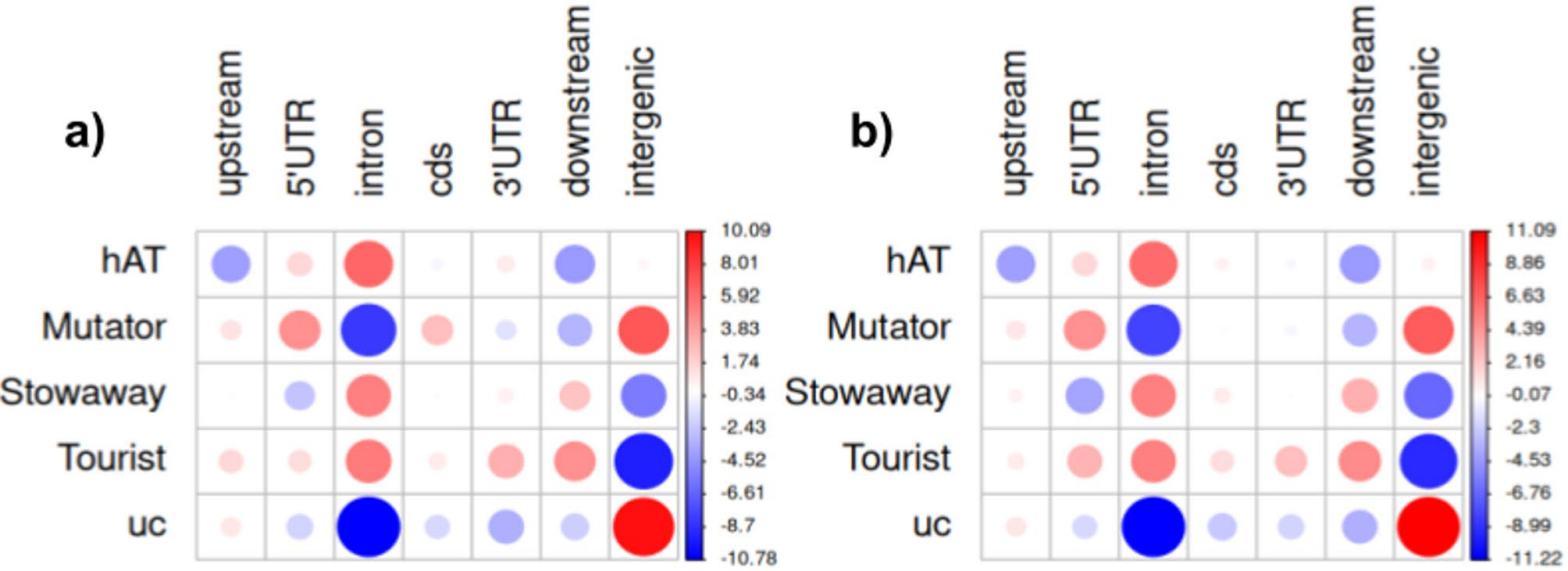
Relative abundance of MITE copies attributed to superfamilies in different genomic regions for the two sugar beet F2 families, P1 (**a**) and P4 (**b**). The colour scale reflects deviations from average values (*p*-value < 2.2 × 10^–16^). Circle size is proportional to the contribution of each test to the total Pearson chi-squared score. ‘uc’ stands for unclassified MITEs.

**Fig. 4:**
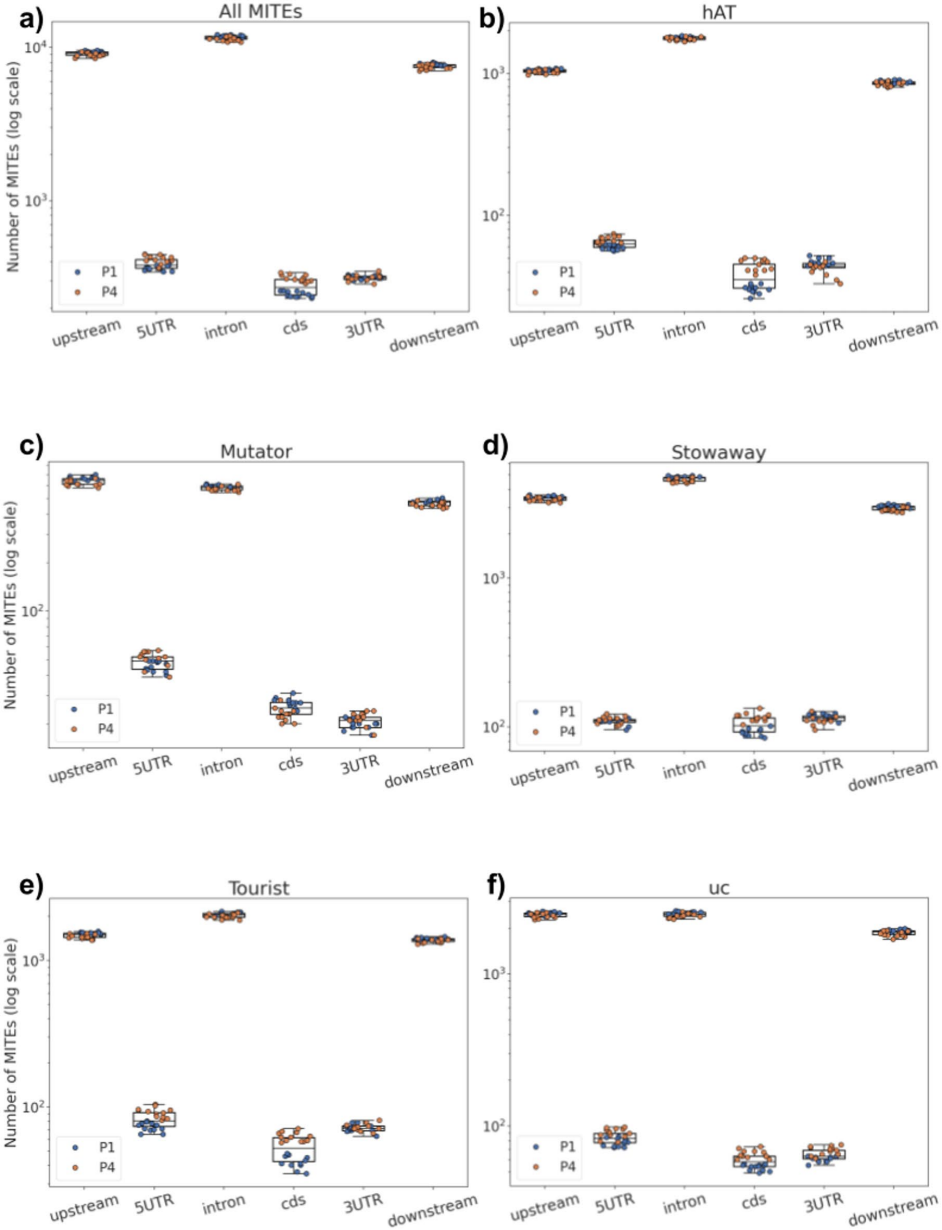
Boxplots showing the number of MITE copies in different genomic regions, illustrating variability among the 12 genomes within the two sugar beet F2 families, P1 and P4: all MITE copies **a**, and attributed to superfamilies: *hAT* **b**, *Mutator* **c**, *Stowaway* **d**, *Tourist* **e**, and uc **f**. ‘uc’ stands for unclassified MITEs.

The MITE superfamilies differed concerning the number of families, copy number, and genomic localisation. However, similar patterns were noted between the P1 and P4 regarding these parameters (Fig. 2, Fig. S4, S5, S9 in Supplementary Materials 1). Over 60% of all identified MITEs in both F2 families (37,406 and 38,191 copies for P1 and P4, respectively) were inserted within genic regions, defined as 2 kb upstream and downstream of genes, including the gene body (Table S4 in Supplementary Materials 2). *Stowaways* constituted the most abundant group of MITEs in both P1 and P4, distributed among 38 families. The largest family, comprising over 2,500 copies in both F2 populations, was assigned to that group. *Stowaway*-like elements were relatively enriched in introns and downstream from genes. Unclassified MITEs were the second most abundant group and were found mainly in intergenic regions. *Tourists* were the least diverse superfamily, distributed among 20 families. *Tourist*-like elements were more frequently positioned in introns, upstream and downstream from genes and in UTRs. In particular, *Tourists* were relatively more enriched in 3’UTRs than the other MITE groups. *hAT*-like MITEs belonged to 28 families, and the largest one within that group comprised ca. 650 elements. *hAT*-like elements were present mainly in introns and 5’UTRs. Sugar beet *Mutator* MITEs were the least numerous, with the largest family comprising ca. 420 copies. MITEs belonging to that group were mainly positioned in intergenic regions. *Mutator*-like elements were also relatively more frequently located in 5’UTRs than in the other MITE groups (Figs. 3 and 4, Table S4 in Supplementary Materials 2).

The number of copies adjusted for the cumulative length of each defined genic context indicated a twofold enrichment of *Mutator* and unclassified elements upstream from genes, as compared to introns. Considering this standardisation, about 1.5 times as many insertions located in upstream regions were recorded for *Stowaways* and *Tourists* and downstream for *Mutators* and unclassified elements (Fig. S5 in Supplementary Materials 1).

### Polymorphisms in the segregating populations

We identified 7,097,431 and 6,930,299 polymorphisms for the P1 and P4 sugar beet F2 families, respectively, with positive quality control results, less than 20% missing data, of which 5,416,895 (P1) and 5,273,910 (P4) were single nucleotide polymorphisms (SNPs). After indel filtering, we retained 5,076,044 and 4,969,501 SNPs, respectively (Table S12 in Supplementary Materials 2). The average number of SNPs per plant was 4,272,718 and 4,095,800 for the P1 and P4 populations, respectively (Table S13 in Supplementary Materials 2).

Analysis of the abundance of polymorphisms showed that the highest variability was observed for chromosomes 6 and 5 in P1 (11.77 and 11.55 polymorphisms/kb, respectively) and for chromosomes 5 and 8 in P4 (10.10 and 10.01 polymorphisms/kb, respectively). The lowest variability was recorded for chromosome 2 in both F2 families (6.69 and 8.08 polymorphisms/kb for P1 and P4, respectively) and chromosome 1 in P1 (6.69 polymorphisms/kb) (Table S14 in Supplementary Materials 2).

### Identification of homozygosity regions

We identified 4,463 and 5,615 homozygosity regions for P1 and P4, respectively (Table 3). Approximately 40–46% of all homozygosity bins were longer than 1 kb (Table S15 in Supplementary Materials 2). The highest number of these regions for both F2 families was identified on chromosome 6, while the smallest was on chromosome 2 (Table 3). These values correspond with the results of the distribution analysis of polymorphisms (Table S14 in Supplementary Materials 2).

**Table 3:** Homozygosity bins identified in sugar beet F2 families P1 and P4.

Chromosome number	P1			P4		
	All bins	Bins with genes	Genes in bins	All bins	Bins with genes	Genes in bins
1	332	154	1,763	744	288	1,690
2	150	49	264	366	105	509
3	375	104	960	720	378	2,264
4	584	188	810	582	170	610
5	708	191	613	393	106	966
6	776	196	942	1,207	293	838
7	522	117	539	741	362	1,901
8	454	139	1,056	446	96	303
9	562	285	1,801	416	217	2,356
SUM	4,463	1,423	8,748	5,615	2,015	11,437

Comparison of the location of homozygosity regions with the gene positions from the reference sugar beet genome annotation (EL10_1.0, GCF_002917755.1) enabled the identification of 8,748 genes within 1,423 bins in P1 and 11,437 genes in 2,015 bins in P4 (Table 3). The highest number of bins containing genes was identified on chromosome 9 in P1 (285 bins with 1,801 genes), while for P4 the highest number of bins with genes was on chromosome 3 (378) and most of the genes within the bins were found on chromosome 9 (2,356) (Table 3).

We further typed regions of homozygosity containing 20 or more genes to select sample sets for differential expression (DE) analysis. We found 106 and 135 homozygosity bins with 20 or more genes in P1 and P4, respectively (Table 4). The most abundant representation of these regions was observed on chromosome 1 in P1 and on chromosome 9 in P4 (Table S16 in Supplementary Materials 2).

**Table 4:** Number of homozygosity bins for sugar beet F2 families P1 and P4 spanning genes from the reference annotation.

Number of bins	P1	P4
All	4,463	5,616
With no genes	3,040	3,601
> = 1 genes	1,423	2,015
< 20 genes	1,243	1,880
> = 20 genes*	106	135
> = 30 genes	65	75
> = 50 genes	30	34
> = 70 genes	12	16
> = 100 genes	4	7

* threshold for selecting sample sets for DE analysis.

### Determination of differentially expressed genes in homozygosity bins and associated MITE insertions

RNAseq resulted in approximately 38 M and 34 M read pairs per sample in the P1 and P4 populations. The percentage fraction of correctly mapped read pairs ranged from 94.1% to 96.09% in the P1 F2 family and from 95.06% to 96.69% in the P4 (Table S1 in Supplementary Materials 2). These results were used to identify differentially expressed genes (DEGs).

**Fig. 5:**
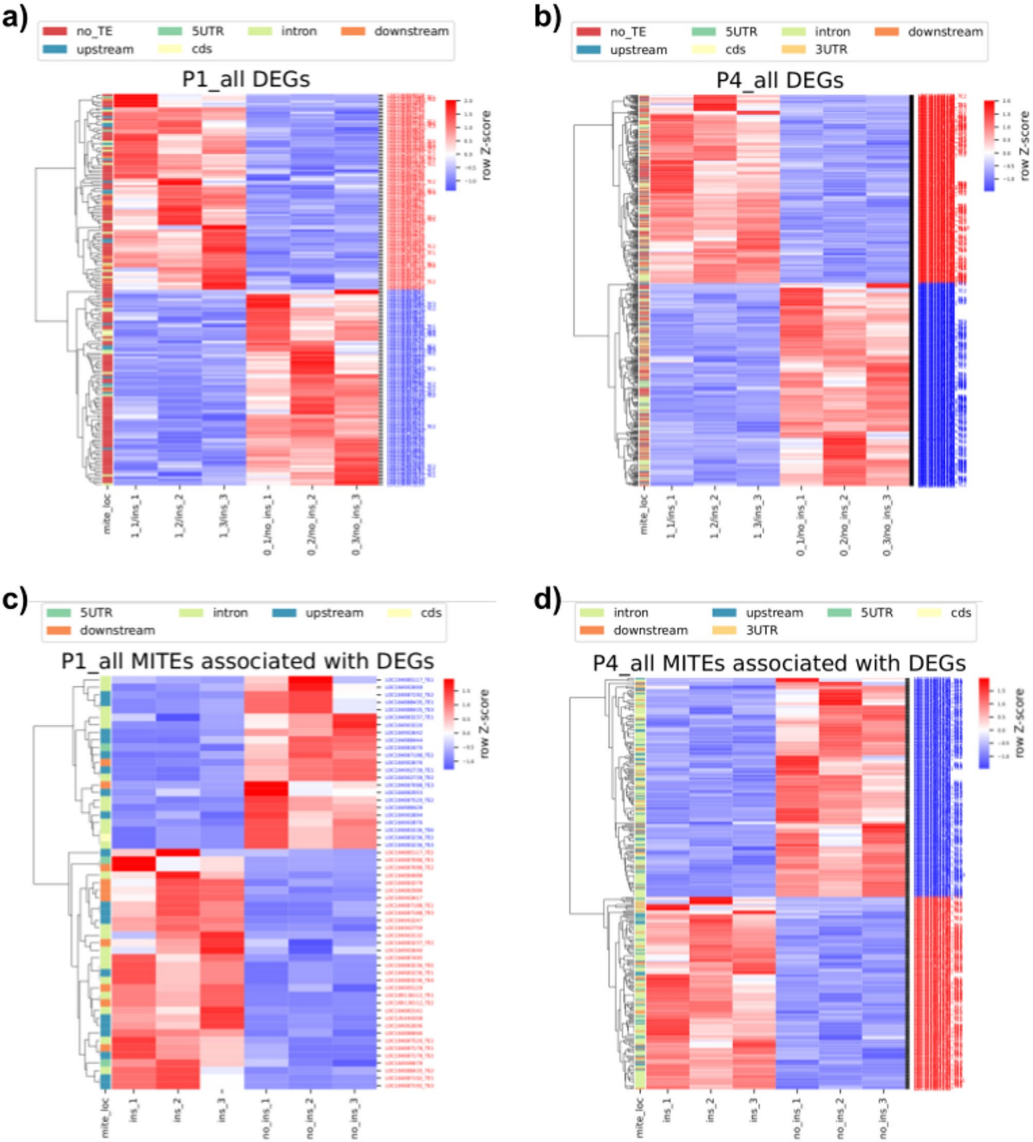
Heatmaps representing up- and downregulated DEGs (gene names in red and blue, respectively) in the P1 and P4 sugar beet F2 families: **a**, **b** all DEGs (associated and not associated with MITEs), identified in homozygosity bins; **c**, **d** only DEGs associated with MITEs, depending of the presence/absence status of the MITE copy associated with DEGs. Normalised expression of genes from three plants in homozygosity bins only carrying MITE copies associated with those genes and three plants carrying homozygous empty sites **c** and **d** or with and without MITE copies **a** and **b** was used to develop heatmaps. Positions of MITE insertion sites relative to the genes (and lack of MITE insertion for **a** and **b** are marked by different colours. Row z-score represents the normalised expression of DEGs.

96 and 92 sample sets for DE analysis were selected for P1 and P4 and matched to regions of homozygosity among all identified intervals. Within the genomic intervals encompassing regions of homozygosity, we identified 58 and 319 MITE copies belonging to 53 and 89 families, in the vicinity of 36 and 170 DEGs in P1 and P4, respectively (Fig. 5, Fig. S10 in Supplementary Materials 1, Table S19-S21 in Supplementary Materials 2). Most MITEs associated with DEGs were classified as *Stowaways*, while the fewest were attributed to *Mutators* in both sugar beet F2 families (Fig. 7). In absolute numbers, about half of the copies, from all MITE groups except *Mutators*, were located in introns of DEGs. Copies of *hAT*-like, *Stowaway*, *Mutator* and unclassified MITEs were also observed relatively frequently upstream from DEGs (Fig. 7a, c). The number of copies adjusted for the cumulative length of each defined genic segment indicated twofold enrichment of *hAT*-like copies in 5′ UTRs, as compared to introns, in both F2 families (Fig. 7b, d). Noticeably, *hAT*-like elements were enriched in the 5’UTR of DEGs compared to the overall distribution of MITEs in the sugar beet genome. In turn, *Stowaways* and *Tourists* appeared less frequently downstream from DEGs, while *Mutator*-like elements were scarce in introns of either DEGs or the overall MITE distribution. (Figs. 3, 4, 7).

**Fig. 6:**
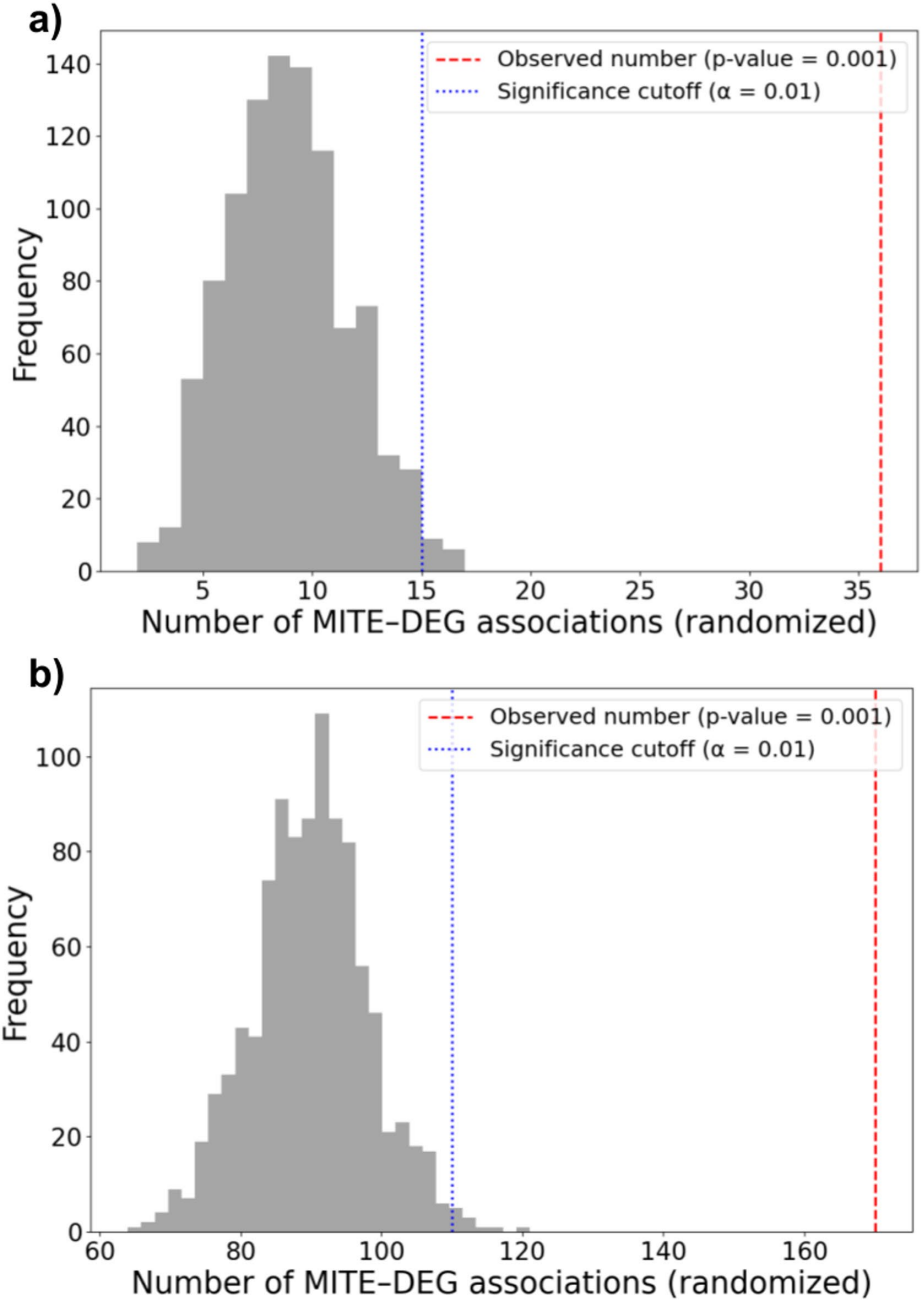
Permutation analysis of MITE/DEG associations in the regions of homozygosity in two F2 populations of sugar beet, P1 (**a**) and P4 (**b**) DEGs associated with MITEs are overrepresented. Red line—the number of observed associations, blue line—significance cutoff at *α* = 0.01; histograms are derived from 1000 permutations.

**Fig. 7:**
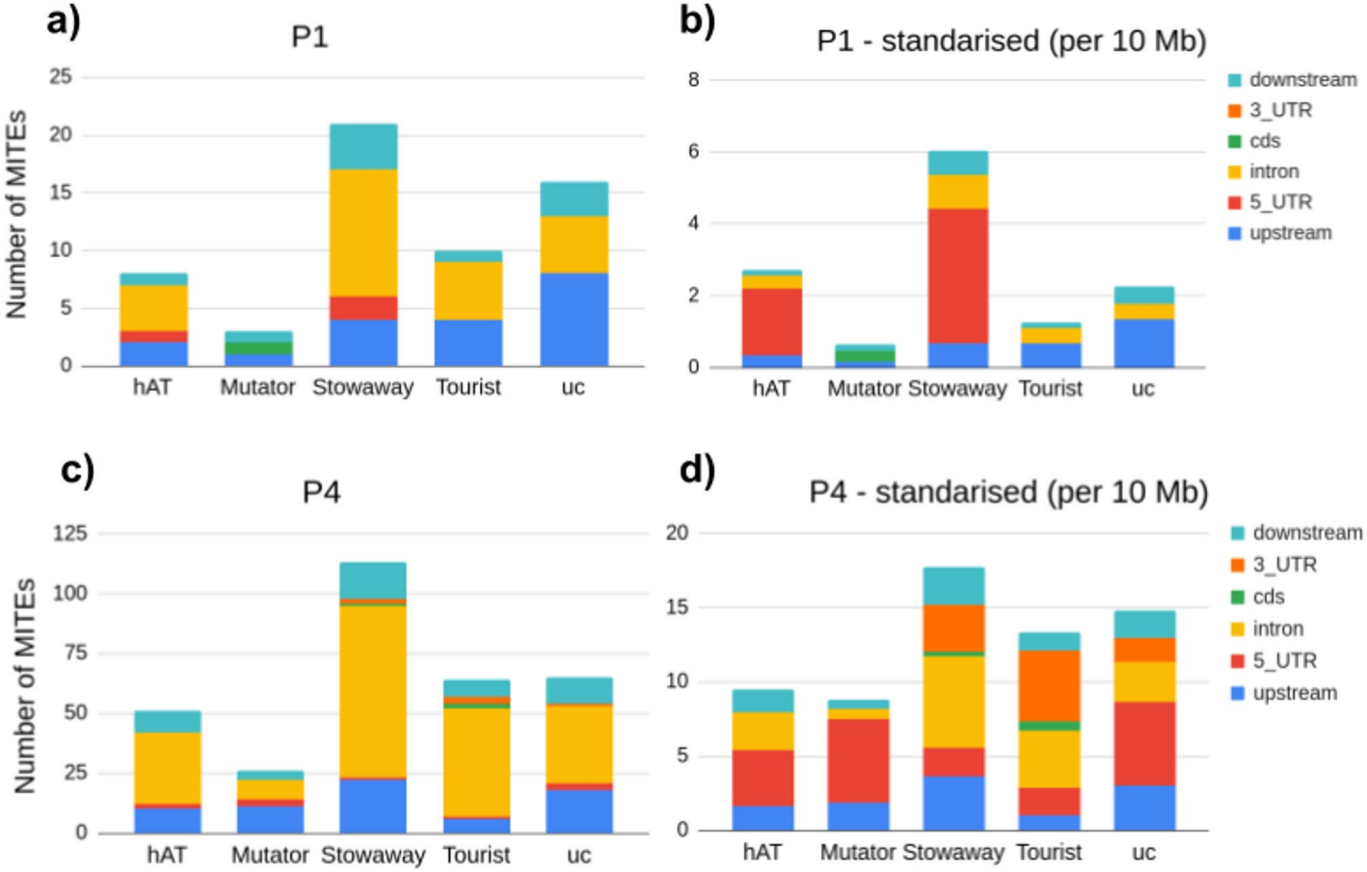
The number of polymorphic MITE insertions associated with DEGs within the regions of homozygosity in the two F2 populations of sugar beet. The number of copies present in up- and downstream regions, CDS, introns and UTRs in the F2 families P1 (**a**) and P4 (**b**). The number of insertions per 10 Mb (standardised to the cumulative length of each region) in the P1 (**c**) and P4 (**d**). ‘uc’ stands for unclassified MITEs.

Of all genes positioned in homozygosity bins, 2.86% (163 of 5,698) and 4.80% (441 of 9,192) were DEGs in P1 and P4, respectively (Fig. 5a, b; Table S17, S18 in Supplementary Materials 2). However, a much higher fraction of genes positioned in homozygosity bins and associated with MITE insertions were differentially expressed, namely 14.75% (36 of 244) and 9.82% (170 of 1,731) in P1 and P4, respectively, (Table S19 in Supplementary Materials 2). Thus, we observed a two- to four-fold increase in the frequency of DEGs among genes associated with polymorphic MITE insertions as compared to those not associated with MITEs. Permutation tests further supported the observation that MITEs were non-randomly associated with DEGs in both F2 families (Fig. 6).

**Fig. 8:**
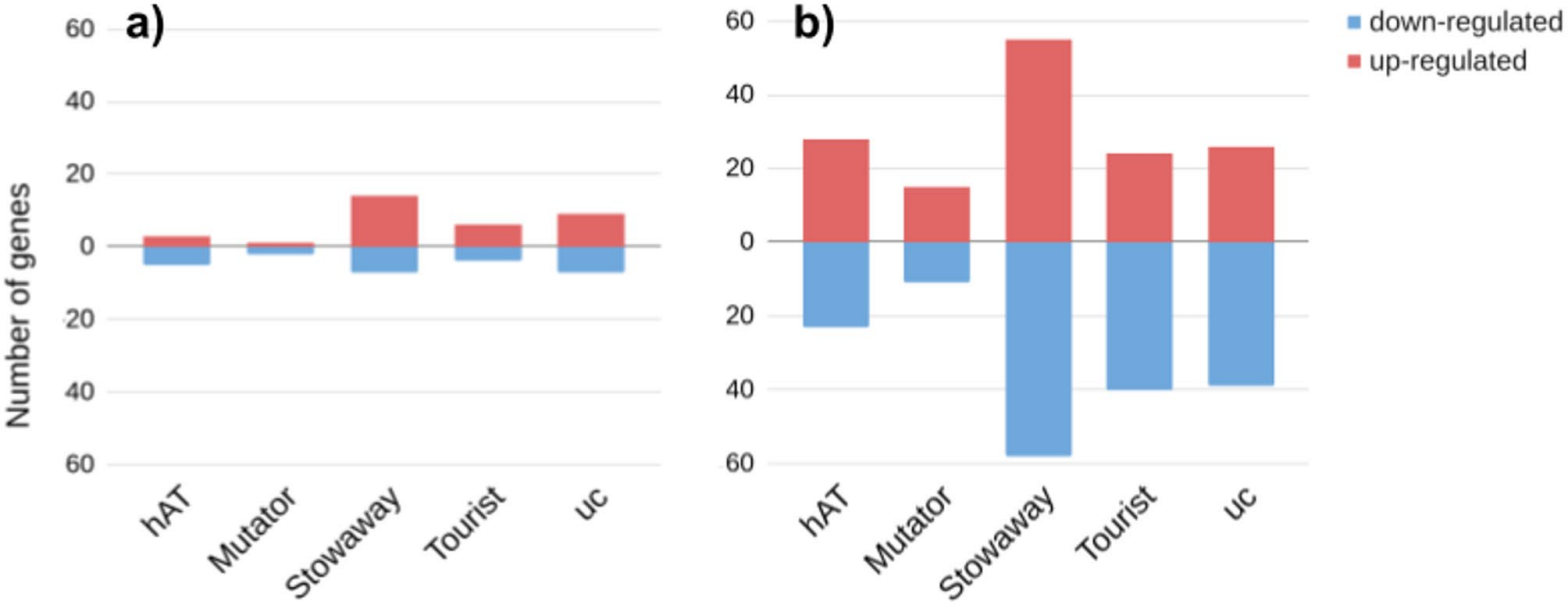
Up- and downregulated DEGs (red and blue bars, respectively) associated with MITE insertions in P1 (**a**) and P4 (**b**) sugar beet F2 families.

As with all DEGs identified in regions of homozygosity, the proportion of upregulated to downregulated genes associated with MITE copies was close to 1:1 in both F2 families, however, differences were observed within the MITE groups (Figs. 7c, d and 8). In P1, there were more than twice as many upregulated genes as downregulated genes associated with copies of *Stowaways*, while in P4 such bias was not observed. In contrast, *Tourist* copies in P4 were recorded twice as often in association with downregulated genes compared to upregulated genes (Fig. 8).

**Fig. 9:**
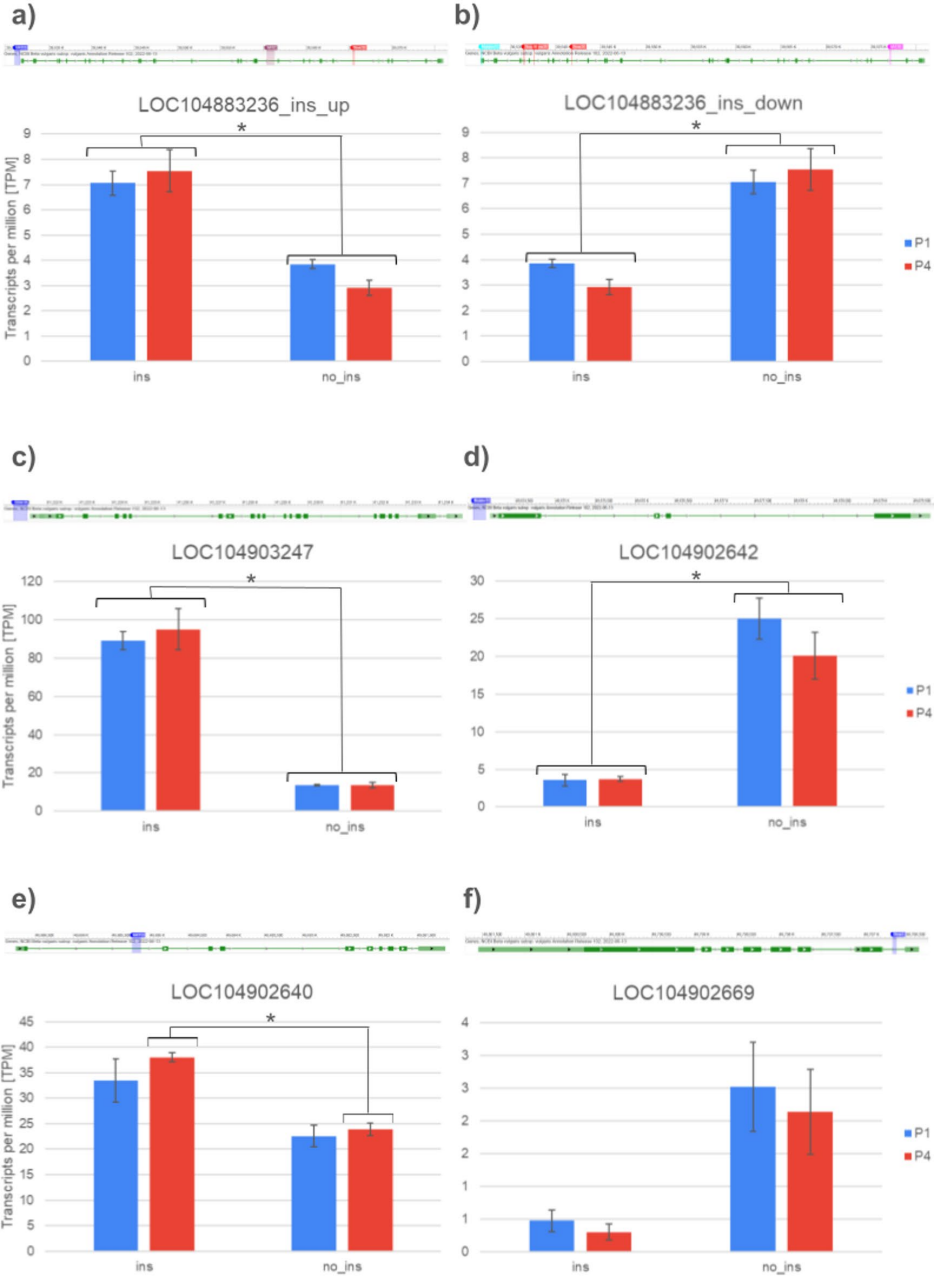
Examples of differentially expressed genes associated with MITE insertions segregating within both sugar beet F2 populations, P1 and P4. LOC104883236 is associated with multiple MITE copies, three in the upregulated variant **a** and four in the downregulated variant **b**; LOC104903247 (upregulated) **c** and LOC104902642 (downregulated) **d** are associated with MITE copies inserted upstream; while LOC1004902640 (upregulated) **e** and LOC104902669 (downregulated) **f** carry intronic MITE insertions. RNAseq was used to quantify expression levels, whiskers show SE; significant differences at *p* = 0.05 are indicated.

Five DEGs associated with the same MITE copies were identified in both F2 families on Chr. 9 (Table S22 in Supplementary Materials 2). LOC104883236, annotated as a hypothetical protein, carried three or four MITE copies, depending on the haplotype. Among them, a copy of *hAT13* was located upstream and associated with gene upregulation. The other four DEGs were associated with single MITE copies from different MITE families. *Stowaway18,* located upstream of LOC104903247, was associated with its higher expression in the roots. Interestingly, this gene encodes ferredoxin, an important enzyme involved in photosynthesis. In contrast, the presence of *Mutator18* upstream LOC104902642, encoding a hexamer DNA-binding protein (HEXBP), was associated with downregulation of the gene (Table S22 in Supplementary Materials 2). The remaining two genes carried intronic insertions of *hAT13* and *Stowaway3*, associated respectively with up- and downregulation of the respective genes, i.e. LOC104902640, encoding DNA-directed RNA polymerase IV and V subunit 4, and LOC104902669, encoding serine/threonine-protein kinase. Notably, all five genes showed similar expression patterns in P1 and P4 (Fig. 9).

## Discussion

MITEs comprise one of the most numerous groups of transposons in plant genomes. Higher diversity and abundance of MITEs have been recorded in monocots compared to dicots^55^. Our results determined the MITE copy number equal to 46,756, divided into 147 families and spanning nearly 2.8% of the EL10_1.0 reference sugar beet genome assembly. Moreover, we identified on average 47,581 and 47,335 copies from the 147 MITE families in two F2 sugar beet populations, respectively, covering approximately 3.1% of the genome. These slight discrepancies in copy number and genome coverage may arise from the hybrid origin of the F2 families, reflecting a higher genetic diversity level. Nonetheless, sugar beet stands out from most other dicotyledonous species and could be mentioned among several species with a relatively small genome size, but containing a large number of diverse MITEs, with *Solanum lycopersicum*, *Cajanus cajan* and *Daucus carota* as the most prominent examples. In the tomato genome of 781 Mbp, 104 MITE families and 107,087 copies comprising 3.4% of the genome were identified. The 606 Mb-long pigeon pea genome was reported to contain 135,581 copies divided into 92 families, covering 5.1% of the genome^55^. Thus, although the MITE copy number in the sugar beet genome is generally lower, it shows similar genome coverage and diversity. However, 428 MITE families were estimated to occupy around 2% of the 473-Mb carrot genome (31,025 copies spanning 10.34 Mbp)^20^. Compared to the carrot, sugar beet has a comparable MITE copy number and genome coverage, but significantly less diversity in terms of MITE families.

Our results revealed that *Stowaways* constituted the most abundant MITE superfamily in the sugar beet genome, reaching nearly half and over one-third of all copies in the reference genome and F2 families, respectively. Their presence was identified on all nine chromosomes. Similar observations relate to the previously reported sugar beet MITEs group, comprising three families of *Stowaway*-like elements^27,28^. *Stowaways* have also been reported to be the most numerous MITE group in other plant genomes, such as rice^21^ or carrot^20^. However, *Arabidopsis thaliana* was found to have the most MITE copies belonging to the *hAT* superfamily among the previously classified MITE families. It should be noted that about 80% of the MITE copies identified in this species were reported as novel or unclassified^56^. In our results, approximately 25% of all copies in both the reference genome and the F2 families were reported as unclassified MITEs. It indicates a potential field for further research to provide a more detailed characterisation of these elements.

We demonstrated that about 60% of sugar beet MITEs (in either the reference genome or the P1 and P4 F2 families) are located in genic regions, possibly affecting expression levels of adjacent genes. They were distributed more densely in the distal, gene-rich regions of chromosome arms. A comparable fraction also comprised MITEs associated with genes in the genomes of rice and carrot^20,21^. Moreover, we observed that different MITE groups were enriched in different genic contexts, which may suggest their possible involvement in various regulatory mechanisms. Sugar beet *Stowaways* and *Tourists* were frequently present in introns and downstream from genes. *Tourists* were also often located upstream from genes and relatively more enriched in 3’UTRs than the other MITE groups in F2 families. *hAT*-like MITEs were present mainly in introns and 5’UTRs, while *Mutator*-like elements were significantly enriched in intergenic regions and relatively more frequently located in 5’UTRs than in the other MITE groups in F2 families. Similar observations were reported in the rice genome, where the *Stowaway* and *Tourists* were enriched in introns, and *Tourists* were also frequently present upstream from genes^21^. As in the carrot genome, *Tourist* elements were predominantly localised in UTRs and regions upstream or downstream from genes. In turn, *Stowaways* were frequently present in intergenic and upstream regions, while *Mutator*-like MITEs were enriched not only in intergenic segments but also in introns^20^. In potato, *Stowaway*-like elements were likewise often located in the promoter region of genes^57^.

MITEs can interact with genes in multiple ways. They are usually considered to downregulate gene expression^56,58^. Examples of epigenetic gene downregulation induced by MITE insertion in the promoter region in the maize genome have been reported^13,14^. Downregulation of genes by MITE-dependent alteration of methylation through RNA-directed methylation (RdDM) has also been described^14,24^. On the other hand, it has been demonstrated that a MITE insertion in the rice genome, localised upstream from a gene, could trigger its upregulation^15^. Our study revealed that a considerable share of DEGs (22 and 40% for P1 and P4) were associated with MITEs, particularly those from the *Stowaway* and *Tourist* families. Copies of *hAT*-like, *Stowaway*, *Mutator* and unclassified MITEs were also observed relatively frequently upstream from DEGs. However, no clear association was found between the MITE superfamily and the tendency for up- or downregulation of DEGs. We therefore attempted to focus on MITE insertions identified in the vicinity of the five DEGs segregating in both F2 families. Our results demonstrated that two MITE copies, belonging to *hATs* and *Stowaways*, located upstream of two different genes, were associated with upregulation, whereas another upstream *Mutator*-like element was found to be associated with a downregulated gene variant. In mulberry, the 564-bp MITE insertion was required for the activity of the promoter of the *ans* gene encoding anthocyanidin synthase, reprogramming the expression profile^16^. These examples, along with our findings, highlight the importance of further research into the potential multifaceted effects of MITE insertions on the expression of particular genes, which could differ with respect to positional effects and/or characteristics of individual MITE families or even copies, e.g. their ability to distribute CREs^59^.

Our outcomes suggest that MITEs may have regulatory functions in the sugar beet genome. We developed a curated sugar beet MITE repository, providing means for further analysis of MITEs and their effects on the structure and function of sugar beet genes and genomes. Besides the analysis of the functional role and impact on the genome evolution, MITE insertional polymorphisms can be utilised as molecular markers^54,60^.

## Conclusions

As in other plant species, MITEs in the sugar beet genome are abundant and diverse. Their widespread prevalence in the vicinity of genes indicates their possible contribution to the regulation of gene expression. MITEs from different superfamilies differ with respect to their genomic distribution and gene association. The identification and characterisation of MITEs in the sugar beet genome provides a basis for further research into their functional impact. It also facilitates the exploitation of MITE insertion polymorphisms to identify genetic factors associated with important agronomic traits in sugar beet.

## Supplementary Information

Below is the link to the electronic supplementary material.

Supplementary Material 1

Supplementary Material 2

Supplementary Material 3

## Data Availability

We used the previously published sugar beet reference genome assembly RefBeet and EL10_1.0, available at NCBI (GenBank acc. no. GCA_000511025.2 and GCA_002917755.1, respectively). DNAseq and RNAseq reads from 24 plants representing the two F2 families, P1 and P4 (12 plants each), were deposited in GenBank (acc. no. PRJNA1276275).
