# Supplementary material for: Miniature inverted repeat transposable elements in the genome of sugar beet and their impact on gene expression
Source: Sci Rep. 2025 Dec 19;16:1757. doi: 10.1038/s41598-025-32772-7 (PMC12804786; doi:10.1038/s41598-025-32772-7)
Supplement: Supplementary file 1 — Supplementary Material 1 [file 41598_2025_32772_MOESM1_ESM.pdf]

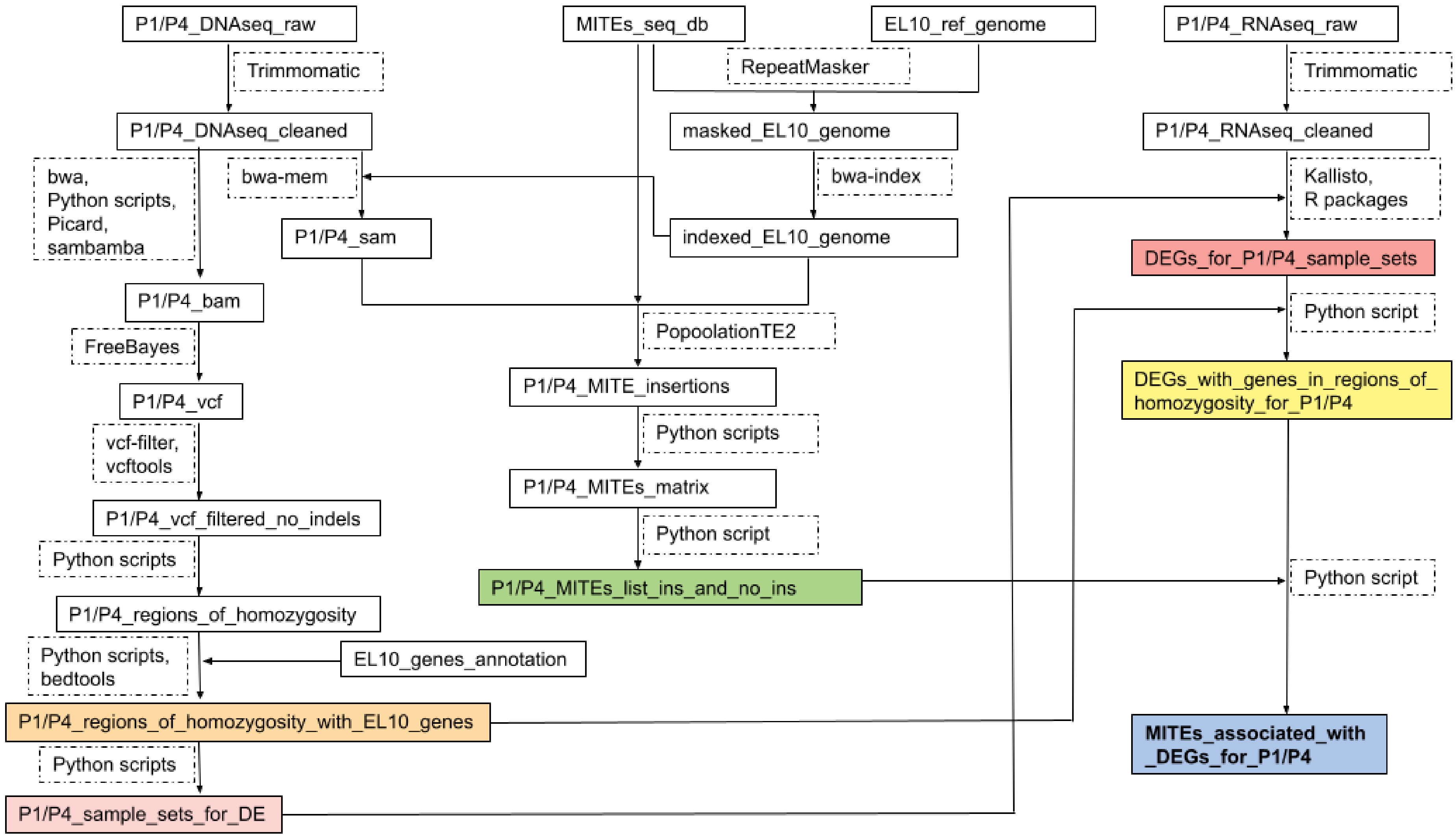

Fig. S1. Flowchart with the following steps: identification of MITEs in the sugar beet genome, determination of homozygosity regions, differential expression analysis and relating the results to the location of MITE insertions.

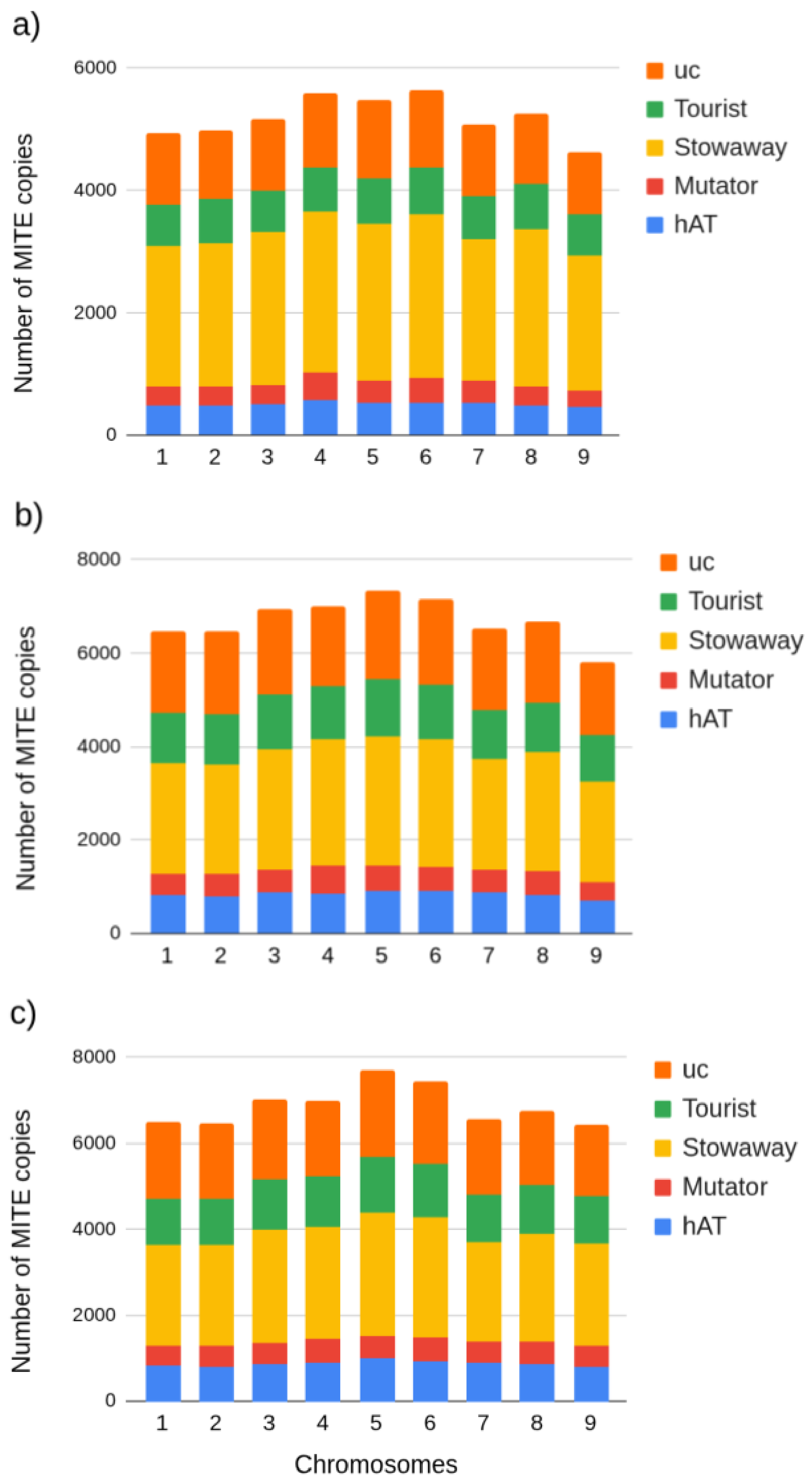

Fig. S2. MITEs abundance on chromosomes in the a) EL10_1.0 reference assembly of the sugar beet genome and each of 12 genomes in b) P1, and c) P4 F2 sugar beet families. ‘uc’ stands for unclassified MITEs.

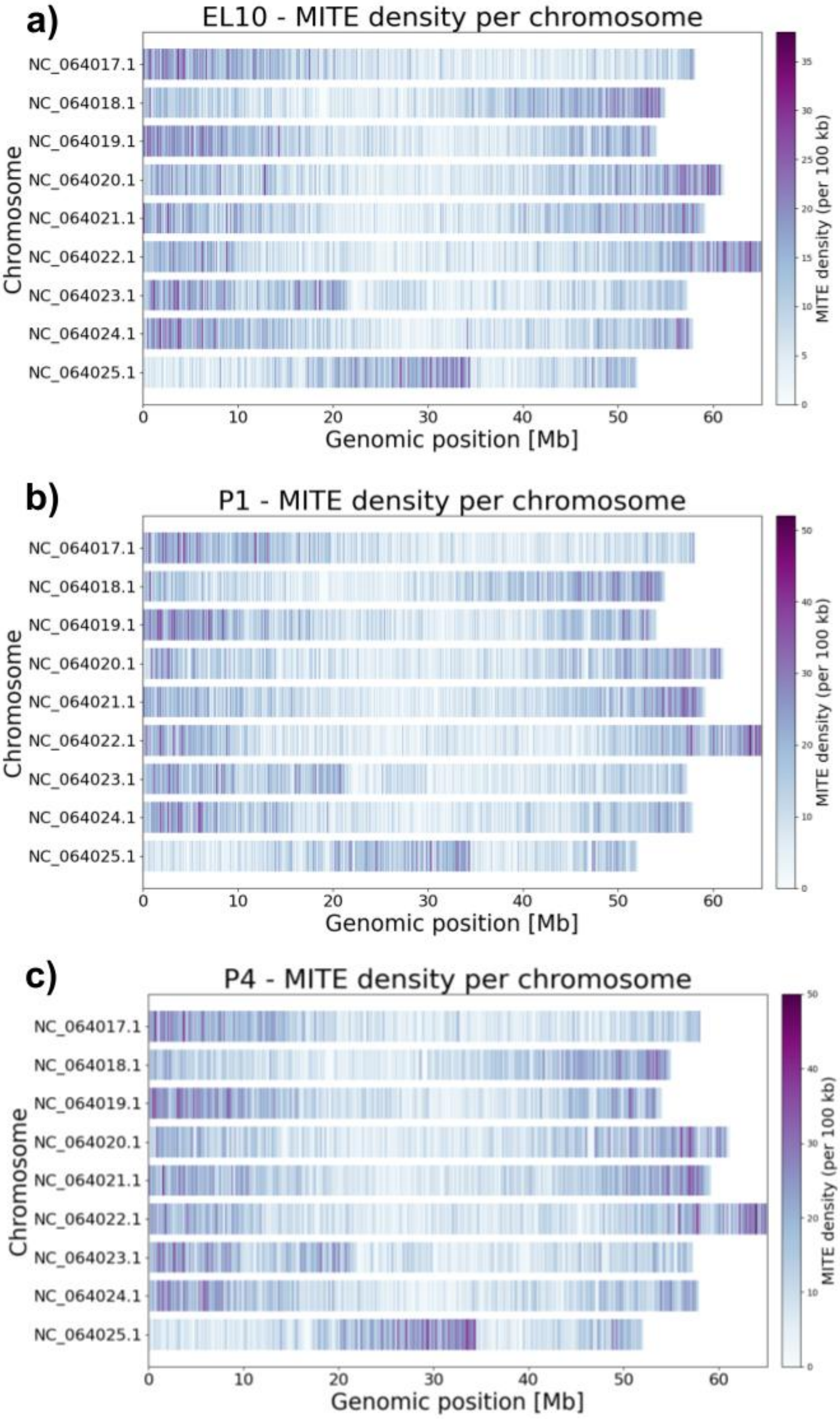

Fig. S3. MITEs density on chromosomes in the a) EL10_1.0 reference assembly of the sugar beet genome and each of 12 genomes in b) P1, and c) P4 F2 sugar beet families.

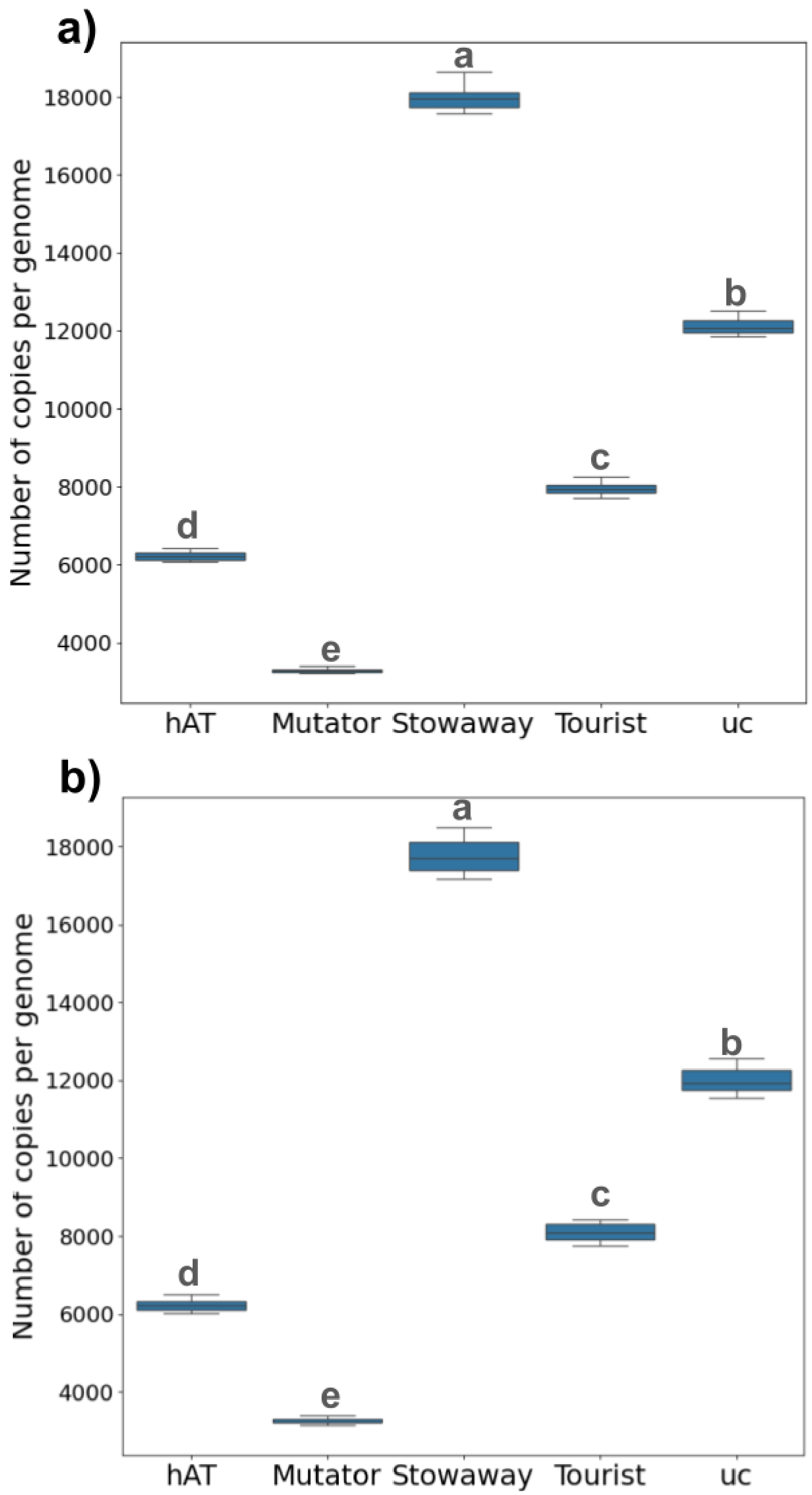

Fig. S4. Box plots showing the number of MITE copies of each superfamily considering the variability among the 12 genomes in the P1 (a) and P4 (b) F2 sugar beet families. Letters reflect the significance of differences according to one-way ANOVA and Tukey's HSD test as post-hoc at the p-value=0.05.

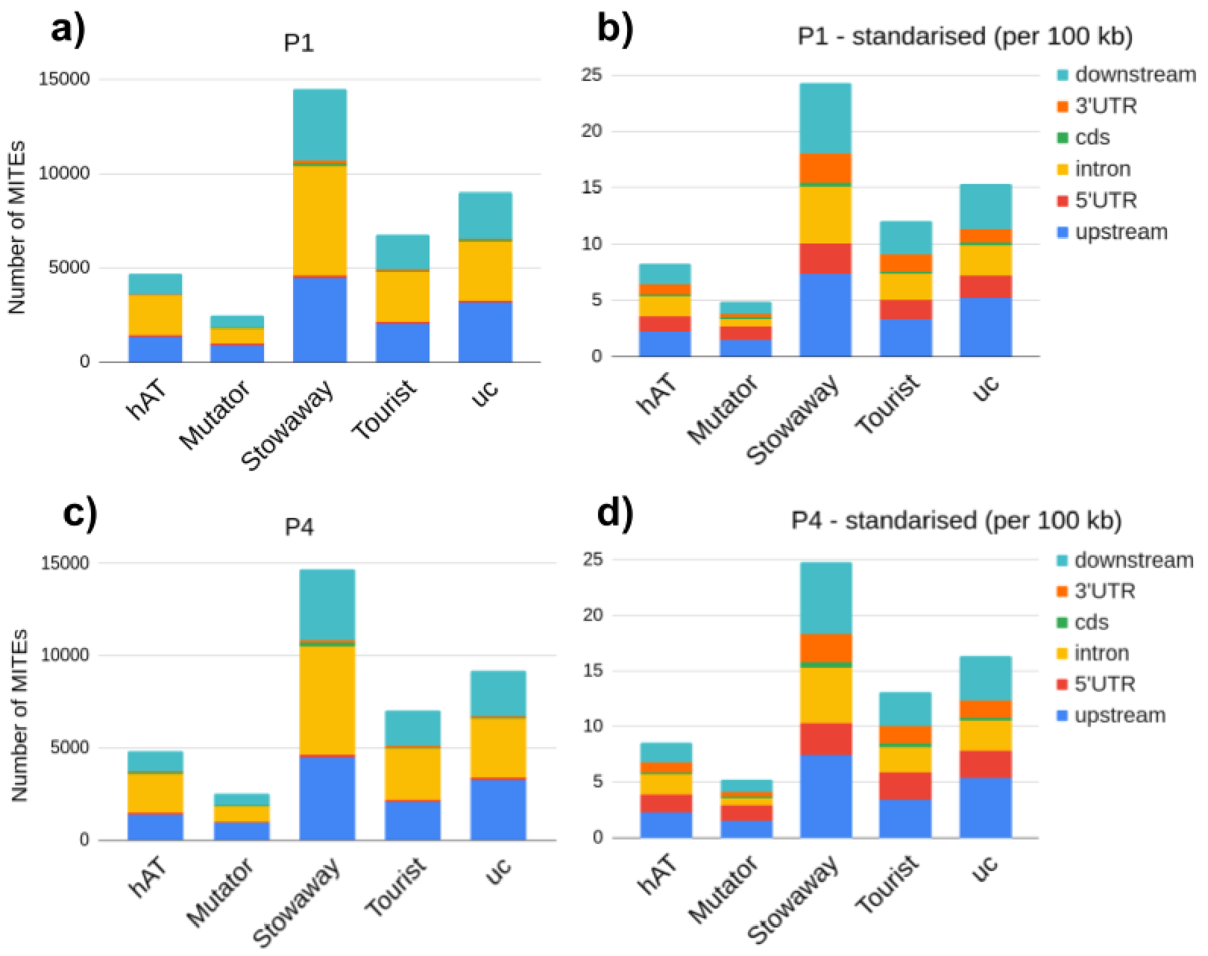

Fig. S5. The number of MITE copies within genic regions in the sugar beet genome. The number of insertions in up- and downstream regions, CDS, introns and UTRs in two sugar beet F2 families, P1 (a) and P4 (b). The number of insertions per 100 kb (standardised to the cumulative length of each region) in P1 (c) and P4 (d). 'uc' stands for unclassified MITEs.

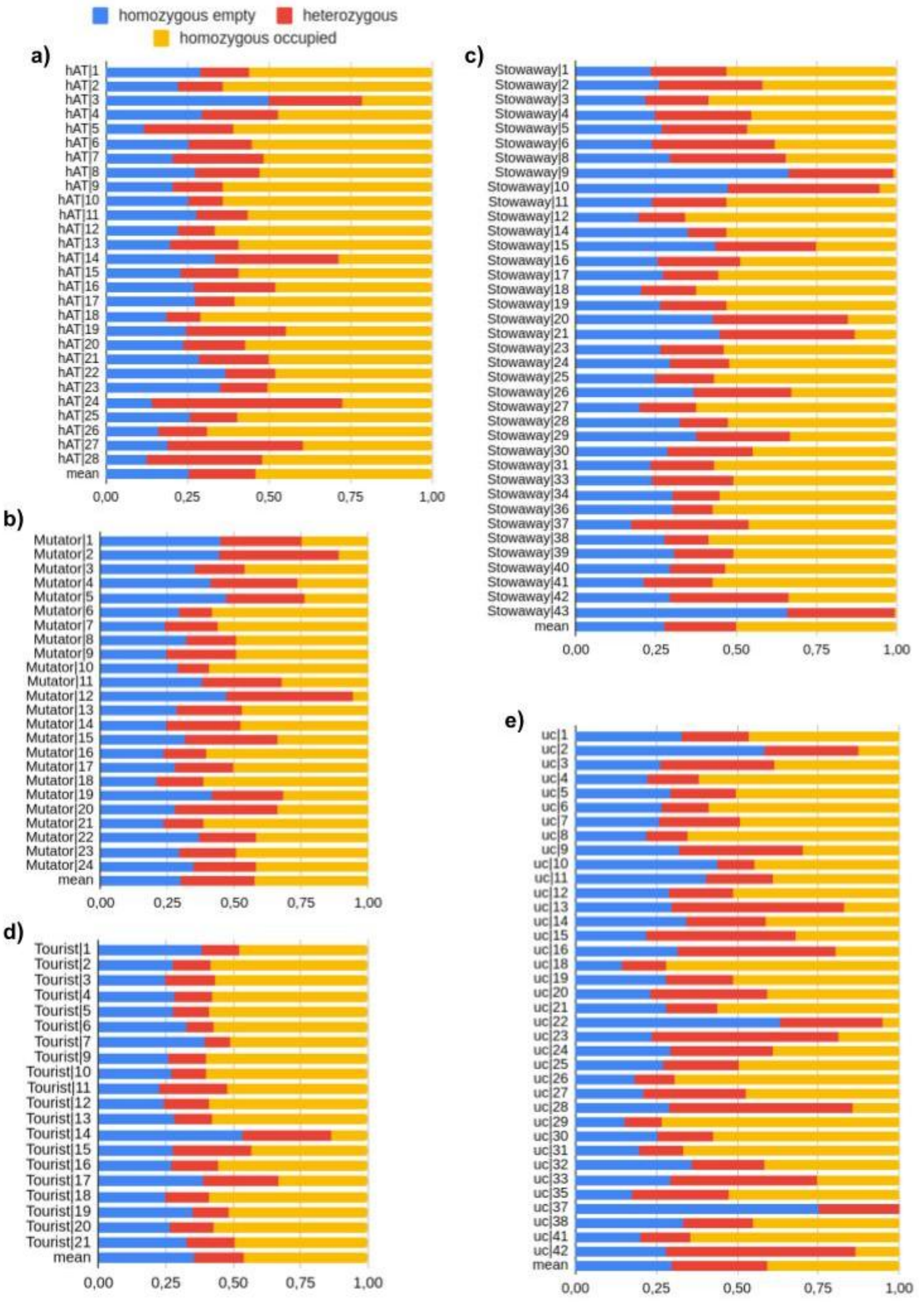

Fig. S6. Variant frequency observed at MITE insertion sites in the P1 sugar beet F2 family for a) hAT, b) Mutator, c) Stowaway, d) Tourist and e) uc (unclassified) groups

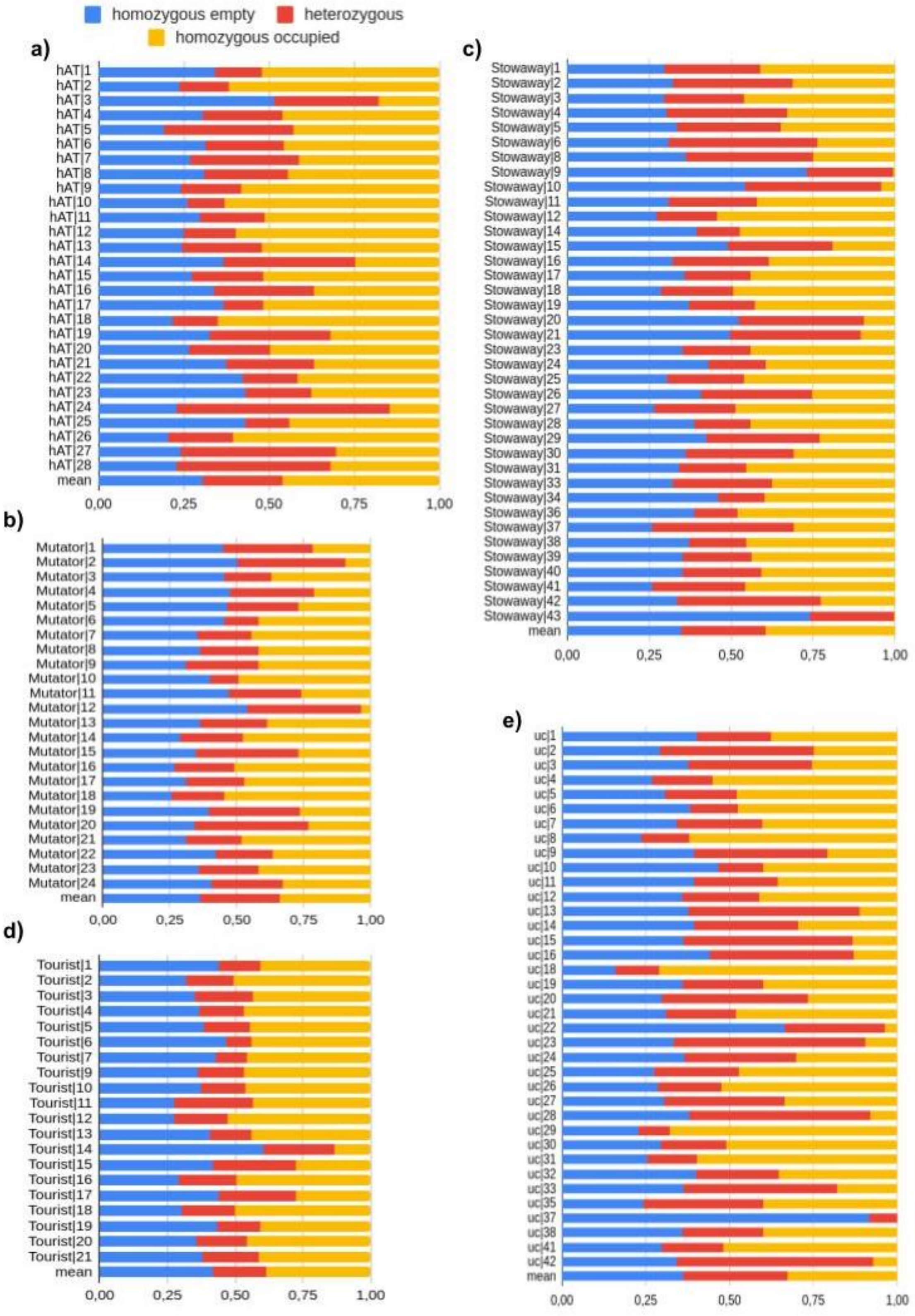

Fig. S7. Variant frequency observed at MITE insertion sites in the P4 F2 sugar beet family for a) hAT, b) Mutator, c) Stowaway, d) Tourist and e) uc (unclassified) groups

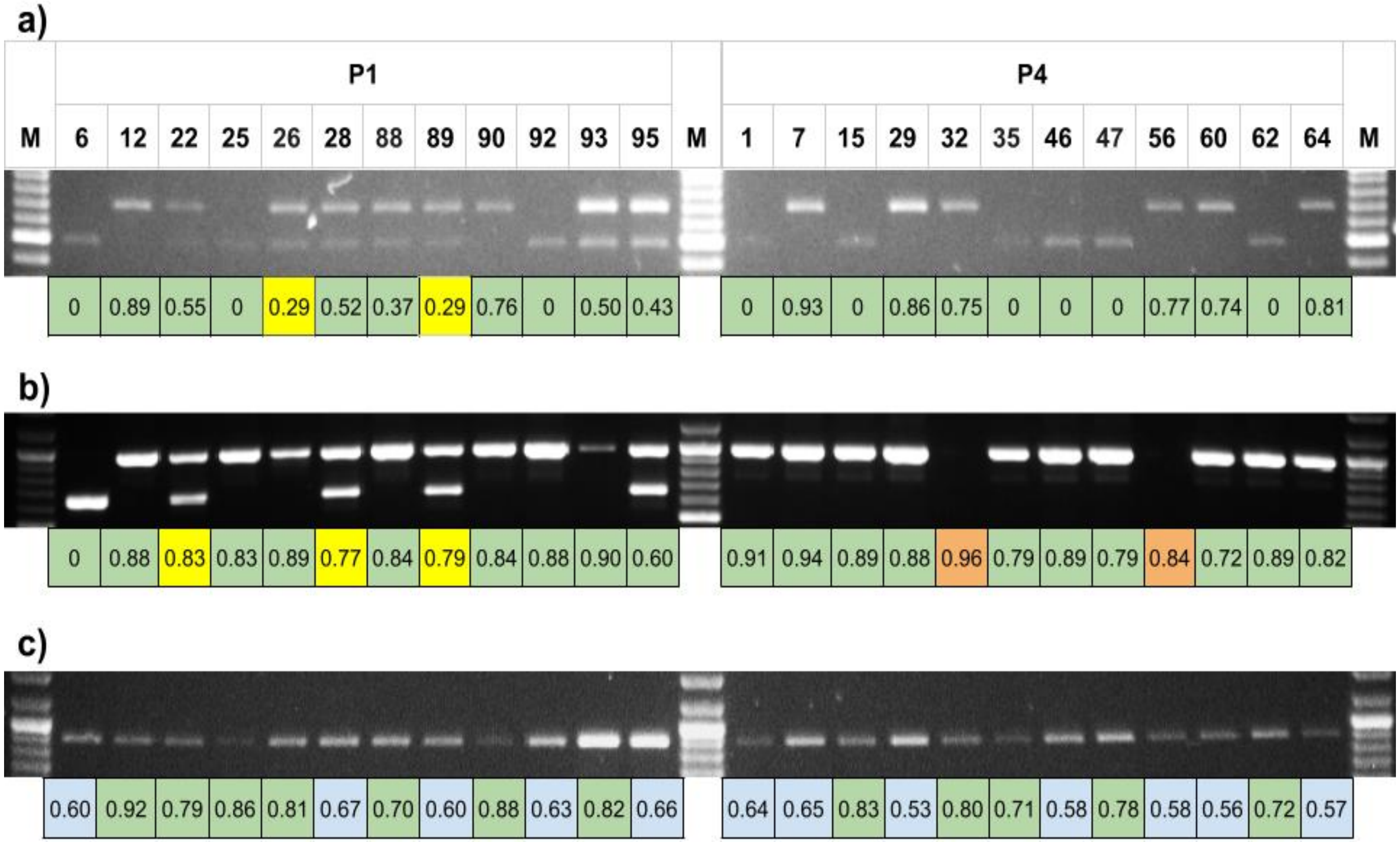

Fig. S8. Comparison of results of *in silico* MITE insertion sites prediction by PopoolationTE2 with the experimental verification in the P1 and P4 F2 sugar beet families, using primer pairs: Bv_chr3_hAT_27_20 (a), Bv_chr4_Sto36_1_8 (b) and Bv_chr3_hAT28_1_1 (c). Numbers above the electrophoregrams represent consecutive plants within the P1 and P4 F2 sugar beet families; M - 100 bp+ DNA Ladder. The values below the images refer to the frequency for each MITE insertion, as calculated by PopoolationTE2. Colours indicate, respectively: green - consistency of *in silico* prediction and PCR verification; yellow - homozygote by PopoolationTE2, heterozygote by PCR; blue - heterozygote by PopoolationTE2; homozygote by PCR; orange - no amplification.

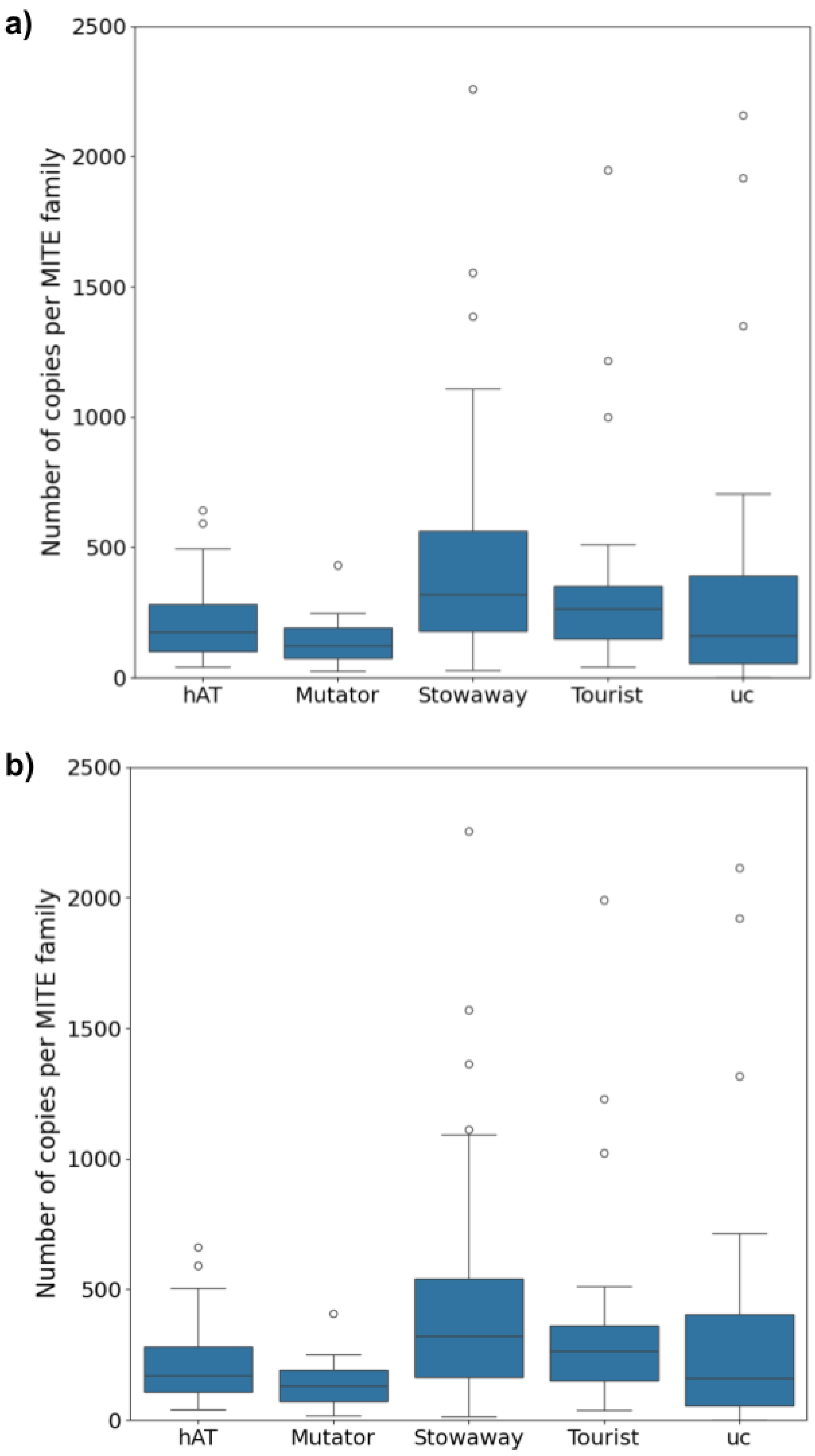

Fig. S9. Box plots showing the number of MITE copies of each superfamily considering the variability among particular MITE families for the a) P1 and b) P4 F2 sugar beet families. ‘uc’ stands for unclassified MITEs

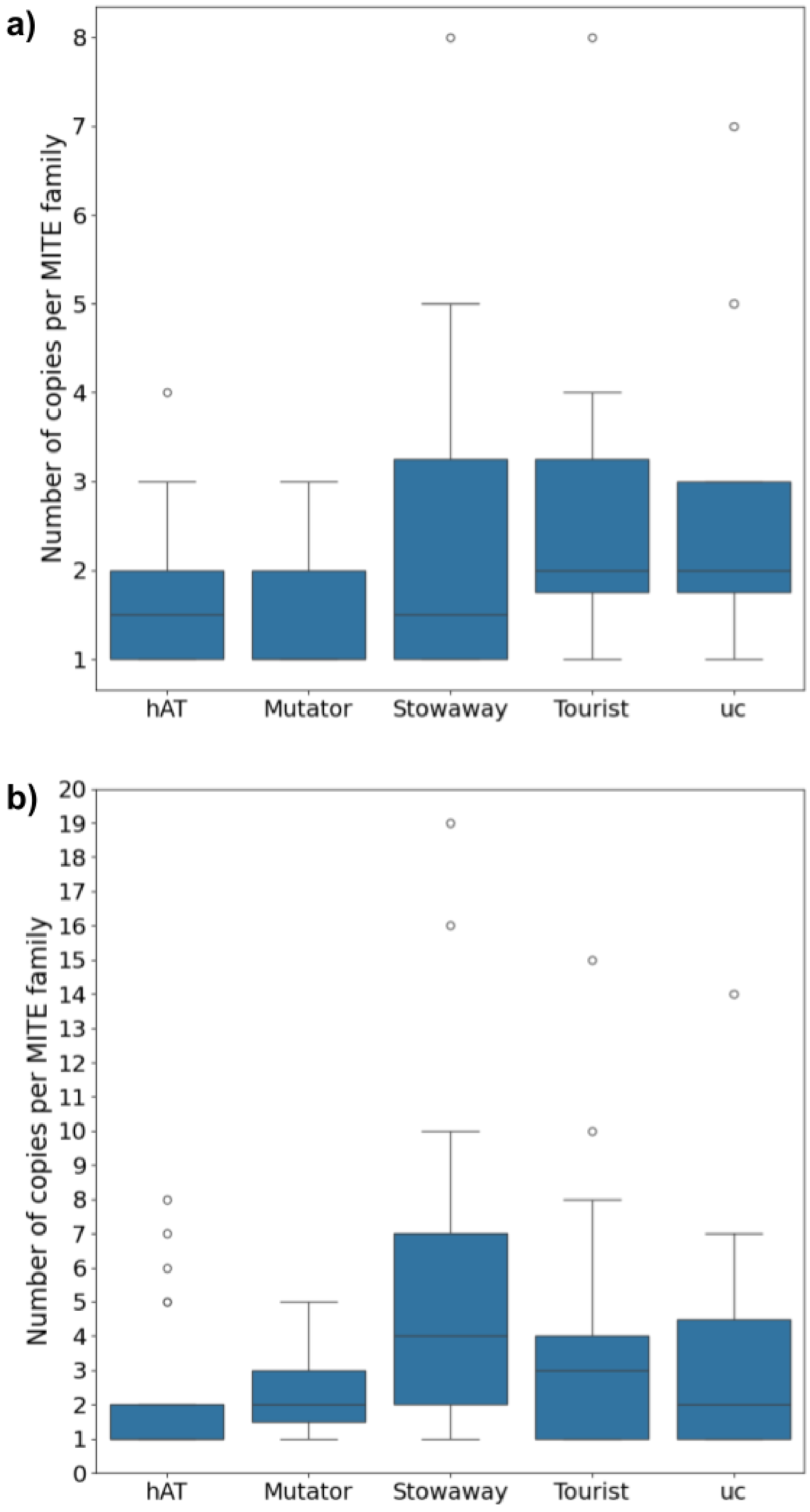

Fig. S10. Box plots showing the number of copies of each MITE superfamily, associated with DEGs, considering the variability among particular MITE families for the a) P1 and b) P4 F2 sugar beet families. 'uc' stands for unclassified MITEs.
