# Supplementary material for: Miniature inverted repeat transposable elements in the genome of sugar beet and their impact on gene expression
Source: Sci Rep. 2025 Dec 19;16:1757. doi: 10.1038/s41598-025-32772-7 (PMC12804786; doi:10.1038/s41598-025-32772-7)
Supplement: Supplementary file 3 — Supplementary Material 3 [file 41598_2025_32772_MOESM3_ESM.docx]

>Stowaway|1.1

CTTCCTCCGTTTAGAAATAGTTGCACCATTTGTATAGTGTTTACTATTCACATCTTGCAC

TTTGATTGTCTTTGGTGATTTATATTTTAGGAAAAACATAGTCATATGAGATCTTGTTAG

ATTCGTCTCAATGTATATTATTTTAATATCAACTTTTTATAATTTTTACTTATTGATAAT

TAGAGATATTAATGTTTGAAATAATGCATTGGCAAACGTGAAAACATGAAATGGTGCAAC

TATTTCTAAACGGAGGAAG

>Stowaway|2.1

CTCCCTCCGTCCTCTATTAGTTTACCCCTTTCTTTTGCACAGAGTTTTAGGAGGAATAGT

ATTGTGGGGCATACAAAGAGAGAGAAAAGATTATTTATACTTAAAGTTGAATATATGTGT

GATGAAAAGTTTGTGGTCCCATTCCAAAAATAGCAAAAAAAAGAAAGGGGTAAACTAATA

AGGGACACCCTTAAAAGGAATAGGGGTAAACTAATAGAGGACGGAGGGAG

>Stowaway|3.1

CTCCCTCCGTTTCATATTAATCTACACACTTTGACATTTTACACTATTCACAAATTGCAC

TTTGACCCTCGTTTGTGATTTATACGTGAATAAAAACATAGTCATGTGGGATCTTGTTAG

ATTCGTCTTAGTATGTATTTTATTAATATGTACTTTTTATAATTTTTACAAATACAAAAC

TAAAGATATATGTCCTCAAAGTTTTACATTGGCATGCGTGAAAAGTGTAAGTGTGTAGAT

TAAAATGAAACGGAGGGAG

>Stowaway|4.1

CTCCCTCCGTCCCAAAATATAGTTCCCATTTCTATTTTGGGAGTCAAATTTTAAAAACTT

TGACCAAATATTCTCACTAATGTATACAAAAAAACATATTCATGAGGGGTCTTGTTAGAT

TCGTCTCAATGTGTAAATTCATAATATCAACTTTTTATATTTTTTACTAATAAGAAATAG

AAGATATCATAGGTCAAAGGTACGTCTTGGAGACTGTGCAAAAAGAAATGGGAACTATAT

TTTGGGACGGAGGGAG

>Stowaway|5.1

CTCCCTCCGTCCCTTAATAGAGTGCCCATTTCTTTTTTGGACACTATTCAAAGGTAAAGA

AAGCCCTCAAAAAATATTTCTAATATATAAGAAAAAACATAGTCATGTGGGGTCTTGTTT

GATTCGTCTCATTACGTACATTAAGAAAATCAAACTTTTATAATTTTTACTAATATGAAA

TTAAAGATATTAACGATACAAAACGTGCATTGGCAAACGTGCATTGGAGAAATGGGCACT

CTATTAAGGGACGGAGGGAG

>Stowaway|6.1

CTTCCTCCGTTTCAATTTAGTTGTGACACTTTTAAAATCACGGACTCCTAGGCAATTTTT

GGAGAGGAGAGAGATAGAGAAAGAAAATAAAAGGGTCCCATGTGATAGGAGAGAGATAGA

GAGAAGTTATTACCCAAAATGAAAGTGTCACAACTAAATTGAAACTTCCTAAAATAGAAA

GTGTCACAACTAAATTGAAACGGAGGAAG

>Stowaway|8.1

CTTCCTCCGTTTCAAAATAGTTGCACCGTTTGCTTTTTCACGTTTGCCAAGACGCTTTTT

TGAACGTTAATATCTCTAATTGCGTATAAGTAAAAATTATAAAAAGTTGATATTTGAAAT

CCTCGTATTGAGACGAATTTAACAAGATCCCACTTGACTATGTTTTTTCTTATATAATGG

TGAGAGAAAGCTTGTCAAAGTTGGTTCATGAATAGTGACCAAATGCCAAATGGTGCAACT

ATTTTGAAACGGAGGAAG

>Stowaway|9.1

CTACCTCTGTTTTTTTTAGTTGCAACGATTAGACATAAAGTGTGAGTAGAAAATGTCTAA

TCGTTGCAACTAAAAAAAACAGAGGTAG

>Stowaway|10.1

CTTCCTCCGTTCTGAAATAAGTGAAACACTTCTCTCAAAAAGCTCTCACATAAAAGAGAG

AAGTGTTTCACTTATTTCAGAACGGAGGAAG

>Stowaway|11.1

CTTCCTCCGTTTCTTAATAAGTGCAACATAGAGAAAGTGGGGGAATACCAAGGAATGTAG

AGAGAGAAAGTTGGGACCACAATAAAGTAGAGTAATTCAAGTGATGAAACAAAAAAGTTG

TGGTCCCCTCTTGCTTTTTGGTGGCATATTTGTAATAATATTGAACTTTTTTTACCATTT

TGAGCTTGGTTGCACTTATTGTGACTATTCCAAATAAGGGAAATGTTGCACTTATTAAGA

AACGGAGGTAG

>Stowaway|12.1

CTCCCTCCGTCCCAATTTAGTTGCTACATTTGCTTTTTCACAGTTTACGAGATGCAACTT

CAAACACCAATATCTTGAGTTACATATATGAAAAAATTATAAAAATTATATATTTGGAAA

ATACAAATTGGGACGAATCAAACAAGATCCAACATGACTATGTTTTTTCTTATGTATATG

GAGTAAAAGATAGTCAAAGTTATGACGTGAATAGTGCCTACGGTCAAAATGTAGCAACTA

AATTGGGACGGAGGGAG

>Stowaway|14.1

CTCCCTCCGTCCCAAAATATAGTTCCCATTTCCATTTTGGGTGTCCCAAAATATAGTTCC

CATTTCCATATTTAGTTCCACATTTTTCCGTAATTTGTCTAGAAAAATCGTGTCCCATCA

TTTATTTGCTTCTTGAATTTTGTTTTTTCCTTTGTTTATTCAACCAAAATGTTACAATTA

ATGCTCTTTCACCAATTATCTCCATTCTCTCTCCTAAAATCATCTTTTCCCATACAACAT

TTATTTAAATAAACAAAAAATCTTTACTATCCCAACTTGCATAAATTACCGTGAAAAAAG

GGAAATGGGAACTATATTTTGGGACGGAGGGAG

>Stowaway|15.1

CTTCCTCTTTTTTTTTTACTTGCAACACTTTCCTAAAAGGGAAATAATTAATTACTTGCA

ACACTTTCTACTTTTGCTTTTCCTTTCTCTCTATCTCTCTCCAATCACATGGGGCCCTCT

CTTTTTTTCCTCTCTACCTCTCTCCTACCCAAATTTTGCATTGGAGTTGATGAAAAAAAA

AGTGTTGCAAGTAAAAAAAAACGGAGGAAG

>Stowaway|16.1

CTCCCTCCGTTTCAAATTATAAGGCATGTAAACCACTTTTACGCATATTAAGAATTTAGT

TAGTTTTAGTTAGATTTGCATAGGTAATAAGAATTGACATTAAATATTGTGTTGATAATA

GAAAATAACCTATAATCTTATCTTGATAGTATAAAATTGCATTTAATTTTACATTGGTAA

TGTAAAACGCCTTATATTGTGAAACAAAATTTTTTAGCTTAAATGCCTTATAATTTGAAA

CAGAGGGAG

>Stowaway|17.1

CTTCCTCCGTTTTTTTATAGATGCTACATTTCCCTTAAATGGAAATGTCATAATAGATGC

TACATACCATAAATGGATTCCTTTTCACCATAAAAATCTTAAAATACTATTAAAAAGTTG

GTGGTCCCCCACCTTCTCTTTTAAAATACTCATACTTTTTCCTTAACAAATGTTTTTTTA

TTCAAAAAGTGGTGGTCCCCTACTTTCTCTCTCTACCTTTCTTAAAAACCCCCCACTTTC

TCTATGTAGCAAGAATAAAAACACGGAGGAAG

>Stowaway|18.1

CTTCCTCCGTTCCGTTTTAAATGAAACGTTTGCTTTTTCACGCAACCCAACGCGCTTCTT

TAGTCATTAATATCTTTAGTTGTGTACACGTAAAAAATATAAAAAAGTTATAATTTGATA

GTATGCATCGAGACGAATCTAACAAGATCCCACTTGACTATGTTTTTCTTTATATAATAG

CGATAAATTGGAGTTAAAATTGATCAATGAATAGTGTCATTTCATCCAATGTTTCATTTA

AAACAGAACGGAGGAAG

>Stowaway|19.1

CTCCCTCCGTTTTTTTTAGATGTAACATTGGACTTTTCACATTTGCCGAGGCACAACTTT

GACCGATAATATCTCCAATTGTATAAAAGTAAAAATTATAAAAAATATATATTTGGAAAG

TGTATACCGTGACGAATCAAACAAGATCCCACATGACTATATTTTTTCTTATGTATAAAT

AACAATTAGTGGTCAAAGTGTATTGGATGAATAGTGCCCAAAGTCAATATGTTACATCTA

AAAAAAACGGAGGGAG

>Stowaway|21.1

CTTCCTCCGTTTCATAATAGATGCCACATTGTACATTTTTGGGTGTTTCAAAATAGATGC

AACAATGTTTTTTTTCCTTATTTAGTAACTTTTGCCTTATACTTTTACCATTTTACCCTT

ACTAACTTGTTTTAAGTACACTAATCTACTTCACTTTATATGTGGTCCCCAACAGTTTTT

TTTTCCCTCCTTTCACATGGGGACCACTACAATTAATCTACTTCACTTTATACTACTTAA

ACATGCTTAGGAAATTTTGCCTAGGAGTCCGTTTTAACAACAATGTGGCATCTATTATGA

AACGGAGGAAG

>Stowaway|23.1

CTCCCTCCGCTTTTATTTATTTGCTACATTTTGGTTTACACAAGAATTTAGGAGATGTCA

TTTTCATTCTTTTTTAGTGTACTTTTTGACCCCTTTAGCATTAAATAGGGTGGGAAAAGG

GAAAAAAGTAGTACTTTTTGGTGGAAAAAGAGGGGAAACAAATATAATACTATTACTTTT

TGGTGGGCCAAGTCTCCTAATAAGGAAATGTAGCAAATAAAAAAAAGCAACCCTTTTTGG

AAAATGTAGCAAATAAATAAAAGCAGAGGGAG

>Stowaway|24.1

CTCCCTCCGTCCCATAATATAAGTCCTGATTCTGTTTTTACGCGGGAATTAAGGTTAAAT

GGAATCTTGTTTTATTTGAATAAAAAAAATTATTTTTATTGAGAAACACTTACATTGTTT

TTTTCTTTTTAACAAAAAATAAAAACTGTCATTTAATGTTGCATGGTTTAATGTGTAGAG

GAGTTCAATGGGGTAAAAGATTCCATTTTGTCATTAATAATGGTGCATTGGAAGCTGAAA

TTATGATGACATTTTGGGAATTAAACTATTTTCATGAGGGTATTTTGGGAATAATTATTG

ATTGAAAATAGAAATAAGACTTATATTTTGGGACACCTTATAAGGAAACAGGACTTATAT

TATGGGACGGAGGGAG

>Stowaway|25.1

CTTCCTCCGTTCCATAATAAGTGAAACACTTACTATTTTAGAACGTTTCAAAATAAGTGA

AACATACCTTAAATGGAAAAAAGAAAGTGACAAAATGACAAGAGTACCCATCACAAACTT

GTGTGGTTTGCTTTTCTCTCTCCTCCTTTTGATAATAAAAAGGGTATTTTATGACTTTTT

GGCTTTTGAAAGTGATGTTTATTAAATCTTGTGCCCAATCCCTATGTTTCACTTATTATG

GAACGGAGGAAG

>Stowaway|26.1

CTCCCTCCGTTTTTTTTTATTTGCTACATTGTTGCTTTGGGAATGAAGCCACTAACTTTG

ACCGATTATTTTGATTATTTTATAAGTGAAAATATAGTCATGTGTGGTCTTGTTAGATTC

GTCTCGACGTGTAGATTCATAATATCAAATTTTTATAATTTTTACTTAAACGAAATAAGA

GATATTAAGGGTTAAATAATGCCTTAAAAACCGTGCACAACAATGTAGCAAATAAAAAAA

AGCAGAGGTAG

>Stowaway|27.1

CTTCCTCCGTTCCGAATTAAATGCAACAATTCCACTTTTTCACTATTCACACTTTTCACT

TTGACTAAGATTTGTGATTTATACATAATGAAAAACATAGTCAAGTGGGATCTTGTTAGA

TTCGTCTCGTGACGTACTTTCATAAAATGTAATTTTTATAATTTTTACTTATATGTAATT

ATAGATATTTGTGGTCAAAATAATGTCTCGACATGCGTAAAAGTGGATTTGTTGCATTTA

ATTCGGAACGGAGGAAG

>Stowaway|28.1

CTCCCTCCGTCCCAGAATACTCTTTACACTTTCCTTTTTTATCTGTCCCAGAATACTCTT

TACACTTCTATTTTGGGATATGACCCACTATTATTTTAATCATTCTACCTTTTAACTATT

GGCCCACACTCTCTCTCCTCCAAATAAAATAATACCACTCTTATTACCTACCAACTCTAA

CCATGGCCCACATTCTCTCTCCTTAAAATAAAATAATACTACTTTTACTAACTACTAACT

CCTATCACATCTACTTTTCAATAAAATAACAATTGATAACCAAACAACAACTTATCATCT

AAAACTACGTGCCTTGATAAGTGTCAAGAGTATTCTGGGACGGAGGGAG

>Stowaway|29.1

CTTCCTCCGTTTCAAAATAGATGGTACATTGTGATTAAAACGGACTCCTAGGCAAAATTT

CATAAGTATGTTTAAGTAGTATAAAGGGAGGTAGATTAATTCTAGTGGTCCCATGTGAAA

AGGAGGGAGAAAGAAAAAGTTGGGGACCACATGTATTAATTAAGTAAAATGGTAAAAGTG

TAAGGCAAAAGTTACTCAATAAGGAAAAAAAAAACAATGTATCATCTATTTTGAAACACC

CATAAATAGACAATGTATCATCTATTTTGAAACGGAGGAAG

>Stowaway|30.1

CTTCCTCCGTTCTTTTTCTATTGCAACAATGAGATTTTTACACTATTCACTCACCACCCT

TTAACTATCTATTGTGATTTTTATGTAAGGAAAAATATAGTCATGTGGGATCTTGTTAGA

TTCGTCTCGATATATACTTTCAAAATATCAAATTTGTATAATTTTTACTAAAAAGTAATT

AAAGATATTAATAGTTAAAGTTGTGCATTGGCAAGCGTGAAAACTCAATTGTTGCAATAG

AAAAAGAACGGAGGAAG

>Stowaway|31.1

CTCCCTCCGTCCCTAAATAGAGTGCCCATTTCTTTTGGGCACAAGTATTAAGAAGAAATC

ATGTGTAGGACCTTAGTTGTATTAAATCATAATTAAATATAGAAGTTGGTACACTTTTTA

TGAATTGTTTATTAAGGAGAGAGAATGTGGGCCATATGGGAAAGTGGGTGATATTATAAA

AATTGAAAGCAAAATAATATTTTAATTGGTGGACCCATACCAAATAAGGAAATGGGAAAT

CTATTTGGGACAAACATAAATGGAAAAATGGGCACTCTATTTAGGGACGGAGGGAG

>Stowaway|33.1

CTCCCTCCGTCCTCTATTAGTTTACCCCTTTCTTTGGGGCACATTTGCCGAGATACTAAT

TTGAACCTTAATATCTCTAATTTTCGATAAGCAAAAATTACAAAAAGTTGATATTTGGAT

AATACACATTGAGACGAATCCAACAAGATCTCACATGACTATATTTTTCCTTAAATACAA

ATTACAAAAGATAGTCAAAGTCATGTATGTGAATAGTGTCAAAAGTCAAAAGGGGTAAAC

TAATAAAAGACGGAGGGAG

>Stowaway|34.1

CTCCCTCCGTCCCATAATATAGTGCTCACTTCTCATTTTTCACAAGAATTAAGGAAAATG

GAAAGAGTTTTAGGATTTCACAAAAATATATATTTAAGTAAATGTTTATTGGGTATTTTT

GTCTTGGAACTAGTTTTTTTCTTCTTTTTCCTTTAAAATAAAATGAAAGAGCAATAAATA

TACCTTGGAATGGTGTAAAACAATAAATAGGGGCACATATTTTAAAAGAAAGTAGGGGTA

TTTTGGAAAGTTTTATGTAGAAATGAAGGATAATTTAGTCCAAACAAATTGATAAAAAAG

AAATAGGCACAACATTTTGGGACACCCAAAGAGGAATGCAAGCACTATATTATGGGACGG

AGGGAG

>Stowaway|36.1

CTCCCTCCGTTTCCTAAAGATCTTCCCTCTTTCCGTTTAAGTGCGTTTCCAAAAGATCTT

CCCTCTTTTACTTTATTCCTTATTTGGAATTTTTTTTCCCTTTTTACCCTCTCATGTCCC

TATCATATGGTCCACCATTACATTATTAATTCAATTTTACTCCCTTTCTCTCTCCTACTT

TAACCCCTTTTTATTTGTTTATTTATCTTAACCCCTTTCTCTCTCCTACTTTACCCACCC

CACACCCCACACAACTTGATTCTCATTGGTATCTGTAGTAACAGTAACAAGGAAGATCTT

TAGGAAACGGAGGTAG

>Stowaway|37.1

CTTCCTCCGTAACAAAATAGATGTTACACTTTCTAAATATGGTTGTAACAAAATAGATGT

TACACTTCCTTTTTAGGCAACAATCTCTCTCTATCTCTCTCCTATCACATGGGTCTCCTT

TTTTCTTTCTCTCTATCTCTCTCCTCTTCAAAAAATTGCCTAGAAAAGTGTGTTTTTAAA

AGTGTAACATCTATTTTGTTACGGAGGAAG

>Stowaway|38.1

CTACCTCTGTTTTTATTTAGTTGCAACAATTTCTACTTTTAGGTGTTTTTTATTAGTTGC

AACATTTCCCTTTTTAGGAAAGCTTTTGGGATAATTTGACTATATTACCCTTACTTCACC

TACTCTCCCCATTTAATTACAGTACTAGTAGCAGGGGCAAGTGGGTCAATTATACTCTCT

TTTCTCATTTCTTAAAGTTTGTGCAAAAAGGTGAATGTTGCAACTAAATAAAAACAGAGG

TAG

>Stowaway|39.1

CTCCCTCCGTCTTTTTTTATTTGCTACATTTTGCCATTTAGGGGTGTTTCAATTTATTTG

CTACATTTGCAAATATTTCCATATATAGTACATAGGTCCCACACTTTATTTACAAATTTG

CCATCACTCCCTTCTTGATTTTGTAAATGTTTCCATATATAATACATAGGTCCCACAATT

TACCTATTTGCTTTTTTGTTAATTCCATTTATGTCAATGGTCCCCACTCCCTCCTTGGTC

TTTGTGAAAAAGTGCTTGTAGCAAATAAAAAAAGACGGAGGGAG

>Stowaway|40.1

CTACCTCTGTTTTTATTTAGTTGCAACGTTTTGACTTTCACGCTTGCCAATGTACAATTT

CAAATCGTTTTATCTCTAATTGTATGTAAGTAAAAATTATAAAAATTTGATATTATGAAA

GCTTACATCGAAACGAATCAAACAAGACCCCACATGACTATATTTTGTTTAACATATAAA

TCAATAAATGTGGTCAAATAAGAGAACATGAATAGTGCAATTTGTCAAACGTTGCAACTA

AAAAAGAACGGAGGTAG

>Stowaway|41.1

CTCCCTCCGTTTCACAATATAAGGCACGCACGATGTTCGAGGTCGAACTTTGACCGTTAA

TTACTCTCAATTTATGTTGATATAAAATTTTAAAAAATAATATTATTATAGTATTTTTCA

AGACGAATCCAACGGTATAATTTTTATAATTTTTACTTATCGTATTTGAGAGATATTTGT

GGTCAAAGCAAATTTTCGTAAAGCGTAAATGCCTTATATTTTGAAACAGAGGGAG

>Stowaway|42.1

CTTCCTCCGTTTCTAAATAAATGCAACATTTTGACTTTCACGCTTGTCAATGCACAACTT

TGACTATTAATATCTTTAATTATGTATTAGTAAAAATTATAAAAAGTTGATATTATGAAA

ATATACATTAAAACAAATCCAACAACATATTATATTATAACATTTGTTTTTATATATTAG

TAAATAAAGCATGGTCAAAGTTCATTTATGAATAGTGCAAAAGTCAAAATGTTGCATTTA

AAAAGAAACGGAGGAAG

>Stowaway|43.1

CTTCCTCCGTTTCGTTTTAAATGCAACAAATGAATATTTTATATCTCACAAATAATACCC

CTTTGTTGCATTTAAAACGAAACGGAGGAAG

>uc|1.1

TATATATAGAGGCAAGATCACATGAGAATGCTCCTTACATGAGAAATGAGAATGAATCTC

AACCACACGATTGGTTTAGAGTTACAACACTTATTCATTAGAGTTATATCCCTCTAAAAA

AATTTGAATTCAAAAAAAAATTTGTTTTTTTTGAATTTTTTTTTCAAATATTTTGTTTTT

GGAGTTACATCACTTTTTCATTAGAGTTACATCACTTATTCATAAGAGTTATAGCGTGTA

AAATTCATTCTCATTTCTCATATAAGTATGCATTCTCATATGAACTCACTTCTATATATA

TATA

>uc|3.1

TGAACTGATATTTTAGGGGAAGGGTAATTTAGGTATTATCTTAATGAGTAGCTTGGGGAA

TAAGCTAGGTAGTTGTGCTAAGGAAATTACTCTATATATAGGGATAATTAGAGAGAGAAA

GGTATGTTGAAAATTGTTAAGAAAAATTGGGTGAGCTTTTAAAGTTCAATGGGAGAGAAC

AAGCCTCTCGAAAGCTTGTAACTATCATTTTATATTGCTGTAATTTCTGTTTTTCAATCA

AATACTCATTTTCTTCCTTAATTTCTGCCATTAAAGTGTCCAAGCTATGTTAGTTCA

>uc|4.1

GGGGTGTTCATGGGTGGTTAACCGTCCCGAAACAATATTTCGGTTCGGTAACCGGAAGTT

TCGGTACGCTCTTTTTGCGAACCGTAACCGTAACCGTTTAAACCGGTTACCGCCTAAGCG

GTTAACCGTTTTTTCGGTACGGTTTTTCGGTTAACCATTAACCGTTGTTTAAGTTATACT

ATTATGTTTTACTTAATGTACATATATATTTTCAATTTTTTTGTGCATATATTTTAATAT

TATATATACTCTAATATAAAATGTATTATAAAGTGGATAATAAATCAATTGGAAAACATA

ACTAACACTAAGAATTCACTTCATTTCCTAATTACAATGTTTATATTAGTACTAGAGTTT

CTAAATAATTACATACTTCCTCTAATTGTTGTAACTTAGCAACAAATATTAGAATTAATG

TAATTGTTTGAAAATGTTTCACAACAATTAAATAGAGAAAAAAAGATTTCGGTATTTCGG

TTAACCGTTTACTCAAAAATCATAACCGTAACCGTACCGTTTAACCGCTAATTTTTTCGG

TTCGGTAACCGTACCGTTCAAACTTTTTTTTGTCGGTACGGTAACCACCCATTTAGACCG

GTTTTTCGGTATTTCGGTACGGTATTTCGGTTTCGGTTTTTTATGAACACCCC

>uc|5.1

GAGTTATGGTAAGTATATTATATTAGAGTCCTAGACTAATAAGGAAACGAGTATAGTTTT

TATATAAACTATACTCGTTTCCTTATTAGTCTAGGACTCTAATATAATATACTTACCATA

ACTC

>uc|6.1

GGGGTGTAAATGATGCGAGTCGAGTTCGAGTTTGAGCTAGCTCGACTCGACTCGTTAGCA

TTTCGAGTCGAGCTTGATTGACTCGAGTCGAGTTTTGACCAGTCGAGCTTTGACCGAGTA

GATTTCGAGTAGCTTGCAAGTGTCTCATGTTAAAAGCAACAATAAGGTAGAAAAATGTTG

AATTTAGGTATTTTGCTATTGATAATTGGGTATTCGAGCTTCTCACGAGTTTTCGAGTCG

AGTATCGAGTTGCTCAACTCGACTCACCAACATCTCGAGTCGAGCTCGAGTTCGATTCGA

GCTCACTCGAGTCGAGCTTTGACCGAGTCGATCTCGAGTAGCTCGCGAGTTGTCTCGTCT

CATTTACACCCC

>uc|7.1

GAGCTGTCAAAAAATGACCTGATCTGATAACCCGACCTGAACCTGTAATGACCTGTAACT

GACTTGCACCTGAAATTTTGGCAAAACAGGTCACCTGAAACCTGACCTGTACCCGACCTG

ATAATAACTGATACCTGCTCTTGACCCGATACTCTATGACCTGAACCTGAACCGATACCC

GCCCAAAAAAGACCGATAACTGAAATGACCCGACCTGACAACGATATGAACCCGATTCGA

CACCTGACCCGATTAGGACCTGAAACTGATTATAACTCGACGTAACTTGTCATTAACCCG

ACCTGTTGCGACCCGTTACCTGATTTTACTAGACCTGTTGTGACCCGGTACATTATTTTA

CTTGACTTGGTTCTACATGTTACCCGATTTTACCCGACTTGTTGATTGTTGATAACTCGA

CTTGTCATTGATTTCACGACATAGTAACATAAATCATAAACTAACACTACTATAAAAAGG

CATATAACTAGATGAACTATTCAAGCATTGATTTTCAATGAAATCTTAAATAATGATACA

AATTTGAAGCAACGCCACACAAAATTCATGACCTGTTCCGTACTCGAAGCGACCCGAAAG

TGACCTGAAACCAAGTATGGTATAACCCGATAACTACCAACCTGAACCCGACTCGAACCT

GACCCAAACCGCTATTGTCCTCACCTGATAAGTATCCGACCCATGCATGACCTGAACCTG

AAAGCACCTGCTACAAACCTGCTATAAACCCGACCCGACACCACATAATCACGAAAGATG

ACCTGTCCCGAAATTGACCGCGCCGAACCCGACCGAACCCGAAAGAAGAAATGAACCGAG

ACAGACCTGACCGAATTATGACCGTTCCCGACCTGCACCCGACCTGTTTGATTTAATGAC

CTGACACGACCTGACCGAACATGACCTGAAACCTGAGATGACCCGACCTGAACCCGATTC

GAGTAACTGAATTGACAGCTC

>uc|8.1

GGGATGGCAATGGGTCCTGGACCCGACCCAGACCCGCTAGGACCCGACCCATTTATAAGG

GTCTGGGTCCAGAAATTTTAGACCCAATGGGTCCGGGTCGGGTCTGGGTCCCGAAGAGCA

TGGGTCGGGTCTGGGTCCGGGTCCACATATGCTAGACCCAGACCCGCCCCGCGGACCCAT

TTACATATTAGAAATTTTTTTTTTACCTAAACTCTAAAATTTTATGAGTTGTGATTTTGT

GAACTCGTTAGTGTTGTAACTCCACTAAGACATGTAACTTTATAAGTTGTAACATTTTAA

TATTTTATGAGTTAATTCTTAAAATTTTATTTAGAGAATGCTTTAACCATGGATACAAGA

ACCTATATATATTAGGGCGAAAATTTTTAATTACATATATTCAAAAATGTGATGAAAACT

TAAAACTAAGAAGGACCCATTTAGGACCCAGTAAGGACCCATGGACCCGCTAGACCCAGC

GGATCTGGGTCTGGGTCTGAAAAATCTAGACCCAACGGATCTGGGGCGGGTCTGGGTCTG

TGAAAAATAAACGGGTCTGGGTCTGGGTCCAGGTGGACCCGACCCAGACCCGCCCCATTG

CCATCCC

>Tourist|1.1

GGCTCCGTTTGGTAGGGCGTAAAACGTTTTCCCGGAAAACTGTTTTCCTCTATTTTCAGT

TTTACATTGTTTGGTTGGCAAAAGAGTGTAAAACCATTTTCCCTTGGGGTAAAATTACTC

TCCCAATGATGGAAAACCATTTTCCTTTCAAAATGAAGGGAAAACTATTTTCCTTATCTC

TCTTGTACACTCCTCTCACTACCTCCTTACTTTCCCTTTCATTTTCCTTTCATTTCATCA

TTTTTCTTACATCGAACCAAACAACGGAAAACTAATTTTGGAATTGTGTTTTCCATTGTA

AATTGTTTTCCATGAAAATCATTTTACACTGAAAATGTTTTACGCCCTACCAAACGGAGC

C

>Tourist|2.1

GAGGGTGTTTGGTATGGAAAATTTTGATGTAGGAAACGGATTCAAATAAGTGTTTCCATT

TCTAATTGTTTGGTATGAGTGTTAAGTAACAAATACCGTTTCCAAGGGGAAACATAATCC

CCCCATTTGTTACCCTTATCAAACTAGTGGAAACAAATCACGTTTCCTTCCTCAATCATA

ATCCTCTCAATTGTTTCCATTCCACTAAGATACCAAACAACAATTAAAGGTAACGGTTAT

TATTTCCATTTCCCCTCCTTATCCAAATCCTTTCCATTCCGTTTACGTTTTCCATACCAA

ACACCCTC

>Tourist|3.1

GGGGGTGTTTGGTATCATGTTTTTTCACAAAAGAAACGGAATCAAATAACAAAATCCGTT

TCTACTTGTTTGGTTAGTGTGTAAAGTAACAATTACCGTTACTTGAGGGAAACACATCCC

CCCAAGTTGTTTCCACTCAAAAACCTAAGGAAACAAATAACGTTTCACTCCTCAAACAAG

AGGAAAGGGAGTGGGTTATATTTGTTTCTATTCCATTAGGATACCAAACAACTAATAAGG

GTAACACTTATTATTACCCTTTCCCCTCCTTATCAAAATCCTTTCCATTCCGTTTACGGT

ATTCATACCAAACACCCCC

>Tourist|4.1

GGGCGTGTTCGGCACAAGCTTTTTAAGTAGCTTTTAGTGTCTTGACTAGGTCAAAACACT

AATTAGAAAAAATAAGAGTGTTTGGCAAGGTAGTGGTGTAAGTAGCTTTTAGTGGTACAA

GTAGTGTTTTAGTAGTGTTTGTCAAAAGCTACAATTTGTAGCTTTTGGGTAGCTTTTAGT

GGTGTATAAATTATATGTTAAAGTTTTATTTGAATTTTTAACTAGTTATTCTTAAACAGC

TACTTTGACAGCTTCTTTTACCGAACATCAATATTTTACACCGCTACTTGGACAGCTAAC

AGCTAACAGCTACACACCAACCACTAACAGCTACCAGCACAGCTAACAGCTACTCTCACA

GCTAAAAGCTACCACCACAGCTACTGTTGCCGAACACGCCC

>Tourist|5.1

GGGGTCGTTTGGTTCAAGATATGGAATGGAAAGGGAATGGATACAAATAGAGGAGGGGAA

TGGTAACAAAGATGGTTACCTTTATTGAGTGTTTGGTTCAAGCCAAGGAATGGAAATGAA

GGGTAACCATTAACTTCAAGTAATGGATTTTGTTACTTGAGGGGGAGGGAAGGTAACAAA

TTGGGGGAATCCATTACCATCAAGTAATGGTAAGGGATTGATGAGGGGTTGAACCAAACA

AGAAGAAAGGGAATCGGTAATTGATTCCGTTTCTTGAAACCCAAAAATAGTGAACCAAAC

GACCCC

>Tourist|6.1

GGCTCCGTTTGGTTGGATGTAAAACATTTTCAATGTAAAATGATTTTTCATGTAAAACAT

TTTACAATGGAAAATTCAAATGCAAAGCTAATTTTCCTTTGTTTGGTTCAATGTAAAAAA

AGTTATGGAAAAGAGAAAAATGGAAGGGGAAGGGAGAGGATGGATAAGAGGATAGTAAAA

GGTTGCTAAGGAAGAGGGAGGAAAATGTGTTTTCCCTTCTTTTTGAAAGGAAAATGATTT

TCACCTAAAGCTCCCTTGATGGAAATCTTGTTTTACACCCCTTATCAAACCAAACAACGG

AAAATGAAAGGAAAAGGAAAACTCATTTTCATGGAAAATATTTTACACCCTACCAAACGG

AGCC

>Tourist|7.1

CGGCCCGTTTGGTAGTCGTTAATAAATTGCGGTAATGGGAATGAATTTTAAGTGTAATTT

TATTAGGGAAGCACCCTTAAAATCTCCATGGGGAGGCATTTTTTCTCCATGGGCAAGCAA

TCTCACCCCTAAACTTGTGTTTTTGTCTCCAAGGGGAAGCAATTTTCATTACCACTCATT

ACCACCCTACAAGGTGGTATTGGATGGGAATGAAAATTCAAGAAGGAAAGTGGACACTTT

AGGATAAATTAGCATTACCATGGGAATGGACAATGATATTTTTCACAAATTTACACACAA

ATTCATTCCCATTACCACCATTTATGACCAACTACCAAACGGGCCG

>Tourist|9.1

CGGCCCGTTTGGTAAGAGGTTATAAATGGTGGGAATGGTAATGCATTATAGTGTAAATTT

GTACTAGAAATCCAATGTCATTACCAATGGTAATGCTACTTAATCACAAGATATCCATTT

TCTTCCTAAAATCTCATTACTATCCATTACCACTTGGGAAGGTGGTATTAGGTGGTAATG

AAAATTTATAAAGGAAAACCCATGTTTTGTGATAAAGTTTCATTCCCATGGTTATGGACA

TGTGAAAATCTTTACAAATTTACACTAAATTTCATTACCATTACCACTATTTATAACCAC

TAACCAAACGGGCCG

>Tourist|11.1

GGCCCTGTTTGGCACTAGCGTTTGAGGTAGCGGTTAGCGGTTGGACAAGATTGAAACGCT

ACTTAAGAAAATTATGAGTGTTTGGTAAGGTAGTGGTTGTAGTAGCGGGTAGCAGTTGGA

GTAGATTATGAGTAGCGGTTGTGGAACGTTACTACAAGTAACGTTTGAGATTTAGAGGTA

GCGGTCTAGCAAATAAACCCTATATATTAGCATAAGGTAGAATTATTAACTAATTTTTTT

TATCTTTTACAATTCCAACAGCTACTTTTGCCAAACACTTCTAATTAAACAACTAATTTA

ATCGCTGACCGCTGACAGCTACTAGTAGACAGCTAGCAGCTACCCCCAACAGCTACCGCT

ACTACAACCGCTAACAGCTACTAGAACCGCTAGTTTTGCCAAACATGCCC

>Tourist|12.1

GGCTCTGTTTGGGGAAGCATTTCAGGTGACTTAAATGATAAGGTGCTACAATTTTAAGTC

ACCTTATATGATTAGTGGTGTTTGGTGGAGGAACTTAAATAAAATTTAAGTCCAAAATTT

GACTGAAATTGAGTTGGAGTAAAATATAGCTTCTGAGATCAGGTGCTGAAATTTTTTTAT

TATTATTATTTTCTTTTTATCTTTTTAAGCCTAAAAAGATATTTTCTTTTTATGTTTTTA

AGTCTAAAAAGATATTTCAGCTACAGTCATAAATAATATTACCAAACAGGTTATGTAATC

AGCTAGCTTATCAGTTTCAGCACCTTAACAGTTTCAGCTGCTACCTAATCAGTTTCAGCC

ACCTTATCAGTTTCAGCTCCATTTCAGTCAGTTTTGCCAAACATAGCC

>Tourist|13.1

GGCTCTGTTTGGGGAAGCATTTTAGCTACCTTAAATGATAAGGTGCTAACATATTTTAGG

TGCTAAAATGATTAGTAGTGTTTGGGGAGCACCTAAAATAAAATTTCAGGTGGCTAAAAT

GACTTAAATGATAAGTTAAAAAATTTACCTTTTCACAATCAGGTGACTGAAATTTTGGAA

AATCTTTCCAAAATTTTGACAACAACTAATTTTGCTAAAATAATTTATCAACTTAATCAA

CTAATTTTGCCAAACAATTAAACTAATCAGTTACCTTATCAGTTTCAGCACCTTATCAGT

TTCAGCTTTATCTTATCAGTTTCAGTTAACTTATCAGTTTCAGTTTATTTTCAGCTAGTA

ATGCCAAACAGAGCC

>Tourist|14.1

GGGTCCGTTTGGTAGAGTGTAAAACGTTTTCCTTGAAAACTATTTTCCTATTTTACATTG

TTTGGTTTGGTGTAAAATATTAAAATGTAAAACTTGGGATGGAAAACCATTTTCCACCCC

ATTGAAAACATTTTCCCCCAAAATTGGGAGGAAAACATTTTCCACCACTCCTCTTTGTAC

ATTCCTTTTACTACCTCTTTCATTTTCCTTGTTTTCCATCATTTTTCTTACATCGAACCA

AACAACGGAAAACTAATTTTGGAATTGTGTTTTCCATTGTAAATTGTTTTCCATGAAAAT

CATTTTACACTGAAAATGTTTTACGCCCTACCAAACGGAGCC

>Tourist|15.1

GAGCACCTCCATTCATGGGGCACCCCATTTTTTCTCTCTCCTCACCTTATTTGTCCACCT

CATTTTCCACTACCTCCCCATTTTACCTTACTCAAATTTGAAACACATACCTCCACTCAT

CTACCTCCCCATTTATCTTCTCTCTCTTATTTCCTATACTCCACCACCTTTTCTCTCTCC

TACTTTCCCATACCCCACCAATATTTTATCTATTTATTATATACCCCACCACCTTTTCTT

CATTTGGGCACCTCCCCTAAAACTTGAGCATCCTCATTAAATGGGGAACCAACCCCACTC

ATGGGGAGCCCCATTTGAGGAGAGAGAGTCCATATGGGGAGGGTATTTTTTGCCATTGGA

GGTGCTC

>Tourist|16.1

GAGCATGTACAATGGTTGGTGTCTCTCCAACTAGCTCTCCACACTCTCTCTCCTATTCTC

TCTCTTCAAGCTACTCTCAAGAGCCATGTCTAAAAACACCCAACAACAATAAAACCCTTT

AAAGAGCTCTCCATTCAACTTTTCATTATTTTGTGGTCCCCATGTCAATAAATGAACCTC

ATTTCATATTATGATCAAAGTTATATAAATTAAGTTATTCATGTAAATTAAATATAATTA

CAAATTAATCTTTTTCATAATTTAAGCAAAGTAGAATTTTTAAAAAACAATATAATTATG

AGTGCTAATATTTAATTTTCATAAATTTGGCATGGAATGAAGAATTAACCTACAACATGA

AAAACTAATTTTTAAATATCCAATATTAGGCTCATTTTATAGATAAAGGAAAATTATACT

TGTTGGCATAATAATTATTACCATCTTTTTTTTAATTTTTATAGAAAAGATCATTAAATG

GTTGCATGAAGAAAAAAGAATTGAAGTAATTACAATAAAAAGTGGGTCCCACACATTGGA

GAGCCATCTCTCCATCAACCCAAATGGTATCTCCCCCAAGAGCCCTTTGGCAAAGTGTCT

CCATGGAGAGACACCCAAGACACACCCAAGAGCCCCATTGTTGGGTAAGAGGTGGCTCTC

CATTTTCTCTCTCCTTGGAGAGCTAGTTGGAGAGCTAGTTGGAGAGCCCCATTGTACATA

CTC

>Tourist|17.1

GGCTATGTTCAGTTCACCTTATTTTTGCTTATTTCAGATCTAATCAGATCAGATCAGATC

AGAAAAAATAAGTTCAGATCAGATCAGTTCAGATCATGCTTATATTTAAAAATCATTATA

TCTTTCATTACATATTAGTAAAAATTATAATAATTGAATATTAATAAAGTACTCATTGAG

ACGAATCAAACAAGACCCCACATGACTATATTTTTTCTTATATATTGAGAAAAATCTAAA

AGATAAGATCTTGTAATCAGATCAGTTCAGTCAGATCAGATCAGATCAGAAAAAATAAGT

TCAGATCAGATCAGATAAGTTCAGATCAGAAAATTTCAGGTGAACTAAACATAGCC

>Tourist|18.1

GAGCATTCCCAATGGTGATGGGTTTGATCACCAAATTTTCCTACTTACTCATGTTTTGAT

CATTTCATGATCAAATGTATCACCTCCACCCCACTCATGAACAAAAAAACCATCAAACTC

CACTTTATCTCTCCTACTTTTCACTACCCCACCTCAACTTTCTCTCTCCTATTTCTCTTT

CCTACTTCAATTAATAATATAATTTACATATTAAAAATAAAAATGAATCATCTTATGATT

ACTTAAAATATTTTCTCTTTTTAGGTGAGGAGGAGAAGGTAAAAAATTATAGAGAAAAAT

GATGTATAAATATTTTTAGAGAGAGAAAGGTGAGGAGGAGAAGTGTTTTTTTTTGTTCTG

AAATATATCAATATGGGTATCATTTTATAGAGGGGAAAAAATATGAATTTTGGTATTTTT

TTTTGAAATAGGAATTTTCTGGGAAATTCCGTGGAAATTTCCACGAAATTTCCCAAAAAA

ACACAGCAGCAGCCACGTGGCAGCTTGCCACTGGCTGGCTGTGTGTGACAATGCACTTTT

TGTCTTCAATTTGCACGGTTCACATGGTGATGAGTTTTTTCACATGGTGAACAATGACAT

GATATGTCATTTGGTGATAGGTTTTGTATCACCAATGGGATGCATGTTTTTTTTGTTCAA

CCCAAAATTTCTGATTTTGGTGATGGATTATATCACCATTGGGAGTGCTC

>Tourist|19.1

GAGCAACTCCAATGGTAGAGCAAAATGAAAATTGGCTTAGAGTGCCACATAGATTTTTCT

AGCTAACTCAACTTTAAGCCAAAACTCCCACAATGGTTAGCCAATATACTTGTTGTTGAG

TAGGTCCCACTTGAAGTAAGATAGTTTTTTTTTTAGAGAAGAATATAGAAATTGTTCACC

AAAATTCCCACATGGGCCCACAACTTTAGCTCAAGCAAATTGGCTTAGTTAAGCTACATT

GGCTAGCAAATGAAATTTCAGCTACTCACTCCAATGGTTGGGCTAATTGCTTAACTTTTT

ATTTATTTGCTAGCAAGTCCCTAATGCTCACCATTGGAGTTGCTC

>Tourist|20.1

GGGAGTGTTTGGTTCATATGATTTTGGTATGAGGTATGGGTTTAGAATGAGCAAAACCCA

TACCTAGTGTTTGTTTAATAATTTGATAGATTTAAAACCCATACCTCAAACCCATAAGGT

ATGGGGTTTAGAAACCCAAGGGGGGAGGTGGGTTTGGCTCATACCAATACCTCTAGGTAT

TAAATAGAAGAAAAAATTTCATTCCCAAATAAAACCAAACACATGGTATTAGGTATCAAG

TTCCAAACCTATACCACCTTGATTTCATTCCTGATTCCAAACCCATACCACTATGTGAAC

CAAACGCCCCC

>Tourist|21.1

GGCCTTGTTTGGTTGTATATAGGAAGTCATACTTCCTAGGAAGTTATTTTTTCCATGTAT

TTGAAATTCCTAGGAAGTGTAAAATTGTGTTTGGTTGATAATGGGAAGTAGAACTTCCTA

TGGGAAGCTCTACTTACCTAGGGGGACCTAGGTAAGTAACTTCCTCCATTTTGGAGGAAG

TAGAACTTCCTAGGAAGTGCAAATTCCCATGGTTTTGCCAACCAAACAACTTCATATACT

TCTACTTCCTAGGAATTTGCACTTCCCATGATTTGTTTTGTCAACCAAACAAGGCC

>hAT|1.1

TAGGGATGGAAATGGATCGGATTCGGGGCGGGTGGAACATCACCCGCATCCACCACCCGC

AATTGTTTACTCCACCCGCATCCATCCATCCGTCATCCATCGGGTGAAACGGGTGGGTCG

GGTGCTAAACGGGTGATGATATTTAGTATATTTTTAAAAGAAATTAGTGACATAATATAA

AATAATTATATCAAAAACATAAAATATAAGTAATTTACATGTAAAATTTAATAATTGGGT

AATGAGTTGGATGTTAAACGGGTGATGGATCGGGTGACGGATGAAAAAATTCTCACCCAC

CACCCACTTTCCATCCATTTAAATTCGGGTTTTCATCCATTTAAAATACCGGGTTAAGCG

GGTGATGGATCATCCATCACCCATTTCCATCCTTA

>hAT|2.1

TAGGGGTGAGCACAACCGGGTACCCAGTAGAATTTCATAGACCCGGACCCAGTGGGTATT

AAAAATCTGGAATTGGAATTGGTCAAACCGGGTCCTAAATTTTTGGACCCAGACCCGGAC

CCGGTGGGTACCAGGTCCAGAACTGGGTACCCAGTTGTAAGTTTAACAACATACTAAGAT

GGGCCAATTGAGCTATAAAATTTTTTAAAAAAATTAAATGGGCCTAAAGGCCTACTAAGT

ACTAACTTTTTACTTAACAACTATTATCCTATTACCAATTCTATATGAGTATATGAAAAA

AACCAATTCTAAGTACCGGGTACCCGGACCTAGACCCGCTCCAACCGGGTACCCAGAGCT

GGAACCGGTCAAACTGGGTACCCACCAGGTACCGGGTCCGAAAAAATTCTACCCAGAATT

GGAACCGGATGCAACTGGGTCCAATTTTTTGGACCCAGACCCGGACCCAGTTGGACCGGG

ACCGGGTCCGGACCCGGCGGGTCCATACCGGCCGGGTCTGGGTCTGGGTACCCGGAATTT

CTGCTCACCCCTA

>hAT|3.1

TAGGGATGGCAATCCAATCCGAATCCGACCCTAGTCCGCTCCGATCCGAAGGAAAAAATC

CGAGTTGACTCCGACAAAAAAGTAATCCGACTCCGACTCCGTTTAAAAAATCCGACTCCG

AGTCGGAGGAGGGTCGGAGTCGGATCTCATAAAATAATCCGACATCCGAATCCGACTCCG

ACTTTGAGTCAATCAATCCTTTTTTCTTATTTATGTAATGTATTATTTATGTATAATCTG

ACTTCGACTTTCAGCCAGTTGTATTATATAGGCTTTTCCCACAGAAAAAATGATTAAACA

ATTGGAAGAAAAATAAGTCGGATCAGGTTCGGAGTCGGATGTTCGGATTTTAAGTCGGAT

GTCCGGATTTTAAGTCGGGTCGGATCTCGGAGTCGGATGTTCGGATTTTAGGTCGGGTCG

GATTCGGATTTTCAAAGTCGGATCTCGGAGTCGGAGTCGGACTCGGATTTAAAATCCGAC

TTTCTGTAGGGTCGGAGTCGGATTATGCATAATCCGGTCCAAGTCCGACCCGTTGCCATT

CCTA

>hAT|4.1

TAGGAATGGCAACGGGTCGGACTTGGACCGGATTATGCATAATCCGACTCCGACCCTACA

GAAAGTCGGATTTTAAATCTGAGTCCGACTCCGACATCCGACTTTGAAAATCCGAATCCG

ACCCGACTTAAAATCCGAACATCCGACTCCGAGATCCGACCCGACTTAAAATCCGGACAT

CCGACTTAAAATCCGAACATCCGACTCCGAACCTGATCCGACTTATTTTTCTTCCAATTT

TTTATAATAGAATAGTCTTCTATAATACAATAATTTAATCATTACAATTTATTCTAATCA

TCATATGGAAATCTTATATAGTACAAATAATCTAATTATTACTATTATTTTTAACAATCA

TTTTTTCTATGGGAAAATCCTATATAATACAATTGGCTGAAAGTCGAAGTCAGATTATAC

ATAAATAATACATTACATAAATAAGAAAAAAGGATTGATTGACTCAAAGTCGGAGTCGGA

TTCGGATGTCGGATTATTTTATGAGATCCGACTCCGACCCTCCTCCGACTCGGAGTCGGA

TTTTTTAAACGGAGTCGGAGTCGGATTACTTTTTTGTCGGAGTCAACTCGGATTTTTTCC

TTCGGATCGGAGCGGACTAGGGTCGGATTCGGACTGGATTGCCATCCCTA

>hAT|5.1

TAGGGCTGGCAATCCCTGACACGACCTGATAACACGACACGAACCCGACCTGAAGTTACT

GAGAGAAACACGAACACGAATGACACGAAGTCAACCCGATTAGTTACATTTATCATATTA

TGCAACTATTCTACTTTTTATATCATTTTTGATACATTTGGGGGCCAACCCGAAATCGAC

ACGAAACCAAACACGAACACGACCCACTGACACGAATTGCCAGCCCTA

>hAT|6.1

TAGACCTGGCAATCGGACCAATCGGATCGGATTCGGACCGGACCAATTCAGATCGGACCG

TTTTCGGATCAAGTTACATTCGGACTGGGATCGGACCGGATCGGACCGTTTCGGATTGGG

TCATCGTTCGGACTATTATTTCGGATCGGAATCGGACCGGACCAATTCAGATCGGACCGT

TTTCGGATCAAGTTACATTCGGACCGGGATCGGACCGGATCGGATCGTTTCGGATTGGGT

CATCGTTCGGACTATTATTTCGGATCGGAATCGGACTATGGTGGAATGAATGGAATACTT

ATGCCAATGATATTTTTTTTATATAAAAAAACGGGTAAGTTTCGGGTATTTCGGATCAAA

GCCTAATCGAAACCAATAATTTCGGATCGGTTTACCTTCGGGTCGGATCAGTTTTAGATC

GGGTCGGATCGGTTTCGGATCGGGTAAGATTATATCGGATCATTCGGATTTCGGATGAGT

TTCGGATCAAGATTATTTCGGATCGGGATTCGGACCGGATCGGGACCGTTTCGGATTCGG

ATCAAGGTTCGGACCGTCTCGTTCGGACCCAGATCGGACCACGGATCGGATATTTCGGAC

CGGATTTTCGGATCAGGACCAACTTTGCCAGGTCTA

>hAT|7.1

TAGGGGTGTAAGTGGATTGGACTGGATTGGACTTTGCCAAATCCAAATCCAGTCCAAAGT

TTTTTGGACTCGAGAAATTGAGTCCAAGTCCGATCCAAATATTTTTTGAGTCCAGTCCAA

TCCAGTCCGATAATTTTTTCTTGAGTCCGAATCCAGTCCAGTCCAGTCCGATTATTATAT

CTTTTTTCCCGATTTAGATTCAATGATTCACAACGTTTTTTGAGATGCTTGAGCATTTGA

CATCTGATTCAATTATCAATATCCACAAATTAGATTGAAAGCTTAAATTAAAGTAAAATA

CTATGAATAAAAAGTTGAATTAGATGCTTACCTTGATCTAAGTTGAGAGGAAGCAGAGAG

ACTGAGAATTAATCTGAGGGAGAAATAGAGAATGCGAGAGTCGAGACAGCGAGGTAGAAA

GAAAATGAAGAGTAAGAGGAAGTGAGTAGTAAGGACTGAGGAGTGAAGTGAGATAGAATT

AGTCGGCTACTAGCCTACTACTGCAGTACTGCTAGTATAATTTACTTATTTAACAAATGG

AGCTAAGTGCAATAGTCTGGCGCCAAGTAAAATTGTCTGGCGCCAGTTGAAATATTTAGA

GAGGGAAGGTTGAAAACTCCAAAATTTTAAAAATAGTATCAATTTTTTTAATATATACAT

TATATATGTATTAAATATATATTATATATAAAAATATTTTTGGACTGGACTGGACTTATT

GGACTCCAAAGGGATGAGTCCAAATCCAGTCCAAAAATATTTGGACTCGAAAATTTGAGT

CCGAGTCCAGTCCGAAAAATTTTCAGTCCAGTCCAGTCCGACAAGTTTGGACTGGACTGG

ATTGGACTCTGGACTTTTCGTAGTCCGCTTACACCCCTA

>hAT|8.1

TAGAGATGGCAATATCCAGTGGATCACCCGACCATCCGCTCCACCCGGTTTATTAAGTGG

GTGAAAAACCCGGTTTAAACGGATGATAGCCGGGTGACGGATAATCGGGTGATGGATGCG

GGTCGGGTTCGGGTGATATTTTTTCATCCATCACCCGACCCATCATCCGTTTACCACCCG

ATCCGTCACCCGTTTAAAGTAAAAAATATATAATTTTTTTAATTTTGGGCTTTTTCATTT

TTACAGCTTTATTAATCTATAGCCCAAAAGTTACAAGATGATAATATAAGTATATAAATG

TTAATAATGTGGATTAGGGTTAGTTTGGAGACGATTTTACATAGTAAGTAGAATTATATA

AATGGATTAGTGGTTCTTGTTAGTAATTTTTCTTTCTTCTCCTAATACTATTTGATTTCA

CCCGTTAATCACCCGATCCATCCGTTTAACCCGGTTACAAATGGATGGATGGATTCGGGT

GGAGATGGATGCGGGTGATGGATGGATCGGGTGGAGTGGGTGGATGCGGGTGACGCTCCA

CCCGCCCCGAACCCGATCCATTTACATCTCTA

>hAT|9.1

TAGGGGTGAAAGTTCGGTGTGCGGTTTGGATTAGGGCCTAATCCAATCCGATCCGAATAA

AATTTGGATTTCAATTTTTCAATCCGAATCCAAACCAATTAGGGTTTGAATCCGATCCAA

TCCAATCCGAAATTTCATACTCTAGATTGGATCCGATCCGAAAATCCGATTTTTTTAAAA

CTTTAATTTTTCGATGAAATAAATTAAGCACAACAGCAGCCTAGTAGATTTAGGAAATGA

GGAATTAAGTGCAGTTAGTGCTTTAGGGGAAGGATTGAAGTTCTATGTGTATTGGACTAT

GGAGTAGGTTTTTTTATAATAAAAAATCAGTTAAGCACTTAAGCCCAATCCAGATACATA

ATACAAGGCATATTACTATTCGGTTATATTGGATTTTTGGATCCAATTACCCTCAATCCA

ATCCGATCCGAAAATCCAATTTTTCAAAAATTCTCAATCCGAATCCAATCCGAACACAGT

TCGAATCCGATCCGAATTACTAATTTGGATTGGATTGGATTGGATTTTGGATTTTATCCG

AATAACTTTCACCCCTA

>hAT|10.1

TAGTGATGGCCACTGGTGCGGGCTGGGCGGGCCGGACCGTGGGCCGTAGCGGGCTGGGCT

GAGATTTCACGGCCCAGGACCGGACCGTGAAATTCATGGGTTGGGTGGGCTGGTCCGCGG

GCTGAAAAATCTTGGCCCACACCGGTCTGTTACCGGTTTTCACGGTTCGAGAAGCGGTTC

TGGCGGTTCGTGGGTCTAGTGGGCTGGACCGGGCTGGCGGGCCGGCATGAGAATTTTTTT

TTCAAAATTATTTAATCATTGAAATTATTCATTATTGTTACATAAAATCTTTAAAATGAA

TTAAAATATATACAATTATTAATATTAATGAATAATTTCATTATTTTAAAATTTGATTGA

ATTATAATATATGCAATTTGATTAAAATATACACAATTTGGTTCAATATTTACTTGGACT

GGGCTGGTCCGGTTCATGGGTCGGGCTGGACCGGTTTCGTGGTCCGGGCTGGGCGGTTTT

AGTAGCGGGCTTTAGTGGGCTGGGCTCAAGAAATCTGGCCCACGACCCACCCACCAATTT

CACGGTTCGAGCTGGGCTGGACCGGGCTTTTTGCGAGCCCGACCCACACGGCCCACGAGG

TGGGCTGGGTGGGCTGGATTGGCCCGCACTGTTGGCCATCTCTA

>hAT|11.1

TAGGGATGGCAGGTGGGTATAATTTGGCGGGTTTCGCACCGCATCCGCCCCAGTTGGGGC

GGGTATGGATATGACTTTGGTGGGTAGTGGGTGGGTATGGATACGAAAAGTCAAATCCGC

CATGGGTAGTGGGTAGGTGGTGGATATGAGGTCACATCCGCCCCAAATCCGCCCGCCCCG

CCCCATATCCATCCGCCCCGTATAATATTTACTAATTTACCCTTTATAAAATAAAGAATT

ATGTTCATAAATCTAAAAAATTGTAAAAAATTGAATTGAGTGATGTTTCTTGAGATTTAT

TAGTAACAATGAAACTGTATTGTATAATTGTATTTGTTTGTTAGTGTTTTGTGAACTTTG

TATTGTCATTTGGAACAATTTTCAACTGTTCTTGAGGTTGAACTACTGCACTTTATGTAT

GACAAAGAATGTGTACTTTTTTTTCTTTGAAATGGGTGTTTCTAATAATTAATTTATATA

ATAGGTTATATATAGAAAATAAATCAGGTGGGCGGGTGGAAATCCACCTGGGTGATGGGC

GGGTATAAGTGGGTAATGGATATCCACTTGGGTGGTGGAGCGGATGTGGGTATAACCTTA

TACCCACGTGGGCGGAGGTGGATATGGAAACTTGTACCCGCCATGGTTGGCGGGCAGGTG

GCGGGTATGGAAAATTTAGGTGGAGGTGGGTGTGGATTTAGGGGTATCCGCATCCGCCCC

GCCCATTTGCCATGCCTA

>hAT|12.1

TAGGGATGGCAATATCCAGTGGATATCCAGTAACCCGCTCCATCCTGTTTAAATAAAAGG

GTGAAAAACCGTAATAAACGGATGAAGACCGGGTGACGGTGAACCGGGTGACGGATCCGG

GTAGGATGCGGGTGACCATTTCTCACCCATCACCCGATCCACCACCCGGTTAAGCACCCG

ACCCATTATCCATCCGTTTAATTTTTTTTGTTCAAACTCGTATTTTTGAAACTCGTATTG

TTGAAGTATTGTTGTCGAGACTTTATTATTGTGAAGACGACTTCCTAAAACTTGTATGGT

GCGACGTCACCTTTTCTTTATGCTAAAAACATCGGCGTATTTATTTATGATTTCGGAAAG

TGTAGGTGGAAATTGGGTACATTATTTTTTTAATAGTTATAATCTAAATTTGATATTTAG

ATTTTTCACCCGTTAATCACCCGACCCACCCGAATCACCCGGTTGTGATGGATGGATGGA

TTTCGGGTGATGAGTAAAGCGGGTGACGGATGGATGGATGAGAGTCAATCTAACCGGGTG

ATGGATGCGGGTGATGGCTCACCCGACCCAAATCCCACCCGTTGCCATCCCTA

>hAT|13.1

TAGAGATGGCAATGGGTAGGATCTGGACAGGGTCCTCTAGGATCCAGATCCAGATCCGTT

TTTTAGGCAAAGATCCATATCCTACCCGGATCCGCTGGGTAATAAGAATGAGGATCCAGA

TCCAGATCCGTATGGATCCTACGGATCCGGGTCCTAAATGGATCCAAAACGGATCCTTGC

TTATTGTCTTCAAACTATCCTAATTTTCACAATAATTAAAGATTTTAAACAATAAAGTCA

TCTAATATAAACCAAAAAATACATTTTATTTTTGAAAAATAACCATTGTAACTACTTACA

ACTATTCAATTATCAAAATGTAGATTAGTTATCAACCCAATTTTCGAACTTAACTATCCA

GTGAATTCGCACAAAAATCGCACATTAATTGGAACAAATCCAAAATTCAAGCGAAAATCC

GTTTTACATGTAATAACTAATTTTATCAAAATATAATATTAAATTATAATAATATTATAA

AAACGGGTAAACGGATCTGGATCTGGGTAATTATTTAGGACCCGGTCCAGATCCATATTT

TTCAAGGAGGATCCAGATCCTACCCGGATCCATAGGGTCCAAAATTTGAGGATCCAGATC

CGTCAAAAATGGATCTGGATCCACGGATCCGGGTAGGGTCCATTACCCACTGCCATCTCT

A

>hAT|14.1

TAGAGATGGAAATGGATCGGGTTCGGGGCGGGTGGATCGTCACCCGCATCCATCACCCGC

ATCCACCCGCTTCACCCGATCCATCCATCACCCGCATAAATGTTCACCCGAATCCATCCA

TCCATCACCCGCGGGTGAAACGGATGGATCGGGTGATTATCGGGTGAAGGGAGAAAAAAA

TCCAGTAGCCCAACAACATAAAACCCACAACAATTTGCATAAGAAAATAAGTTATATATA

TATATATATATATATATAAACGGGTGATGGGTCGGGTGGTAACCGGGTGATGGACCGGGT

GATGGATGAAAAATTATCACCCGAATCCGTCACCCGTTAACCCGTCACCCGCCTCTCATC

CGTTTAAACCGGGTTTTCCATCCGTTTAATAGTGCGGGTGAAGCGGGTGATCGGATCATC

CGCTGGATATTTCCATCTCTA

>hAT|15.1

TAGGGCTGTTCAAAGTGCGGTCTGGACCGCACCAAACCGCAACCCAAACCGTTTTTTCGC

GGTTTGGGTTGGTTTGCGGTTTTAAAATTGCGGTTTGGGTTATGATTTCAAGCAAACCGC

GGTTTGCGGTTTGGGTTGGGTTTTAATTTTTGTAAACCAAAACCGCACCGCAAACCGCAA

TGTTACATATTTTAAAAAAAAATAAAATTAAATACACTTATGAAGGTGACATACAAGTAT

AAAATTGAAAAAAGAAGTTTGAGGTAAAAAACTTTAACACTTTTGATAAATCATTATATA

TGTTTAATTATGAATTCAACTTCATATCTATTTGGACTCTTATTAACAATTTTTTTTTAT

CTTAGATTACAAAAGTAATGTCGCGGAGGAAAAAATGGTTAAACCGCAACCCAAACCGCA

CCAAACCGTTTTGCGCGGTTTGGGTTGGGTTGGTTTGGGAAAAATTGCGGTGCGGTTTGG

GTTGGAAAATTTTCAAACCGTATATTTGCGGTTTGGGTTGGGTTACATCCCAAACCGCAC

CAACCCAAACCGCGAACACCCCTA

>hAT|16.1

TAGAGCTGGCCACGGGCCGTGCTGGGCCTGACCACGGGCCAGGCCCGCGGGCTGAAGTGG

AATACGGGCCGGGCCAGGCCCGTGGCCCATCAAACTCGTGCCGTGCCGGGCCGGGCTGAA

ATTATGGGAAACTTGTCCATGGCCCGGCCCGGCCCACGGGCCTGGCGGGCCGGGCTCGTG

CTCACCGCGGGCTTGGTCCAAAAATCACAGTTTTTGAAAAAAAAAAATTGCAAGGCCCAC

GGGCTCGTGCCGGGCCCGGGCCGAAATCTAAAATCTCCAAACGTGCAAGGCCCACGGCCC

ATGTTATGGCCAGCGGGCCGGGCCGGGCCCATAGCGGGCTCGGGCCGGGCCGGGCCGAAA

ACGGGCCGGGCTCGGGCAGCCCACGGGCCAGCCCGGCCCATGGCCACCTTTA

>hAT|17.1

TAGACCTGGTTATTGGACGGGTCGGGTCAGGGTCAGTGCGGGTCGGAAGCGGGTCGGGTC

ATAAGAGGGTCGCCTCATAAAGGGTCGGTAGCGGGTCGGGCCAAATGGGTAGAGGGCTTA

TAGCGGGCCGATAATGGGCGGGTCGGTAAACGAGCAAGAAAAACAAGGAACAAAAAGAGC

CAAAAGTAAGTATAAATGAATGCGAATCCGAATATGTAATTGTTTTTACAAAACTTATTT

TATTTTTTAAAATTATTGGGTTTCTTTGTTGACCCTATAATGGCCCTATCCAATAATGAC

CCTGTCCAATAAAACCCTGTCCAATAAAAGCCCTGTAAAATTGACCCTAACCCTTTAACC

ATTGGGCTACCCTGTCCAATAACCACCTCTA

>hAT|18.1

TAGAGCTGGGCATGGGCCGGGCCGGCACGAGGGCCGGCACGGCACGACACGATTTGAGCC

CGGCCCAGCACGAGCCCGCTATGAGCACGGCACGGCACGCCGGCACGAGTGGTGGGCCGT

GGGCCATGCATGAACAACATTCTAAAAGTTTGGCACGAACCCGGCACGAGCACGACGGGT

CGGCCCACAAGTTGGCACGCTTATTTTGGTTAAAATTTCATAATATAAATTAAAATTTGT

ATAAGGCACGATGGCACGGCACGAGCCGGCCCGGCACGGCACGTGGGCCGGGTCGTGCAT

GGGCCTTAGATTTTACAACTTGGCACGAGCCCGGCACGACACGAGCATTTCTTGGCCCGT

TTTGGCACGGCACGTTGTGGCACGAGGCACGCGGGCTTGGCACGTGGGCCGTCCCGGCAC

GGCCCGTGCCCACCTCTA

>hAT|19.1

TAGGGCTGGCAATCAGGTCGGGTTGGGTCAGTTTCAGGTTGACCTGATTTATATTTCAGG

TCACTTACCCTTGACCCAAACCCGACCTGATTTATATTTCAGGTCGAAAACCTCAACCCA

AACCCGACCTTATATTTTTCGGGTTGACCCGAAACCAACCCAAAATGACCTGAAATAAAA

ATCTAAATATCCAATGATAAAAAAACTGCCAACTTAATTCAGAAAGGTATTGTACATGTA

ACATGTTTAAGTTTCAACATTCAAAGCCGGAATTTTCAGGTTGAAGTGAGTTGGGCTTGG

CCCAATATTATAACCTAACCGGCTAAGGGCTAACCTAACATAATATAAGTTATTTTTAAA

TCAGGTCATTTCGGGTTATTTTCAGGTTCAAAAGGTCAACCTGAACCTAACCCATTATAT

TTTCAGGTCGGGTTGGGTCGACCCATTTTTTTTTCAGGTCGAAAATCTCAACCCAAACCC

GGAATTTTCAGGTCAGTTTCAGGTCGGGTTATCAGGTTGGGTCAGTTTTTGCCAGCCCTA

>hAT|20.1

TAGGGCTGGCAATGGGTCGGGTTGGGTCAAGATCAGGTTGGGTTGGGGGTTGGCGGGTTG

AGAACCCCTTGACCCAACCTGACCTGTTAACTTAAACGGGTTGAGATTTACAACCCAACC

CAACCTTATGATAAACGGGTCAACCTTCAGGTCGACCCGTTTAATTTTTTTTCGTGTTTT

TTCTAGAAAAAAAAATAAAAACAACACAATTTATTTGCAAATAACAGTAGGCAGCAAATT

AAAATTAAGTCATTACATAACCATATGATAATATTGTTCAAAATCAACTAACTTTTGTTC

AAAATCAACACAAATGTTCATTTAGGTGGAAATCTGAAATCCCTAACCTTTTAAGTAGTG

CTAGGGTTTTTACTTTATTACTTTATGGTCCAGCCCAATTTTTTTGGGCTGAAATCTGAA

TTGAGTTAGGGATTTTTTTAGCTTGTTTTTTCATAAATGGGTTAGGTGGGTCAAAATCAG

GTTGGAATTTTCAACCTGACCCAACCCATTTAATTAAACGGGTTGGGTCAGGTCGACCCA

TTTAATTAAACGGGTTGAAATTTTTGACCCAACCCATTATTAATGGGTTGGGTTCGGGTC

GGGTTGGCGGGTTGGGTAATATTTTGCCAGCTCTA

>hAT|21.1

TAGAGGTGATCATGGGCCGGGCCGGGCCGCGGCCAAAAAAATTTGCCCATTGGTCGGGCC

GGGCCGGGCCTAAATTCGGGCCGAATTTTTGTGTCCAAAGCCCGCTATTTCGGGCCTAAA

TTAGCGGGCTTTTGCGGGCCAAAACGGGCCGGGCCAAAAAATAGACTAAAAAGTCGTTTT

TGGCTTGCCCAAAGCCCGCAAAAAAAAAATACGACCGGGCCGGGCCCGCGGGCCGAAAAA

TTCTGCCCAAAACCCGGCCCAAAAAGGGCGGGCCGGGCCAAAATGATCACCTCTA

>uc|9.1

TACTCCCTCTGTATTTTTTTATCTTTCCTACTTTGACTTTGCACGTTTGCCAATGCACTT

ATTTTACATTAAATATCTTTCATTACATATTAGTAAAAATTATAATAATTGAATATTAAT

AAAGTACTCATTGAGACGAATCAAACAAGACCCCACATGACTATATTTTTTCTTATATAT

TGAGAAAAATCTAAAAGATTCTCTCTAATTGTGAATAGTGTCAAGATTCTAAGTGAGAAA

GATAAAAAAATACAGAGGGAGT

>hAT|22.1

TAGGGGTGTGCAAAAATATCCGATCCGAAAATCCGCCGATCCGATCCGAAATTTCGGATA

TCCGATCCGATTTTAAGATTTCGGATATTTCGGATCGGGTATCCGGAAAAAATTTCGGGT

TTTCGGATCGGATACGGATCGGGATTTTTCAAAAATTTCAACGGACATCCGATCCGAAAA

CCCGGTGATCCGATCCGAAAAGTTCGGATATCCGAAAATTTCGGATCGGATTCGGATCGC

AATTTCCCCGATCCGAAATTTTCGGATATCCGAATTTTCGGATCGGATCCGATCCGATAT

CCGACATTGAACACCCCTA

>hAT|23.1

CAGGGCCGGTTCTGAGGGTAGGCAAGGAGTGCCACCGCCCAGGGCCCACGATCGTGTGGG

GCCCAAAAAAAGAAATTAACTATGACCAGTTAAATAAAAATTGATACACTTTTTGAATAT

TTAGTGAAATTTGTGCTATTTTTGTGCAATTTTTAATAATATTATGTGCTTATTTTTTAT

TGGTATCATTAACAAAATATAGATTATAGTTTTAGGTACGAAGGTTTTAATTATATGACC

TATAAAATATTAGGATCGACTCATATAGGGGCCTTTATTTTTAGGTCCGCCTAGGGCCCA

AAAATTATCAGGAACGGCCCTG

>hAT|24.1

CAGTGTTACCTAATTCCCTAATTCCCCCGCGAATCGCGAATTGAGAAAAGCGAATTATGG

TCGGTTTTGGTTCATTTTTGGCTCATTTTGGGCGAATTGGCGAATTCAAAAGGCGAATTA

GATAGCGAATTAGGTAACACTG

>hAT|25.1

CAATGTTGTTAAAATCGAGATTCTAGTTTGAATCGAAAACAACCTTTAGAATCGAATCGT

AAAATCGAATCGTAGAATCGTAAGATTCTACAAACATGTATAATCAAGATATTGCACTCA

ATCTTCATTGAATATGCTAAAATTATAAGTTAAAAGACCTAAAAATTGGAAAAAATAGTT

GTTTACCCACTATATTGGCAATAACTAACAATATATATCTCTTATTTTCTTTGTTAGCAT

GATTTAAACTTGCGAGGTTTTCGCTAGTCAACAGAAATGTATAGAATCGTCTAGAATCGC

GAATCGAATCGTCTACCATATTTTGAATCGTAAAATCGTAAGATTTTAAGATTTGAATCG

CGATTTTAATAACTTTG

>hAT|26.1

CAGGGAATTAAAACTCTCCCGTTCCGTTACAGAACGGCCGTTCTGTAACGGATTTTGGAT

TTGCCGTTCCGCGAAATCCCGTTACGGAACGCCATAATAAAATTCACGTACTTCTCGCCG

CGTTGTCCGTTCCGTAACGCGAACTCGCCGCGTTAGAACGATTTTATCCCCGTTACATCC

GTTCTTTTCAAACATTGGCCAAATTTTTTTTATGATTTAATTATATAGTTGAGGAAACAT

GATATTCTATTAGATTTGATTATATTATGTTGTAATAATGCTTCATAAACTAAAAAAATG

CAAATAAGGTTAAAACATATTAATGTGAATTTTTTAAAAAAATTCACGCCGCGTCGGCCG

CGAAACGTTCTATTTCGCCGTTACAAGCCGTTCCCGCGACAACGCGACCGTTCCCGCGAA

CGCGACCGCGAGCGCGTTTTTTTTCCCTG

>hAT|27.1

CTATGTTGCTCAGACTCGGGTACCCATGTCCGACACGGGTACGTGTCCAAGTGTCCGACT

CGGCTATTTTTTGGAATTTTTTCTATTTTTTAGCCCAAAACAAAGTGTCCAAGTGTCCCA

CCCATGTCCGAGTGTCGAGGATCCGACACGGGTACTTGAGGTGAAATGAAGAGTCCGAGC

AACATAG

>hAT|28.1

CAGTGTTACTCCGACACCTTCATTTACCTCAAAAACGGGGTGTCGTGTCGTGTCGTGTCC

GACACGACACTTTACTTGGATACTTCATTTTAAACTAAAAACTTGACTTTTTTCTACAAA

ATAGCCGAGTCGGACACTTGGACACCCGTATCCGAGTAGACACTCAATCCGTGTCTGAGT

AACATAG

>Mutator|1.1

GGGAAATTCCCACTGGTAACCCTGTACTATGGAACAATTGCAATGGTAAACCGTTTTTCT

TTTGAGTTTCAAAAATAACCCTATACTTAGTAATATTATACAACTGTGACATTAATGATG

GTTTAATCGTTACTTCAACCATTAGTATTATTAGTTTACCTAACCCAGATTAAATATTAA

CTAATATAAATAATATGTTATTCAATACTTAATCATAGTGAAGTAAATCTAATAATACTA

ATGGTTGAAGTAACGATTAAACCATCATTAATGTCACAGTTGTATAATATTACTAAGTAT

AGGGTTATTTTTGAAACTCAAAAGAAAAACGGTTTACCATTGCAATTGTTCCATAGTACA

GGGTTACCAGTGGGAATTTCCC

>Mutator|2.1

GGAAAAATTGTTTTAAATAAGTCAACCTTTGCGCGGTCTTCCCATTTTAAGTCTACCTTT

TGATTATCCCTGATTGAGTTAGGCTTGTATGTCTGTTTTCTTCTATTGAGTTTTAACCTG

TTTGTGACTTGTTTAGTAGGTTACTTATGATACGTGTTACAATGTCCCTTCTTCTAAAAC

AGGCGAGGGTGGGGGTGGGACATTGTAACACGTATCATAAGTAACCTACTAAACAAGTCA

CAAACAGGTTAAAACTCAATAGAAGAAAACAGACATACAAGCCTAACTCAATCAGGGATA

ATCAAAAGGTAGACTTAAAATGGGAAGACCGCGCAAAGGTTGACTTATTTAAAACAATTT

TTCC

>Mutator|3.1

GGAAAAATTCACAAGAGAGAGCCCAACTATTACCCATTTTCTTTTATAAGTCCCAACTAT

GGATTATTCCCTACAGGTCCCAAGTTTACTATCTTGTGATTTTCATAGGTCCTGAGTTAC

TTATAACCGGTTTAGAAGGTAAGATTTTTAAATGTTTACCATCTTCGAACACTTTCAGCA

AGTGACAAAGACCTATAAAATGGGGCCAACATTTAAAAATCTTACCTTTTAAACCGTTTA

TAAGTAATTCAGGACTTATAAAAATCACAAGATGGTAAACTTGGGACCTGCAGGGAATAA

TCCATAGTTGGGACTTATAAAAGAAAATGGGTAATAGTTGGGCCCTCTCTTGTGAATTTC

TCC

>Mutator|4.1

GGGAAATTTCCTGAGGTACCCTTGAGGTTTGGCTTAATTCTCAAAATACCCTTATTGAAT

TTTGAAGTTCAAAGTTTAAGTTTCAAGGGTACCTTAGGAAATTAATGCACTTGAATTTCG

GTGTATGATAAGGGTATTTTGAGCCCAAACACAAAGTTCAGGGGTATTTGAGGAATTCCC

CAAATGTGAGGGGTAATTTGAGAATTACGTCAAACCTCAGGGGTACCTGAGGAAATTTCC

C

>Mutator|5.1

GGGTTTTTTGTCAGAAAGGACCTAATAAAATACAAGTTTTGTGGGGAAAGACCTAATAAA

ATTTTTTTTGTCTAAAAGGACCTAATAAAATACTTCCGATTGTGAACAGGAACCACTTTA

TGAATTTCCGGCCAAATTTACCATTTACCGGCGTTGACCCTTTTTTTTACCCCTAGTTGA

CTCTTTAATGTTCTGTTTCTATTTTACAGCACACAAATCCCTATTGAATCCCCAAACTTA

ATGTTCCCGTTGGCTTAACCTTCTCCTTCTTCATCTTTAATTTCAACTTCAACTTCTCAA

ATTGTGCTTCAACTTTCAATTTGTCAGCCTTCATCAAAGCCATTTCTTGCTTTACCACCT

TCAACTCATTCAACAAAAACTTCTTCTCATCAACAAGATCATTAATCACAATTCGTTGCC

ATTCTGTCATCTCATCATCCACCCATCTAAAGAATTTGCATCCCCTTGGATTTAAAATAG

GAACAGAACATTAAAGAGTCAACTAGGGGTAAAAAAAAAGGTCAACGCCGATAAATGGTA

AATTTGGCCGGAAATTCATAAAGTGGTTCCTGTTCACAATCGGAAGTATTTTATTAGGTC

CTTTTTGACAAAAAAATTTTTATTAGGTCCTTCCCCACAAAACTTGTATTTTATTAGGTC

CTTTCTGACAAAAAACCC

>Mutator|6.1

GGGTTTTTCTACATAGTACCCTTGTGTTTTACGAAATTCTATGTTGTACCCGACGTTTTT

AAAATTTCCACGTTGTACCCCTAAGATTTCTTAACGTTACCCACAATACCCTTATACACG

ATATCAAACAATAGATTTTGGGACTTTCTTACCTTGTGGGGATAGAGTAGGATGTTTTAG

CCTTGTTAGTTGACTGAGGTTTGGGATATGATCTGATAAATTGAAAACTAAGTGAGGTTT

GGAGTAGGATTTTCTATTAGTAAGGGTATTTTGTGTGACGTTTAGAAAGTTTAGGGGTAC

AATGTGGAGAGTTCAAAAAGGCTGGGTACAACGTAGAATTTCGAAAAACACAGGGGCACT

ACGTAGAAAATCCC

>Mutator|7.1

GGGTTTTTCTACGTTATACCCTTGTATTTTTTCAATTTCTACGTTGTACCCCAAGCTTTT

CAAAATTCCACACAGTACCCTTAAACTTACCTTTTTCAACGGACAATGCCCCTACCGCTG

ATTATACATCATTAATTTCGTCTATTTTTTATATTCCGGTGGAACGAATCTAATTAGTGA

ATAACCTTCTAAGGGTTAAACTATGTATAAGTCAAATTAGTTCCAATAAATTTAAAAAAA

TAAATGAAATTAATGACATATAATCAGCAGTTTGGGCATCGTGCATTAAAAAAGGTAAGC

TTGAGGGTACCGTGTAGAATTTTTAAAAAGTTGGGGTAAAACGTAGAAATTGAAGAAACA

CAAGGGTATAACGTAGAAAAACCC

>Mutator|8.1

GGGAAATTCCCACTGGTAACCCTGTACTATGGAACAATTGCAATGGTAAACCGTTTTTCT

TTTGAGTTTCAAAAATAACCCTATACTTAGTAATATTATACAACCGTGACATTAATGATG

GTTTAATCGTTACTTCAACCATTAGTGTTATTGGTTTACCTAACCCAGGTTAACCCCAAC

TCCCAACTACCACCCAGCCCACTGACATCCCATCCAACCACCACAGTTCCACCCCTCCAC

GATTGTCCCACCGCTCCACATGAACCCCACTCCACCACCAATGTCCCACTTCTCCACTAC

TGTCCCACCTCTCCACCAATGAGAAGAGAAGTCACAGGGAGGGAGAGAGTAACGAGGAGA

GAGAAAAAGTCATCGGGAGGGAGAGAGTGACGGGGTATGGTGTTTCCGGTGGAAGGATGG

CGGTGGGGTGGGGATTTAGTGGGTTAGGTAGTGGTTTGGGGTTAACTTGGGTTAGGATAG

TAAATGGGTTTAACGGTTGAAGTAACGATTTAACCATCATTAATGTCACGGTTGTATAAT

ATTACTAAGTATAGGGTTATTTTTGAAACTCAAAAGAAAAACGGTTTACCATTGCAATTG

TTCCATAGTACAGGGTTACCAGTGGGAATTTCCC

>Mutator|9.1

GGGAAATTCTCACTGGTACCATCCAACTATCGACTTTTCTCAATGGTAGGCATTATTCTT

TTACCTTCCCAATGGTACCACTATGTTTATGACTTCTTAATTAAATTGATTTTTTTATTA

AAAATAAGTTAAAATTATTAATGGCTATAAAGTAGAAATACAATCCTACCCATTATCTTA

ACTTTTCCTCCTATAATTCAACATCCCAACGATTCATCACATTCCAATTAACAGAGGAAG

GTCCAATTTTGATGAGGATGAATTTTTGGTGTTGGGTAGTGCGTATGGCAGCCAAGTATT

AGATTAGGGTAAATATTCTTTTTTTTTAATATTTATTAGTTACATTTAAAGTTTAGCCTA

ATTTCTGTTAACAATAAAACACTTTAAATAAAAAGTCATAAATATAGTGGTATTATTGGG

AAGATAATAAAAAAATGTCTACCATTGAGAAAAGTCGATAGTTGGATGGTACTAGTGAGA

ATTTCCC

>Mutator|11.1

GGATAATTTGTCAAAAGGGACCTTAGAATTTTTTGTTTTTGCGAGAAGGGACCTTAAAAA

AAAAGTTGGCATTTAAAGGCCAAACAGCAAAAAATATTGTGACGAAGGACCTTACCACCG

GTTTCCGACGTTGACCAATCTTTTCAGGCATTGACTTGCCTCAATTTCTAAATGACACTC

CCTAACTTTATAACTGCACTCCATCTAAATACCAAAAACAGACCCAACTCCCTGTCACTT

GAACTCCTTTATTTCTCCCTCTCTCTTTCTCTCTCCAAAATTTGAAAACCACCATTAATG

ATGGTGTGCTGCTGAATATTGAAAGCCATCCAACTGATTAATTGCAACTGGTAAGTTCAC

TGTACTATCCTTTATTAAAGTTTTCATCTTTAATCTCTTCATTGCACTAACACAAAACTG

TTCGAAAATGGGAAAAAGCAGAGCAAAGCAGCGTAGCAGAGCAAGAAGGAGGAAAAGCAG

AGCAAAGCAGCGTAACAGAGAAATGGTATTTGGGGTACAAAGAAGCAGATATAAGTAAGG

GAAATTAGGAGGGAAAAATTTGTCAAAGTAGGGCAAAAAAAGAGGGAAAAATTAGAATAG

GAGTGCCATTTAGAAATTGAGGCAAGTCAATGCCTGAAAAGATTGGTCAACGTCGGAAAC

CGGTGGTAAGGTCCTTCGTCACAATATTTTTTGTTGTTTGGCCTTTAAATGCCAACTTTT

TTTTTTAAGGTCCCTTCTCGCAAAAACAAAAAATTCTAAGGTCCCTTTTGACAAATTATC

C

>Mutator|12.1

GCCAAAATTGTCAACAACTACCTAATAAATATCACTTTTCATAAACAACTACCTAATATA

AAACTTTTTTAATTCAACTACCTAATATAGCTATTCCGTCAACATAACACTACCAAAACC

GTTAACTTTTGAATTTTCCGTCAACTTTTCGAAAATCCCCAATTACATGAACACTAATTT

CAATCATTTCAAGAAACCCCATTTTGATGAACCTTAATTTCATCAATTTTTCCACAAATT

CCAATTTATAAACCCAGATTTCATTAATTCCCCAAAACCCCAATTTTTAAACCCTTATTA

AATCAAGGGATCATAAACAAGCAAAGCACAAAGCCCATTATGAAGTTGAATGTATCTGTA

CAATCTGCTAGCATTCGGTGACTTTTTCACTACATTATCCGATGAGAATGTCACTGCTCC

TCCTCCTACTGTCATTCTTCTTCTATCAAATTAATCAAAAACCCTAAACACAAAATCTTC

CACACTTAAATTGTGGATTATAGTTGCAAGAGCTTAGGATCAAACACAAATGTGGGGTTG

GTTTCTTGTGTTCTTCCCTTCGCTCCACAAAATGGATGTGACGTTTATAGGGAGATGAAT

TCTTGAAGGGAGATGAAGAGCAACCCAGAAGGGAGATGAACAAATTCTTAGCCTATGAGT

CTTAGTCTCAGTCTAAGGGTTTCTGGGGTTTGGGGGAATCGAACAAATTTTAAGTAAACC

AAACAGACCATCATGATTCAATCCAAAATCCATCCAAAATCCAATGTAAAAATCACCAAA

TTAACAACAATCACCAGCACCTGCACAACCAAAATACCCATTAAGATGCTGCCAAGCCAT

ATGGTAAAAAGCCCACCTAAAAAAATGCTCAAAACCCCCAAAAATCTGGGATTACTTTGA

AAGATTCAGTTAAACTTTCACTGCATGGAGGAAGCTCAAATATGTATTTTCATCCATGAA

TTTTGGTGGAGCATTTCTACTTAAGGAAAATTAATGAAATCCCCAATTACTTAACAAATA

AGGGTTTAGAAATTGGGGTTTTTGGGAAATTAATGAAATATGGGTTTATAAATTGGGATT

TGGGGGAAAATTGATGAAATTAAGATTTATTAAAATGGGGTTTGAAATGGTTAAAGTTAG

GGTTCATGTAATTGGGGATTTTCGAAAAGTTAACGGAAAATTCAAAAGTTAACGGTTTTG

GTAGTGTTATGTTGACGGAATAGTTGTATTAGGTAGTTGAATTAAAAAAGTTTTATATTA

GGTAGTTGTTTATGAAAAGTGATATATATTAGGTAGTTGTTGACAATTTTAGC

>Mutator|13.1

GGGAAATTTCCTCAGGTACCCCTGAGGTTTGGCGTAATTCCCAAATTACCCCCCACATTT

GGGGAATTCCTCAGATACCCCTGAGGTTTGTGTTTGTGCTCAAAATACCCTTACTATGGA

TGGAAACCTAACGCCGTTAGGTTTTCCTCTCCTTCTCCCCAATTTTCTTTTTCTCTCTCC

ATCCATCATCCTCCATTGATGAACATGGAAGCTTTCTCTCTCCTCCCACCCCACTCTAAA

GCCCTAACCACCCAACCCCCACCACCTCCCCTTCCCCCCCACCCCCTGCGTCGCGCTCAG

TCGCAGCCCCTGCCTCGGCCGCCGCTCGCCGGAGCTTCGCCGGCCACCAGCATCCTCGTA

CTCTCTCTCCTTCGTCGCACTCTCTCTCTCTCTCCTCTCCTTCGTCGATTTGGACGGGTT

GGGGTGGGGGGCTGCGGGGGAAGACCACGAGTGAGGCGAGAGAGAGTTGCGAGCTCCGGC

GAACGGCAAGCGAGCAGCAGCGGTGGTCTCCGACGGTGTGGCGCGCGCGGGTCGCCGGTG

GTGCTGTACGTACAGCAGCTCGACGAAGGAGAGGAGCGAGAGAGAGAGTGCGACGAAGGA

GAGAGAGTACGAGGATGCTGGTGGCCGGCGAAGCTCCGGCGAGCGGCGGCCGAGGCAGGG

GCTGCGAGAAAATTGGGGAAAGTTTGGAGGATGGTGGGGGATGGTGGGAGGAGAGAGAAA

GCTTGGAGATTCATCCATGGAGGAGGGTGGGAGGAAGGAGAGAAACTGAAGAAAGAGAGA

AAAAAAAGTTAACGGCGTTAGGGTTCCGTCAAGAGTAAGGGTATTTTAGACCCAAACACA

AACCTCGGGGGTATCTGAGGAATTCCCCAAATGTGAGGGGTAATTTGGGAATTACGTCAA

ACCTCAGGGGTACCTGAGGAAATTTCCC

>Mutator|14.1

GGGTTTTTTGTCAGAAAGGACCTAATAAAATACAAGTTTTGTGGGGAAGGACCTAATAAA

ATTTTTTTTGTCAAAAAGGACCTAATAAAATACTTTCGATTGTGAACAGGAACCACTTTA

TGAATTTCCGGCCAAATTTACCATTTACCGGCGTTGACCCTTTTTTTTACCCTTAGTTGA

CTCTTTAATGTTCTGTTTCTATTACCGTAAAATTAGGAGGAGAAGAGAAATTACCGTAAA

ATTAAGAACCCTAATTTGCGTTTTTGGGAAAAACGTATAATAGGAGCCCTAAATTTCAAA

AACCCTTAAACATTGAGAGAGAAAAATTTGAAGAGCAAAACATAGCTTGGATTTGATGAA

GGTTGCAGGATAAGATCGAATAGGTTGATGCTTCCCTTCAATGGAGATTGCGCTGTTGGG

AGGAAAGTGTGAGAATTCAATATGTTTGGGTCAGTTAAAATAGGAACAAAACATTAAAGA

GTCAACTAGGGGTAAAAAAAAGGGTCAACGCCGGTAAATGGTAAATTTGGCCGGAAATTC

ATAAAGTGGTTCCTGTTCACAATCGGAAGTATTTTATTAGGTCCTTTTTGACAAAAAAAA

TTTTATTAGGTCCTTCCCCACAAAACTTGTATTTTATTAGGTCCTTTCTGACAAAAAACC

C

>Mutator|15.1

GAGTTAAGGTTACAACTGGGTACTAAACTTTGCCAAAAGTTCCAGTTTGGATACTCAACT

TTAAAATGTTTCAGTTTGGGTACTCAAACCAAACTTCTGTTACTTTTTCTGTTAACTCCA

ATTAAATAATAAAAAAAATATTTCTGCACAATTTTTTTTATTTCTCTTCTTTAAAAAATT

TAATATCTCTTTCTCTCTCCTCCCCATAAACACCAAATCCACTCCCACCAATCCACCACT

CCACCTCCTCCCACCACAGACCACGCCAAATTTTTTTTGCCATCGTTGCTGATACCCAAT

TATCCACCATCATCGACCCATCAAAATACCCATAAACACCATCAATTTTAAAAGAATCAT

CACCATTACTGTTCTAATCACCATCAAAACACTCATGACTTTTCCCTAAATCAAAAACAC

TAGCACACTTCACATCATCATGATCATTTTTTTTTGCTAATTAAGATAGGTAATGGATCA

ATTATACCTTCCCTATTGATGCAACGACTCTGCTCAGGAACCAAGTTCTCATTCGATTTA

ATCTGCTCTAACCAATTTGAGAAATTCTCCCCTCGCCATAGCTTGATTCTGCAGGGGTTG

GATCGATGCACGTGGTGTGAGAATCTTCTTGAATCACCACTGGAACCTCCTCCTCGATTT

CCAGCATAGAAACCCCTAAAATCGCATCCATTGATTTTAAGTTCTTCGCCTGAGAATTCG

CATCCACTGTACCTTGCTTGTTTACTGCCGATGTACTCGCCTGAAGAACTGTCGTAAGAT

CGATCTACGCAGAGAGGAATTCTAGACAGAGAAAGGCGCGAGGTTCAGTCATGTCTACAT

CATCATCTTCATCTGATGAAGAGGAGAGATAATTAATGGATTCAACCAGATTGACACTTT

GAATTACGTAAATGCTTTCATCATCAATTTCGAAATCAAATCGTGATCCCAATTGCATAT

ATCTTTTTGGGTAGAGATTGATACCATTTATTGTTTCGCCATTGAAGAATTTGAAGTTTT

TCTTGTGTTTCGTTGATTTTTCTCTGTATTTTGTTTTGTTTCAGTGGAAGTTTCTCTCTC

CAATTTTTGGGGATTTATCTCTCAAATGTTGATTTTGCTTGTGAAATGCAGCATGATGAT

GTTGTTTTGGTTGCCGGAGAGGAGCGATGGAAGCAGGAGCGGAGGAGAGAGAAAAACTAT

GGGGGAAGGGGGAGGTGGTGGAGTGGTGGTCTGGTGGATTTGATTTTGAGGGGAGGAGAG

AGAAAGACAATAATTCTTTTTAGGAAGAGAAATAAAAAAATTTGTCCAAAAACACTTTTT

TTTATTATTTAATTGGACTTAACAGCAAAAGTAACAGAAGTTTGGTTTGAGTACCCAAAC

TGGAACTTTTTAAAGTTGAGTACCCAAACTGGAACAAAGGGCAAAGTTTAGTACCCAGTT

GTAACCTTAACTC

>Mutator|16.1

GGGATATTCCATTTGGTACCATTGAATTTTTGGCTTTTCCATTTGGTACCACTGTTATTT

TAAAATTCCACATGATACCACTCTAATTTAATATTAATCTTTATCATACCATTTTTTAAG

TAAAATGTTAGTTAATGGTTGAACAAGAATACGATTTTGTTTGGGTTTGCATGTTTCTAT

TTGCAGTTGCAAAAATTTAATAAATTAAGATTTTAGGGTGTCGTTTTAACCCTTAAAGCT

CTATTTTTCAGTCAAAATTTTCATAAAAATGATCATAATATTCTATGTCTTTTTATTGTC

AAATATGACCTCAACTTTGACCTTAACGACTAAAACGATTACTAAAACCTTAACGCAAAT

TAAAACATGTAAATCGAAGTAAAACTCTAGCCTATTCTAACTTAAATTAACACTTTACTT

AGTTGAATGCTACAATAGAGAATAAAATCAAATTATAGTGGTACCATGTAGAATATCTGA

AAAATAGTGGTACCAAATGGAAAAGCCAAAAGTACAATGGTACCAAATGGAATATCCC

>Mutator|17.1

GGGTTTTTCTACGTTATGCCCTCGTGTTTTTTCATTTTCTACGTTGTACCCCATCTTTCT

GGAATTTCTACACTGTGCCCTTGTGTTATCTATTTTTAATACAAAATGCCATTGCCGTTA

AGTGGCTGTTATGTGTCCCGTTAGTGAAATTAATAATGATTAATTAATTGAATTAATTTG

AGTAGCTGTTAAAAAAACATGAGAGGGAAACTTTCCCACCAAAAAACTAGGGTTTGACTC

CCTTTTATTAACTTCTTTCCCCTTCCCCAAATCTTAATCTTCTTCCTTTTCTCTTTGAAG

CTCCTCTCCATCATCAACAATGTCAACTGCAAATTCTTACTCAGTTGCGGCCTTGGAATG

GGAATTCGTCACTCCCAAGTGTAATTGCAGCCCCCCAAAAAACGCGAGATTGCACATCTC

TGGATCTCAACGCAATCATGGTAGGGCATATTTCAAGTGCGATTTTTGCAATTTTTTTGT

GTGGTTGCACAGTGGACATATGATTAGATGAGGAGATGCTGCAGATGTTTTTTTGCTCTG

GGATTTTGTTTTTGGGACAGAGTGCGAAGAAGAGGGAGTAAGGAAGTTCAATGATCAAAA

CATGAGAGAGAGAGGAGGGGTATTTTATTTTCTTTTTTTTTTGTTCGAAAAAATTATAAA

ATTGACGGTAGGTGACACATTTAATGTCAAACCTAACGTCCGTCATTAATAAGCCCTCAT

TAATGGCATTTTGTATTAAAAATAGATAACACAAGGGCACAGTGTAGAAATTCCAGAAAG

ATGGGGTACAACGTAGAAAATGAAAAAACACGAGGGCATAACGTAGAAAAACCC

>Mutator|18.1

GGGTTTTTTGTCCCAAACTACCTTTAACTTTTATGTTTTTGCGAGAAACTACCTTATAAA

AAAATTGTTGTCCCAAACTACCTTACTTTTACCATTTTGTTGTTGACAACTACCTTAGGC

CGATTTCCGACGTTGACTTTTGTTTTTTCCGCTTTTTTATTGTCACATTATATCACTTGA

CCTCTTCAAACCCTTTTTTTAATTTTCCCCACCCTAATTTCCTCATTTTCCGGTGTGCGC

GCGCTCTCTCCCTCATATCTATGTGACACTTTGTACCACCAATCCACCATTAAATTCCTC

TTCTACTTAAACTAAAATTGAAACAATCACCCTCCTCAATTCCCACAAGGGTAATTTTGT

TTTTCCCACAATTTTGAACTGAAAAAACAAAAGTCAACGCCGGAAACCGGCATAAGGTAG

CTGTCAACAACAAAATTGTAAAGTAAGGTAGTTTAGGACAACAATTTTTTTATAAGGTAG

TTTCTCGCAAAAACTTGAAAGTTAAAGGTAGTTTGGGACAAAAAATCC

>Mutator|19.1

GGAAAATTTGCACAAAACCACCTTAAGTTTTCACTTATTTAGAAACTCCCACCTTTATGT

TTTTTTTTCCTAAATTTCCACCTAAACTTTACAAAACCTTTATAAACTCCCACCACTTAA

CGGGTTTTGCCAAACTCCATTAGTAGTGGGACCCACATCGGTTATTTCTTTTGTTTTTCC

CTCCAAACAACTCACTTCCCTCATTCATCTCTCCAAAAATGATGTCGTCTTCCTCTTCCC

ACCATTAAGTTCTCCCTTTTTTTTCCATTCTCTCTCTCTTCATTCATCTCTCTTGAAATC

TGGGTAAATTTCTGGGTTCCAGACAACATTTTTGTAGAGATGAATGAGGGAAGTGAGTTG

CTTGGAGGGAAAAACAAAGAAATAACTGATGTGGGTCCCACCACTAACGGAGTTTGGCAA

AATCCGTTAAGTGGTGGGAGTTTATAAAGGTTTTGTAAAGTTTAGGTGGAAATTTAGGGA

AAAAAAACATAAAGGTGGGAGTTTCTAAATAAGTGAAAACTTAAGGTGGTTTTGTGCAAA

TTTTCC

>Mutator|20.1

GGAAAATGATATACATAGCCCTTAGGGCTACATATAAGAAGCTAAAAATGGAAATAAAAA

GTTGAATCAAGCATTTATTACCTCAAATGATGTGTAATTTTGTCTTGAATTTTTAAACTG

TTTCTATTTTAAGAAATATATTTAATGCTTATCCATAGCCCTAAGGGCTACATATAACAT

TACCC

>Mutator|21.1

GGAAAAATTGACATGAATAATCCCAACTTTCACCAGTCTTCTCAAAATAATCCCAACTTT

GAATTATTTTAGAATAATCCCAACTTTAGGGGGTACCTTCTCAAAATAATCCGAACTATT

AATTTTCAATATATTATAGACCTAAAATGGATTAGTGTAGGCAAGAAAAGTGATAGTTTG

GTTTATTTTGAGAAGGTCCCCTCAAAAGTTGGGATTATTTTGGAATAATCCAAAGTTGGG

ATTATTTTGAGAAGACAGGTGAAAGTTGGGATTATTCATGTGAATTTTTCC

>Mutator|22.1

GAATTTTTTCCTTGAATAATGCAAAAGTGGGTTTTAATTCCAGAAATATGCCCAAAAAAA

AAATCCCTGCGTACCGTCCACGTAGGACGGCACGCGAGAATTTTTTTTTCTACTTAAGTA

AAACCGCTACTACGAATAGCGGTTTACCCTTATAAGCCGCTACTCAAAGTAGCGGTTATT

GTAAAAATAAACCGCTATTTGAAGTAGCGGTTAATATTAATTGACCGCGACTTCAAGTCG

CGGTTTACTGGTCCCTGAACCCCCCAGTCCATAGCTTGGCGCTGAGTGATATAAACCGCT

ACTTCAAGTAGCGGTTCATTAATGAAAACCGCTACTCCAAGTAGCGGTTTACTAAAATTC

CCAACTCCCCCCTAGGTTTGCCAATTATGGGGAAGCAGCACTGATCGGGGTTTTTTTGTA

CATAAACCGCTATTCGTAATAGCGGTTTACATATGCCAGAGTTTGATATGTGCTTGCAAC

ATTTTGCCCAGGTAAACCGCTACTCAGGGTAGCGGTTTATATATATAAACCGCTACTTAA

AGTAGCGGTTTATTTTAAAATAAACCGCTACTGTGAGTAGCGGTTTACGTGGGCAAAAAC

AAAAAAAAAACAGTTTCTCGCGTGCCGTCCTACGTGGACGGTACGCAGGGAATTTTTTTT

TTTTGGGCATATTTCTGGAATTAAAACCCACTTTTGCATTATTCAAGGAAAAAATTC

>Mutator|23.1

GAGGAGTGATAAAACGCTAGCAGCTAGCGTTTTAATTTGTCAATAGGAGGACTCAAAAGC

AAATAGAGGAGTGGGAAGGAATTTGGCAGAGAAAAAAAAAGCTAGCATCCATGTGTCAAC

ATGTAATTAGCACACATGTCATAATATTGTTTATCCAAAACAAATTACTTTTTGCAATTT

TTAAACTTGTATATCTCAAGAAATATATTTTAGAAAAAAATAAAAAATATACTAATATTC

ATATATTTTTATTCTCTACAAATAGACATCCATTTTGATATAATTTCATCATATATCAGT

TTTAAAATTTATATCAGTTTTAATTACCTTACGTCAAACAAGTTTATTGTTATAAGAAAC

AAGTGTATTTTAACAAGCAACAAGTGTATTGTATGGGGAAACAAGTGTATGTTAACAAGA

AACAAGTGTATGTTAACGAGAAACAGGTGTATTGTAATAATAAGAAACAAGTGTGTTATA

AAAAACAATTGTATTTCAACAAGAAACAAGTGTATTGTATTAAGAAACAAGTGTATGGTA

ATAAGAAACAAGTGTATGTTAACATAAAACAGGTGCATTGTAATAACAAGATACAAGTGT

GTTATAAGGATAAGAGTAAAATAAATATTGTATGAAATTATATCAAAATGGATGTCTATT

TGTAGAGAATAAAAATATATAAATATTGGTATATTTTTTATTTTTTTCTAAAATATATTT

CTTGAGATATACAAGTTTAAAAATTGCAAAAAGTAATTTGTTTTGGATAAACAATATTGT

GACATGTGTGCTAATTACATGTTGACACATGGATGCTAGCTTTTTTTTTCTCTGCCAAAT

TCCTTCCCACTCCTCTATTTGCTTTTGAGTCCTCCTATTGACAAATTAAAACGCTAGCTG

CTAGCGTTTTATCACAGCTC

>Mutator|24.1

GAATTTTTTCCTTGGATAATGCAAAAGTGGGTTTTAATTCCCAAAATATGTCCAAAAAAA

TTAAAATTCCCTGCGTACCGTCCACGTAGGACGGCACGCGAGATTTTTTTTTTTAGTAAA

ACCGCTACTTGCAGTAGCGGTTTACAAGTGCCGAGAATTTTTTTTTTTTCAACTTAGGTA

AAACCGCTACTCGGAGTAGCGGTTTATTATGAAAACTGATATCTGCTTACCATATTTTGC

GCAGGTAAACCGCTACTCAGAGTAGCGGTTTATATTTATAACCGCTACTCAGAGTAGCGG

TTTATTTTAAAATTAAACCGCTACTTTGAGTAGCGGTTTCCGTGGGTTAAAACAAAAAAA

AAACAATTTTCTCGCGTGCCGTCCTACGTGGACGGTACGCAGGGAATTTTAATTTTTTTG

GGCATATTTTGGGAATTAAAACCCACTTTTGCATTATTCAAGGAAAAAATTC

>uc|10.1

AGGAAAAATTGACAATAGAGGTCTGAACTTTGGGTGATAATTTTTAAAAGGTCTGAACTA

TTGATTAACCTGTGTAGGTCCCAACTTTAGGGGGTCTTAACTTTTAGCGGGCCTTAGTGA

CTTAAAACTGGCTTAATAGGTCATCACTTGTAAAACTTGGGTCACATCAGAAGGGTTAAT

TGAGGGAGGGATGTAGGAACTAGGTGAGATTTTAAAAAAAATCTTCAGTTTTAAGTCGTC

GAGGCCCGCTAAAAGTTAAGACCCCCTAAAGTTGGGACCTACACAGGTTAATCAATAGTT

CAGACCTTTTAAAAATTATCACCCAAAGTTCAGACCTCTATTGTCAATTTTTCCT

>uc|11.1

AGCCAAAATTGTCAACAACTACCTAATAAATATCACTTTTCATAAACAACTACCTAATAT

AAAACTTTTTTAATTCAACTACCTAATATAGCTATTCCGTCAACATAACACTACCAAAAC

CGTTAACTTTTGAATTTTCCGTCAATTTTTCGAAAATCCCCAATTATATGAACACTAATT

TCGATCATTTCAAGAAACCCCATTTTGATGAACCTTAATTTCATCAATTTTTCCACAAAT

TCCAATTTATAAATTAATTCCCCAAAACCCCAATTTATAAACCCAGATTTCATTAATTTC

TACTTAAGGAAAATTAATGAAATCCCCAATTACTTAACAAATAAGGGTTTAGAAATTGGG

GGTTTTGGGAAATTAATGAAATATGGGTTTATAAATTGGGATTTGGGGGAAAATTGATGA

AATTAAGATTTATTAAAATGGGGTTTGAAATAGTTAAAGTTAGGGTTCATGTAATTGGGG

ATTTTCGAAAAGTTAACGGAAAATTCAAAAGTTAACGGTTTTGGTAGTGTTATGTTGACG

GAATAGCTGTATTAGGTAGTTGAATTAAAAAAGTTTTATATTAGGTAGTTGTTTATGAAA

AGTGATATATATTAGGTAGTTGTTGACAATTTTAGCT

>uc|12.1

AGGAAAAATTGTTAGAAACTACCTATTAAAGACACTTTTTTAAAAAAAACTACCTTATAT

CATGTTTATTTTACAAAACTACCTTATAAGTGGAAAAACTTTAAAAAGCACTACCAAATT

CAGAATTTACTAACCATGGTTAACTATTTCCTCCAATTATCACTCTAATATAAGATTACT

AAACTACCCCAACCCCTCTTTGCTAATTTCCCTCACAGGAGCGTTTAACTATTGAATACC

CCCTTAAATCGTTTGTGTAATTTCTTCTCCCAACCTTTTGCTTCACCAATTAATTCTTCA

TTATGAGTAACAAAAGCAGATAGAAATTGGGTTTTGGGTCATGAATTGATGTCGTTGATG

ATGATGAAGAATATGGTTTGATGATGGTGCAAAGCTTGCTCTACTAGACACTTTTACTGC

TGGTTTGTTTTTGGGATTGACTTTTGGGGGAGAGAAGGTGTGCTTGTGCTTGCCACATGT

ATATTTGAGAGATAGAGCATCAAGTGATCAAGAAAGTGATGATATCGTAATTGTAACGTA

CTGAATTTAGGGGGTATTTTCATAATCTTATATTAGAGGGATAATTGGAGGGAGTAGTTA

ACCATAGTTAGAAAATTATGAATTTGGTAGTGCTTTTTAAAGTTTTTCCACTTATAAGGT

AGTTTTGTAAAATAAACATGATATAAGGTAGTTTTTTTAAAAAAAGTGTTTTTAATAGGT

AGTTTCTAACAATTTTTCCT

>uc|13.1

AGGAAAAATTGTCAAGAATAAGTCAACCTTTGCGTTGACCGCTTTTAACAAGTCAACCTT

CATTTATTCCAGTAGCAAGTAAAGCTTGTACATGAATTAGCTTTTAACAAGTTTTTTTAC

TTATGACCTGAACAACCCCTACAATTTTGTTCAGTTTTAACAGGTGTGTATTCTAGAACT

CATTAAATTGTTCTCTAGAACATATTTGCACTGCTTGAATTTTATAAAAGTTCCGTGAGA

AACTTAAAACTGAACAAAATTGTAGGGGTTGTTCAGGTCATAAGTAAAAAAACTCGTTAA

AAGCTAATTCATGTACAAGCTTTACTTGCTACTGGAATAAATGAAGGTTGACTTGTCAAA

AGCGGTCAACGCAAAGGTTGACTTATTCTTGACAATTTTTCCT

>uc|14.1

AGGAAAATTTGCACAAAACCACCTTAAGTTTTCACTTATTTAGAAACTCCCACCTTTATG

TTTTTTTTTCCTAAATTTCCACCTAAACTTTACAAAACCTTTATAAACTCCCACCACTTA

ACGGATTTTGCCAAACTCCGTTAGTGGTGGGACCCACATCAGTTATTTCTTTTGTTTTTC

CCTCCAAACAACTCACTTCCCCCTTCTTATATAAGAGAATTTCCCTTTTCACCCTTTGAT

CCTTCACCCACATCTTATCAGAAGGAGGTCTGCCCCCTTTGTTGCTTGTGTTGGAGGGAA

ATTCTCTTATATAAGAAGGGGGAAGTGAGTTGTTTGGAGGGAAAAACAAAAGAAATAACT

GATGTGGGTCCCACCACTAACGGAGTTTGGCAAAATCCGTTAAGTGGTGGGAGTTTATAA

AGGTTTTGTAAAGTTTAGGTGGAAATTTAGGAAAAAAAAACATAAAGGTGGGAGTTTCTA

AATAAGTGAAAACTTAAGGTGGTTTTGTGCAAATTTTCCT

>uc|16.1

TCAGGGGCGGGGCAAACATAACGGGGGCCCTATACACTCTTTATAATTGGGCCTTTTGTA

ATTTATAAAAGATATATGATTCTCAATTTAAAAAAAGTTACTTTGATATTGAAAAATGTC

TAATTACACCATATTAAAATTATAAATACATCTACTTAAAAAGTTTTTTCCATGTCCAAA

AGGATATTTTTTCAGTATATCAAGAAATTGTAAACAAACAGATATTTAGATATAAAAAAA

AGGAGTGATTGAATAAAAAGACAGAGTTGAGAGAAGTGATTAAATATTTATATCATATGA

ACCATAGAAAGTCAAAAGAATATAGGATCATTATGATAATTAATTATTAATATAGAAAAT

ACTTTTTCTTGGAAAGATAAGTAGAAAGAGAAAGTTAAGAGGAGAGAGAGTTGTGAGGAG

AAATTACCATTAATGTAGTTTGGGGAATATGAAAATTGACCGACTTAAATCAACAAAAGA

AAAAAGTAAGTAAAATTTACCTAATAAATACGCTGTGGCCCGCGTGATAGTTATGTCGGA

AACATTCAAAAAAATTAAAACACTAAATTTTGGTTGTTGTCAAGAATTGAACCTGCGTTC

CACTTTCCCTTCCCTTACAACTTTACCACTGCTCCAAACAATTTACTTTTGTCTTTCTAT

CAAACATTTGATATAAACATATATTTTGGGGCCCTTGTATTTTGGGGGCCCTATTCCGTC

GCCTATACTGCATGGCCCAAGAACCGCCCCTGA

>uc|18.1

AATAGGGAAATTCTCAGTGGTAGCCTTCAATTTTGGCTTTTTATCATTGGTAGCCAAAAA

AAAGTTTGATTATCAGAAATAGCCTTGAGGTTTTGGCTATACTTGAAAATGTGGTATTAA

CAACCATTTTTTGCCATAAACAATTGTTAAGAAGGTTGTTTTCATCGTAATTAAGGTCTC

TAGTGTCTAAAATAACATAAATTGTCCTTGACAAACTTTCTTTTGTTCATATCTTTTGTA

AAATCGAGTGAAATAAGTTTTTAATAAGACACGAAGGCAAAATAGATTTTATAAAGGAAA

CTCATATCATTCTAGCTTATGGAACCATACTTAGTTCAAAAATAACCCTTTCAACAATTG

TTTTTAACGAAAAATCGTTGTTAATGCGACATTTTCAAGTATAGCCAAAACCTCAAGGCT

ATTTCTGATAATCAAACTTTTTTTTGGCTACCAATGATAAAAAGCCAAAGTTGAAGGCTA

CCACTGAGAATTTCCCAT

>uc|19.1

CACCCGGGTGTACCATGAGCATGGTACACCCAGGGGTATTTTTGTAATAAATTAGAACTT

CAGGTGCTCATTTGACACCAAATTTGCTCATATGGGCATTTTGCTCATTTACTCATTTGC

TCATTTTGTAATAAAAGTGTTTGCTCATTTTCTTAAATGAGTAGAAATGTTTGCTCATTT

GTTCATTTTGCTCATTTGCTCATTCGCTCATTTTGCTCATTTGGAGAAAATAGTCTTGGG

TGTACCATGAGCATGGTACACCCAGGTGT

>uc|20.1

TATGCAGCCCTAAGGGCTGTATATAAGAAGCTAAAAATGGAAATAAGTTGCATTAATTGA

TCATTTATTACATCGGTTGATGCAAAATTTTTCCATAAATTCACAACTTATTTCCATTTT

AAGTAGTGTATTTATGTCTTATATGCAACCCTAAGGGCTGCATAT

>uc|21.1

CGGGGCGTTTGGTATGAGGGATTGGGAGTTTGGGGATTAGAGGATGGGATTGGGAATGGG

AGTTAGAAGGTGGGAATTCATTAAATATATCCCAATCCCTTGTTTGGTATGTTAGTGGGA

TTAAGTAGGGAATATATTGCTAAATTACAAATTTACCCATTTTAATGAAGGATAGCAAAA

GAAGAGCAAGCAATATTAGTGAAGTATATTTTAATGAAGGGTAAATTTGTCTTTTAACAT

AACCAAAAATAATCCCCCTCCCTAATACCTGCCCCCCCCCATGTCTTATTTTGGTGGGAT

AAGAGGGGTTTTAATCCCATTCCCCTTCACATGGGAATTTAGATATCCCCTCAAACAAAC

ATGGGATATCTACCCATAGGGATATAATATCCCATTCCCACCCCCTAATCCCCCTAAACA

AACACCCCGT

>uc|22.1

TGATAGAGTATAAATCTATTCCTGGGGCCTCAACCATCAGCTTAAGCTTTTGGTTGAGGC

CCCAGGAATAGATTTATACTCTATCATAC

>uc|23.1

CCCGGTGGGTAGTTAACCCACTAGAAATAGAGCATTAAAAAACTATTAGAACTGATGAGT

TGGATTCCACTATTTATTTATTTCTATTAGTAGTGGGTTGTTGACCCACTGGGT

>uc|24.1

CAATTACCTGAACTAGGGGTGATAATGAGCCTGACAAGCTCGCGAGCAGCTCGGGCTCGG

CTCGGTCAAAGCTCGGGCTCGGCTCGAGCTCGGGCTCGGCTCGAGACTTAAACGAGCCGA

GCCGAGCTAGGCAAAGCTCGGCTCATAAAGCTCGTGAACAGCTCGATTATTATTTTATAT

TAAAAAAATATTTATATTAAAAAAATAATTTATATTTTAATTTAGTTACAAATTATATAT

TTTAATTTGTGATCCAACTTCACCTCTACTCCACTAGGCACTAAAGTCCCACATTGGTTA

AGAAAACAAGGATCAACCTTATTTTCTTTATAAATAGCTACTACTCCTTACTTCAACTTA

AAGTTTAATTTTGTATCTCCTTGGTTGGAAAACAAAAACTATACTTTATTTTCTTCATAG

AGGGAATGAAGTATATTTTTAAATATTTGATTATTTAATACTTAAAATATTTGATAATTT

TTTCTTTAGCAAAACATGGAGTCCCACATTGAAATTTTAACAATGCTAGGAGGGATTCCA

AGTTATAAAAGCATATGTAAACAAAACAAGGGTATAATGAGTTTTTTTTCTTAACAAAAC

ATGGAGTCCCACATTGAAATTTTAGCAATGCTAAGAGAGATTCCAAGTTATATAAGCATA

TGTAGAATGAGTTTGTTTATCTTAAACAAAACATGGAGTTCCACATTGAAATGGGGATAG

TGAGTTTGTTTTTCTTTAACAAACCATGAAGTCCCACATTGAAATCTTAGCAATGCTAAG

AGGGAAATCAAGTTATAAAAGCATATGATGGGTGAGTGAACAAGCTCACCTACTTCGAGC

CAAGCCGAGCTCACGAGCCGAGCCTAAACGAGCTCACGAGCCGAGCTCGAGCCCCAAATT

TTAATCACGAGCTGAAGTCGAGCTCGGCTCTCCCAAGCTCGGCGAGCTCACGAGCCGAGC

CTATGAAAATTTAAACGAGCTCGAGCCGAGCCCAATACTATTCGGGCTCGGCTCGCTCGA

TAAGCACCCCTAA

>uc|25.1

AGGAATGATAAACCTCTTTTTGCTAGATGTTTGCAAACATCTAGCAGGGGTAAAACTGTC

AATATATATAGGAAATAGTTGTCAGTCAAACAAAAAAGTTATCTCGAAAATTGCAAAGTG

TCAGTCAAACCAAAAAGTAGTCGCGAAGATTCCGATGCTAAAAAATAGTGTTCATGCTAC

TAAAACTAGTGTTCGTGTAACTAAAAATAGTGTTCATGCAACTAACAAATAGTGTTCATG

AAATATAAAATAGTGTTTTATTCATCTTCTATTTCATGGGACCCATATCACCAAAAAGTT

ATCTCGAAAATTGCAGAGTGTCAGTCAAACCAAAAAGTAGTCGCGAAAATTCCGATGCTA

AAAAATAGTGTTCATGCTACTAAAACTAGTGTTCGTGTAACTAAAAATAGTGTTCATGCA

ACTAACAAATAGTGTTCATGAAATATAAATAGTGTTTTATTCCTCTTTATCTGCTAGATG

TTTGCAAACATCTAGCAAAAAGAGGTTTATCATTCCTCAT

>uc|26.1

CTACATGGTAGCCCCCAACTTTTAGGTTTTCTACATGGTAGCACTTCTTAAATTTTTTTA

TACTTGGTAGCCCCGAACTTTTAGGTTTTCTACATGGTAGCACTTCTTAAGTGAATTCCG

TTAAGTTGTCGTTAGTTTTGACATTTATTAGTCTGGCACGCAAATCGAGACTTTTTCGAC

ACCCAAAACGAGTATTTCTGATCATTTTTTGACAATTTTGACCTTTAATCGAGCCTTTAA

GGACAAAATTGCCCCCTTTCATTGTCAAAAAATGATCAGAAATACTCGTTTTGGGTGTCG

AAAAAGTCTCGATTTGCGTGCCAGACTAATAAATGTCAAAACTAACGACAACTTAACGGA

ATTCACTTAAGAAGTGCTACCATGTAGAAAACCTAAAAGTTCGGGGCTACCAAGTATAAA

AAAATTTAAGAAGTGCTACCATGTAGAAAACCTAAAAGTTGGGGGCTACCATGTAGAA

>uc|27.1

CTCTAGAGTTGTAAAATAACTCTACAAGGTAGAGTTTGAGCAATATCAACCATTGAATAA

AAAATCAATGGCTCATATATTCTCTTTCCAAAATTCCCCCTATTTTATCTCATCAAAATC

CACCCCTAAATCTTTCCATTCCACCAATATAAGCCCTTGATTTTAAAATGTATGGCTCAG

ATACAACTCTACCTTGTAGAGTTTCACTAAAACTCTAGAGG

>uc|28.1

GGTGTACCATGTGCATGGTACACCCAAGGCTATTTTTTGCTCATTTGCTCATTTGAAACT

CTACTTATTATGTGATTATGCTCATTTGGAGGTTTCAAATGAGCAATGAGCAAAAAAATA

GCCTTGGGTGTACCATGCTCATGGTACACCC

>uc|29.1

TTGACCAAATTGGTAAAAAAAAGTGACCCTCAACGACAATTTGGTAAAAAAAAATTGCTC

TAACGACAAATTGGTAAAAAAATTGAGCTCCGGCGACATTTAGGCCAATTTTTTCTATAA

TTTTCCGATTTGATCAAAACGTGCACTTTACAAGATTTTATTTCAAATTGGGTAAATAGT

GTGTAGTGACAAGAGAAGTTAATTAGATAAAAACACGAAAAATCTATATAAAAGGCATGT

AAATGCACGTATTGAATGTTGATCAAGCTGAAAAATTTTAGAAAATATGGCCTAAATGTC

GCTAGGAATCCATTTTATTTACCAAATTGTCGTCAAAACTCATTTTATTTACTTAAATGT

CGTCTGAGCTCATTTTTTTTACCAACTTGTCGCTGGAGCTCAATTTTTTTTACCAATTTA

TCGTTAGAGCAAGTTTTTTTTACCAAATTGTCGTTGAGGGTCACTTTTTTTTACCAATTT

GGTCAA

>uc|30.1

TTAGGGAAATTTCCTCAGGTACCCCTGAGGTTTGACGTAATTCTCAAATTACCCCTCACA

TTTGGGAAATTCCCCAGATACCCCCGAACTTTGACTTTGTGCTCAAAATGCCCTTACTAT

TGACGGAACCCTAACGCCGTTAGGGTTTCTTACTCTTTTTCCTCTCCATCTTCATCTTCT

TCATCTCTTCTCCCACAATCCTCCATTAATGACACCTTAAACCTTCAATTTTCTCCCTCT

CCTTCCACCTACATGCCTTTATCTCTCCTCCCATCTTCATCTTCTTCATCTCTCTTCATG

CTCATGTCTCCCATCAATCCTTTTTCAATCCCTCTTAAACCCCTCTTCTCTATCTTCTTC

ATCTCTCTCTCTCCTAAACTTCACATCCCCTTCAACTTCATTCCTCGTTGTTCTCAGAAT

AACATTCAAGCACAAACCCAGAAAACCCAACCCCCCAACATCGAGAAGAAGAAAATAAAA

CCTAGGCCAATTTTCTATGACTACATCCATGAAAATGGTGTATAAAACCCATTTCTAAAA

CCCAGAAATTCTCATGGCAGCTTCAATAATAAGAACAACAACAATAACCATCATTAATTT

GGGGAAAATTAACAAGGAGAGAGAATTAACTTCACCATCATAAAAAATGGGGGAAATTCA

CAAGATGAAGATGGGAAGGAGAGATAGAGGCATGTAGGTGGAAGAAGAGGGAGAAAATTG

AAAGTTTAAGGTGTCATTAATGGAGGATTGTGGGAGAAGAGATGAAGAAGATGAAGATGG

AGAGGAAAAAGAGTAAGAAACCCTAACGGCGTTAGGGTTCCGTCAATAGTAAGGGCATTT

TGAGCACAAAGTCAAAGTTCGGGGGTATCTGGGGAATTTCCCAAATGTGAGGGGTAATTT

GAGAATTACGTCAAACCTCAGGGGTACCTGAGGAAATTTCCC

>uc|31.1

GTGTTTGGCAATTAGAGTTTTGGAGCAAAAAGAGAGGTTTGGACCACTTTTAGAGTTTTG

ACTAGTCAAAAACTCTAAAAGGGGTGTTTGGTAAAAGAGAGTTTTAGAGAGAGTTTTGGA

ATGAAAACTCTCAAAATGAAAAAGCTCCTATTAGGAGCTTTTTCTTTAGAGGATTAGGTA

GAAATATGTTTAATGACTTTTTTGCCCCTAATAATAATACTACACATATGTATTAAAATG

TCTATAAATGTCATTTTACACAAACAAATGGCAATAAGCTTTTAGTAATCAGTTAATTTT

TACCAAACACATTTATAAAAAAAGTTGTCAAATCAGCAGTCAAATCTGCTGAACAAAAAG

TTAAAACTCTAACAGCAAACTCTAAACTCTAACAGCTAAACTCTAAACTCTATTTGCCAA

ACAGGGCC

>uc|32.1

ATATACTTCCTCCGTTTCTTTTTACTTGCAACGGTTTGACTTTTACACTATTCACCAACT

CTACTTAGTTTATGTTTGGTGATTTATAGTTAAGGAAAAACATATTCATGTGGGATCTTG

TTAGATTCGTCTGAATGTATATTTTCCGAATATATACTTTTTATAATTTTTATTTATCGG

TAATTAAACTTATTGATGGTTGAAATTATGCATTGGCAAGCGTGAAAGTCAAACTGTTGC

AAGTAAAAAGAAACAGAGGAAGTATAT

>uc|33.1

GTTCAGTTCAGTTCAGTTCAGTTCAGTTCAGTTCAGCTCTTGACAATACTTTTACTCTCA

CATATCACTATTCATTTTCATTCAGTTCAGTTCAGTTCAGCTGTTTTATGCCGAAGAGAA

CAGGCCCTT

>uc|35.1

TTTCTAACCTATGCACCTAGTGCAAAGGTTAAGCCTTATCTATTAAACAAATTAATTAAC

TAAAATAGAATATCTTCCCTATAATTATGCTAAATAGTTAGTTTCCTTATTAATATATAT

TTTATCTTTTCTTTTAATCAAATCAAAATTGTCATTAATATATATTTAGCATAATTATAG

GGAGGATATTATATTTTAGTTAATTAATTTATTTAATAGATAAGGCTTAACCTTTGCACT

AGGTGCATAGGTTAGAAAAA

>uc|36.1

CTTAAGTACCAAAGTACTTAAGTACGTACTTAAGTACTTTGGTACTTAAGT

>uc|37.1

TGTAACACCCCTCTGGGGACGAGCCCAGAGAGGACAATATCTACTAGCGGGCTCGTGGTC

CCCAGAGGGGTGTT

>uc|38.1

TTAGGGAAATTTCCTCAGGTACCCCTGAGGTTTGACGTAATTCCCAAATTACCCCCCACA

TTTGGGGAATTCCTCAGATACCCCTGAGGTTTGTGTTTGGGCTCAAGATACCCTTACTAT

GGACGGAACCCTAACGCCGTTAGGTTTTCTCCCTCTCTCTCCCCAATTTTCTCTCTCCTC

CCATTCCCCCACCCACTGCCAACACCCCTCCCCCAGCCGGCGACCAACAGCCGGCGACCA

CCACCCACTTCGGCGCCGGCGACCCTACCCCCTCCCTTCGCCGGCCAGAACAGCAGCACC

CCCGGCGACCCTACCCCTTCCCTTCGCCGGCCAGAACAGCAACACCCCTGGCGACCCTAC

CCTCTCCCTTTCCCCCACCCTCGACACCCCACCCTAGCCCTCCCTTCGCCGGCCAGAACA

ACAGCACCCCGACACCACCCCCTCCCCCCTTTCCCCCTATCGGAAATGTCCCCCCTTCCC

TGTCTCCCACCGTGAGTCGACCCCCTTCCCTTCCCCACCCAGATACGCCGCCCACAAACC

CTTAAACCCGCCCTCAAAACCACCCTAGGCTGTCGTTCTGCTGTTCTGGCCGGCGCCAAC

GCCGAGGGTGAGGGAATCGGGAATGGGGAAGGGAGGGGTAGGGTCGCCGGCGCCGGAGTA

GGGGAGGGGGGTTTGGCAGTGGGTGGGAGAATGGGAGGAGAGAGAAAATTGGGGAGAGAG

AGAGGGAAAACCTAACGGCGTTAGGGTTCCGTCCATAGTAAGGGTATCTTGAGCCCAAAC

ACAAACCTCAGGGGTATCTGAGGAATTCCCCAAATGTGGGGGGTAATTTGGGAATTACGC

CAAACCTCAGGGGTACCTGAGGAAATTTCCC

>uc|41.1

ATGTTTGGATAGCAAGATTTCATTTCAAATCCTGGATTTGGATGAAATCTATTGTTTGGA

TGGCATGGAATTTGAAATTATGGAATTTGTCCAAATTCTTATCAAAAGCCTATATTAGTA

GAATTTCAAATTCAAGCTATTACCTTGTCATTTGAAATTCTCTCCTCATACATATCCCAA

CTCCATGAGTAAACCCACACTTTCTAATATTTATTTCTCTCTCCTTTAGTAAACCCACAC

TTTTTCTCTCTCCTCCATTAAACCTCTATTTCTCTCTCCTCTATCAAACCCCCTTTACAA

AACAGTGATTAGGAATTGAAATACAAACTTTAAATTCCAGATTTCAATTCCATTATTTGA

AATGAAATACAAGTTTCCAAACAC

>uc|42.1

CCCACTGGGTAGACTACCCATTAGAAATAGACAATTAAAAAATAAATAATTCTGCTGAGT

TGGACTCCACTTATTATTAATGTCTATCTCTAGTGGGTAGATTACCCAGTGGGTA

>Stowaway|1.2

CTTCCTCCGTTTTTTAATAAGTGACACGTTTTCCTTTTAGGGAAATGTCACAATAAGTGA

CACATAGCTCAAAGTAGTAAAAAATACTTCTCATTTATTACATCTTTGCCATCATTTAAT

TGTTTCACCTACCTACTTTATCCAATACTCAAATTTGATTGGTTAAACAAGTGGTCCCAA

CTTTCTCTCTCTAGGCTCCTTGGTATTCCCCCACTTTCTCTCTGTGTCACTTATTAAAAA

ACGGAGGAAG

>Stowaway|8.2

CTTCCTCCGTTCTAAAATAAGTGAAACACTTCTCTCAAAAAGCTCTCACATAAAAGAGAG

AAGTGTTTCACTTATTTTAGAACGGAGGAAG

>Stowaway|15.2

CTTCCTCCGTTTCGTTTTAAATGCAACAAATGAATATTTTATATCTCACAAATAATACCC

CTTTGTTGCATTTAAAACGAAACGGAGGAAG

>Stowaway|17.2

ACACCCCGACCCATACGGAGTCGAGTATATTGTCACCAATGCGCATCCAGGGCCAACAAC

TGTCCTCAATAACCAACACAAGTTCTTCCGGCGCATTTTGTCCTCACTCATGCGCACCCT

GAGAAACTTCCCAGGAGGTCACCCATCCCTGGACTACTCCCAGCCAAGCACGCTTAACTG

TGGAGTAACTAAGGAAATAAGCTCCCTAAAAGAAAGATGCATCTTGTTGGAATGAGTAGC

CTGCTAAATCCCTTTAAAGCAATCATGAGGGGTAT

>Stowaway|20.2

CTTCCTCCGTTTCATAATAGATGCCACATTGTACATTTTTGGGTGTTTCAAAATAGATGC

AACAATGTTTTTTTTTCCTTATTTAGTAACTTTTGCCTTATACTTTTACCATTTTACCCT

TACTAACTTGTTTTAAGTACACTAATCTACTTCACTTTATATGTGGTCCCCAACAGTTTT

TTTTTCCCTCCTTTCACATGGGGACCACTACAATTAATCTACTTCACTTTATACTACTTA

AACATGCTTAGGAAATTTTGCCTAGGAGTCCGTTTTAACAACAATGTGGCATCTATTATG

AAACGGAGGAAG

>Stowaway|26.2

CTACCTCTGTTTTTTTTAGTTGCAACGATTAGACATAAAGTGTGAGTAGAAAATGTCTAA

TCGTTGCAACTAAAAAAAACAGAGGTAG

>Stowaway|28.2

CTCCCTCCGTCCTTTATTAGTTTACCCCTTTTGACTTTTCACATTTATCGATGTAAAACT

TTGAGTAATAATATCTCTAATTATGTATTTGTAAAAATTATAAAAATTTAATATTCCAAA

ACTACATATTGAGACGAATCAAACAATATCACACATGATTATATTTTCCTTACATATAAA

GTACAAATGACGGTCAAAGTAATAAAGATGAATAGTGTTATAAGTCAAAGTGGGTAAACT

AATAAAGGACGGAGGTAG

>uc|1.2

GGGGAAAAAAAGGAAGGGCTGCGCAGGCTTTTCCTAAAAGCGCAGCCCTACTAGTGAAGG

GCTGCGCTTTTTTTAAAAGCGCAGCCCTACATAGCAGAAGGGCTGCGCTTTTGAAGAAGC

GCACCCTTCACTTAGTAGGGCTGCGCTTTTAAAAGCGCACCCTTCTGTTATGTAGGGCTG

CGCTTTTGAAGAAAAGCGCAGCCCTTCTTAAAAAAATTTGAAATAAAAAATTCGCGGGCT

TCAAATATTAAGCGGGCTTCGTCAAATATATTATCCCTCATTCTCTCTCCTCTCTCACGT

CCCTCCTCCCTTTTCCTCCTTCTCTCACGTCCCTCCTCCCTTTCCTCCTTCTCTCTCATC

CTCTCTTCTCTCTCAAAAACTCCCAGAAACGTTGCGACAAAAAGCCTGAAATGATTTGCA

CTAATCATGCCGACGGAGTCCGATTGCCTATTGTGATACCTTTTCGGAAGCGGAACGGGC

CAACGAGTCAAACTCACCGCTATGCCGTGACGTTTAGGCTTTTTAAGCCATCCGAGTTCG

TTTAAGGCTCCAAATAGGCATTTTAAGTTTTTCCGATTTTTTTTTTCCTTAATTATGTAT

TAGGGTTTTGTTTTATGTATTTAAACATTATTAGGTTAATGTTTGGAGGTAGTTCTTGTA

TTTTCAGATCTATAATTTCTTAATCTTTGTTTTGATTAATGTTCTTGTATTTTCAGATCT

ATAATTTCTTCTCTTCTGTATATTAGTTTTTGTTCCTTTTCGTTTCGTTCTTTTTTTTTG

TTTTGGGTTTAGTTGCATTGTAGATCTATAATTTCTTCTCTTCTGTATATTTGGGTTTAG

TTGCATTGTAGATCTATAATTTCTTCTCTTCTGGATTTGGATGGTTTACTTTAAAGTTTA

ATGCAAATGTTAAAATTCAGAAAATGTGAGATTAATAGTAAAAAAATGTCGAAAATTTTT

TTTTTTTTTTTGGTAGGAAGGGCTGCGCTTTTTAGGTAAAAGCGCAGCCCTTCTAGCTTG

AAGGGTTACTAAAAGCGCAGCCCTTCCTTGTGAAGGGCTGCGCTTGTCTAGCGCAGCCCT

ACTTAAAAAGTGAAGGGCTGCTATTGAAAGGGCTGCTGTCAAAAAGCGCAGCCCTACAGG

CCTTTTTCAGCGCAGCCCTTCTTCATTTTTCCAC

>uc|2.2

GAGTATAAATCTATTCCTGGGGCCTCAACCATCAGCTTAAGCTGATGGTTGAGGCCCCAG

GAATAGATTTATACTC

>Tourist|4.2

GAGCAACTTCAATGGTTAGCTAAAACAATACTTGGCTAAAGATGCCACATCATTTTCCTT

AGCTAATTCTATCTTTAGCCAACTTCCCTTTAGTGGTTTAGCTAAACTATTTTGTTGCTT

AGGTAGGTCCCACAATCACTTTTTCCTTTTTTTTAATATATAAAATATCACTTATTGAGA

AGCTATTTGTTGGGTGGGACCCAACATTTTGGCTAAGCCAATTTGCTCTATAGAGCCAAT

TTGCTTAGCAAAGCAAAACTCCATTTTGTGCTCCAATGGTTAAGCAACTAGCTAAGTATT

TTGCAAAATTGCTAGCAAGTCCTTTTTTTGCACCATTGGAGTTGCTC

>Tourist|10.2

CAGTTTGTTTGGTAGAAGGAATTTGGAAGGAAAGGAAAGGAAGTGGAAGGAAATTTATTA

CTTTCCAATGTTTGGTTCATTAAAAAAAAATTAAGTGAAAGGAAATGGGAGGAATTGGAA

GGAAAGACTCTTTAATAAAATTTCACTTTCCTTTCCTCCCAAAAGCGGGGGAATTCGGAA

GGAAAGAGAAAATTTATACCAAACATGGGAAATTAAGGTTTTTTTCCTTTCTCTTCCATT

CTTTTCCTTTCTCTTCCCTTCCTTTCTCTTCCCTTTCTTTCCTTTACTAAAAATGAACCA

AACACACTG

>Mutator|1.2

GCTAAAATTGTCAACAACTACCTAATATATATCACTTTTCATAAACAACTACCTAATATA

AAACTTTTTTAATTCAACTACCTAATACAGCTATTCCGTCAACATAACACTACCAAAACC

GTTAACTTTTGAATTTTCCGTTAACTTTTCGAAAATCCCCAATTACATGAACCCTAACTT

TAACCATTTCAAACCCCATTTTAATAAATCTTAATTTCATCAATTTTCCCCCAAATCCCA

ATTTATAAACCCATATTTCATTAATTTCCCAAAAACCCCAATTTCTAAACCCTTATTTGT

TAAGTAATTGGGGATTTCATTAATTTTCCTTAAGTAGAAATTAATGAAATCTGGGTTTAT

AAATTGGGGTTTTGGGGAATTAATGAAATTGGGTTTATAAATTGGAATTTGTGGAAAAAT

TGATGAAATTAAGGTTCATCAAAATGGGGTTTCTTGAAATGATTGAAATTAGTGTTCATG

TAATTGGGGATTTTCGAAAAGTTGACGGAAAATTCAAAAGTTAACGGTTTTGGTAGTGTT

ATGTTGACGGAATAGCTATATTAGGTAGTTGAATTAAAAAAGTTTTATATTAGGTAGTTG

TTTATGAAAAGTGATATTTATTAGGTAGTTGTTGACAATTTTGGC

>Mutator|2.2

GGAAAAATTGTCAAGAATAAGTCAACCTTTGCGTTGACCGCTTTTGACAAGTCAACCTTC

ATTTATTCCAGTAGCAAGTAAAGCTTGTACATGAATTAGCTTTTAACGAGTTTTTTTACT

TATGACCTGAACAACCCCTACAATTTTGTTCAGTTTTAAGTTTCTCACGGAACTTTTATA

AAATTCAAGCAGTGCAAATATGTTCTAGAGAACAATTTAATGAGTTCTAGAATACACACC

TGTTAAAACTGAACAAAATTGTAGGGGTTGTTCAGGTCATAAGTAAAAAAACTTGTTAAA

AGCTAATTCATGTACAAGCTTTACTTGCTACTGGAATAAATGAAGGTTGACTTGTTAAAA

GCGGTCAACGCAAAGGTTGACTTATTCTTGACAATTTTTCC

>Mutator|4.2

GGAAAAATTGTTTTAAATAAGTCAACCTTTGCGCGGTCTTCCCATTTTAAGTCTACCTTT

TGATTATCCCTGATTGAGTTAAGCTTGTATGTCTGTTTTCTTCTATTGAGTTTTAACCTG

TTTGTGACTTGTTTAGTAGGTTACTTATGATACGTGTTACAATGTCCCACCCCCACCCTC

GCCTCCCTCGCCTGTTTTAGAAGAAGGGACATTGTAACACGTATCATAAGTAACCTACTA

AACAAGTCACAAACAGGTTAAAACTCAATAGAAGAAAACAGACATACAAGCCTAACTCAA

TCAGGGATAATCAAAAGGTAGACTTAAAATGGGAAGACCGCGCAAAGGTTGACTTATTTA

AAACAATTTTTCC

>Mutator|7.2

GGGTAATGCTAAATGCAGCCCTTAGGGCTGCATATAAGCATTAAATACCTTTCCTAAACT

AGTATAAAGTTGGTTATTTATGACAAAAAGCATGCATTTATCAAGGTCATTAATGCTTGT

TTTACAAAATAATTTCCATTTATAGGTTCTTATATGCAGCCCTAAGGGCTGCATTTAGCT

TTTCCC

>Mutator|9.2

GGGTTAATTGGCTAGAACAGCACAACCTTGGGCTCAAGTGCAAGGAACAGCACAACCTTA

AACGGGTGCAAAGCATAGCACCAACTTAAACACGTGGTGCAGAAAATAGCATAAATGGCA

CCGGAGCACGGAGATTCCACCGGAGCTAGGGAATCTCCATTATTTTAAGGGTTAATTGGC

AAGGAACAGCACAACCTTTGGCTCAAGTGCAAGGAACAACACAACCCTTATTTTAATAAA

CACACCATTTCTTAACCTCCACTACACTAAATCTACCTTAAACCACACTACATCACCGCC

ACCCTCTTCCCTAGCCCACAACACTACAACACCACCACCTCCCTCAACACCATCATCTTC

CTTCACCACACCAAACACCACCTCTACTCCAACAATGTCATCCTCTGCCCCAAACACTGC

AGCACCACCACACCAAACACCATCTCCACCACAACCTCGCATTACTCCCAGTCCACAAGA

GCAGGCGCTCCGTACTAATTCACCACAAAACAGAAGAGTTCCAGAAAGTTTGAGCGACCA

TGGCGCGACCTCGAAGCAGGCGCTCATCAAACCAACTCTTTTACAAACAGCACGTGGAGT

GGTCTTTGGTGAATCAACAGTGAATATTGAGCTAGGAGAAACTGAGATCAGCCTCCACAA

TTGTTACACGTCAATTATTAATGATGATAGATGTAAATCCAGCGACTTGATTTCGATATT

TGCGCTGATGCAATTTGTTGATGATGTGAAGATCTGTGTTGCGTCGAACAGGGAAGAAAA

TCGCAAAATGAGGAAGTAGCACAGCAGCGAAGAAGAGTAAAGAGGAAATCAATTTTTTTT

TTGAGATTGAAGTTGAAAGTTGGCTAGAACAGCACAACCTTTGGATCAAGTGCAAGGAAC

AACATAACCTTGAACGGAGATTCCCTAGCTCCGGTGGAATCTCCGTGCTTCGGTGCCATT

TATGCTATTTTCTGCACCACGTATTTAAGTTGGTGCTATGCTTTGCACCCGCTTAAGGTT

GTGTTGTTCCTTGCACTTGAGCCAAAGGTTGTGCTGTTCTAGCCAATTAACCC

>Mutator|10.2

GGAAAAATTGTCAAGAATAAGTCAACCTTTAAGCCATCCGCTAAAAACAAGTCAACCTTT

TGATTATCCTTGAATAAGTCAACCTTTAATACCAATTTGTTTTTAACAAGTCTTTGCCTG

TTGTTAACTTATTTAACCGCTTAAATATGATATGTGTAACTCTTCAAAGGGATGAGAGAG

AAAACAAAAAGAAATTAAAAATTACAAAAAAGAAAGAAAAAAGTCATATTATTTGTAATT

TAACTTGTTTAAAATTGATAAGTACTAAAGGTAGACTTATTCAGGAAATAATCAAAAGAT

GACTTATTTTTAGCGGACGGGACAAAGGTTGACTTATTATTGACAATTATTCC

>Mutator|11.2

GGAAAAATTCACAAGAGAGAGCTCAACTATTACCCATTTTCTTTTATAAGTCCCAACTAT

GAATTATTCCGTACAGGTCCCAAGTTTACTATCTTGTGATTTTCATAGGTCCTGAGTTAC

TTATAACCGGTTTAAAAGGTAAGATTTTTAAATGTTTACCATCTTCGAACGCTTTCAGGA

AGTGACAAAGACCTATAAAATGGGGCCAACATTTAAAAATATTACCTTTTAAACCGTTTA

TAAGTAATTCAGGACTTATAAAAGTCACAATATGGTAAACTTGGGACCTTCAAAGAATAA

TCCATAGTTGGGACCTATAAAAGAAAATGGGTAATAGTTGGGCCCTCTCTTGTGAATTTC

TCC

>Mutator|13.2

GAGAAATGATAAACATCTATCTGATAGATGTTTGCAAACATCTATCAGGGATAAATCTGT

CAATGTATATGGGAACAAGTTGTCAGTCAAACAAAAAAGTGATCTCGAAAATTGCAAACT

GTCAGTCAAACAAAAAAGTGATCTCGAAAATTGCGAGCTGAAAAATAGTGTTCATGCTAC

TACAACTAGTGTTCGTGTAACTACAAATAGTGTTTATGCAACTAACAAATAGTGTTCATG

AAATATAAATAGTGTTTTATTCCTCTTCTATTTCTTGGGACCCACATCACCACCTTTTCA

GCCTTTTATTATTTCCTGGGACCCACATCACCACCTTTTCAGCCTTTTATTATTTTTTAT

TCCCTGATAGATGTTTGCAAACATCTATCAGATAGATGTTTATCATTTCTC

>Mutator|15.2

GAGAAATGATAAACCTCTAACTGCTAGATGTTTGCCAAACATCTAACATTCATGTGGCAA

CATATAAGTGGTACACATGTCATTATATTGTTTATCCAAACAAATTACTTTTTCCAATTT

TTTAAACTTGTATATCTCAAAAAATATATTTTAGAAAAAAATAAAAAATATACCAATATT

CATATATTTTTATTCTCTACAAATAGACATCCATCTTGATATAATTTCATCATATTTTTA

TTTTACTCTTCTCTATCTCTTATTTAGATATGTAAAAAAAAAATACGAACACTATCTGTT

TATACACAAACACTATTGTGAGTTGCATAAACACTAGTTATAGTTACACGAACACTAGTT

CTAGTAGCATGAACACTGTATATTAGCATCGGAATCTTCTGACAGTGTCCAATTAAAAAA

ATACGAACACTATCTGTTTATACATAAACACTATTTTTAGTTGCATAAACACTAGTTATA

GTTACACGAACACAAGTTGTAGTAGCATGAACATCATATATTAGCATCGAAATTTTCTGA

CAGTGCCCAATTTTCGAGATTACTTTTTTGTTTGACTGATAACTTTTTTTCATGTATATT

GACAATTTTACCCCTGCTAGATGTTTATAAACATCTAGCAGTTAGAGGTTTATCATTTCT

C

>uc|4.2

CTCTAGAGTTTCAAAATAACTCTACACGGTAGAGTTCGTTAAATATCAGTCGTTGATTAA

GAAATCAAAGGCTTGTATTTTCTTTCCCAAAAATCCCCCCTATTTTCCCCCGAATATCCT

GCCCATATCTTTTCCTTTCCCACTAATCATGAGCCCTTGATTTTATAATATATGGTCAGG

ATTCAACTCTACTTTGTAGAGTTTTCATAAAACTCTAGAGG

>uc|5.2

TATATAAGCCAAGGGAGGAGGTGTGGTGTCCTCTTTTGAAGTGACTAAAATACCCTTAAT

GATCTAATTAATGGAATGAAAAAAATTACAAAAGCAAATCATTTAAAAATTCTCATTCCA

ACTTGCCATTTAAAACAAGTTTAAAATGGTATATGCATCAAATACTTCAATCAAAAATTA

AATGGACAAAATATTTATACCTTATAAAAAAAGATGGCATACATAACGTGCACATGAAAA

GTCTGAACATTCACTACCTTTGATACTCTTTAATTCAGTTAAACCAACTCATTATTTTTG

TTCTTTCTGTTCAATATTCATTCACAAAAAATTAATGTTAACAATCACTCACAACCTTTT

TATTTTTTTACATTAGCGAATTAATATATCTAAATTCATCTAACTGTTGAATTTTTCACA

TTTAAGTTTAATCATAAATAAAGTACGAATGGAGTATCTAATAGAATAACAAATTTTGAA

ATTAACGCACGCATCGCGTGCATATGAAGGCTAGTATTC

>uc|7.2

CCTGGACTCGGCTAGGAGTGTCGAAAATGGGTACGGATCCGAGTGTCCGGTACGGCACTT

CCAAAAAATCAGGACACGGGGACTGTCCGGAATTTGGACACGGGTACGGGTATGCGAAAT

TATTATTTTACAATATTAATTTTAATAATTAATATTACTTATAAAAATTAATATTACCGT

ATGGTAGTATAAAAATATAAACAACAAAGATAATTATTGAACAAATATAGAGAAAAAGAT

AAGGGAAGAGCAAAGGCTACTTTGGTCTTTATTAATTAGGTATGTATAATATTTGAGAAA

AAATAACTAATATTGCAAAAGAATCTTTAGATAGTATATCACGTGACATCTTATAAAAGG

AGAAAGATGATAAAGCCCCTTGGCCCACCTATTTTTTTAATTAAAAAAGTAAAATATGTG

AGTTGAAAGAAACCCACGTTTTCTCTCACCAAGTTGGCCCACCAAGTATGTTCATCTTCT

TCCTCTTCAACTGTTTCTTCTTCTCCTTCAACCTCTGTCCTTTGCAACTCCATTCCCTCC

TACCATCCATCAAAAATGGATGAAAACAACATCAATGGAGTCGCCTCACTAGGGACGCAG

AAAGCCCTAACATAATTCACCATATTCTCATGAAGCAACATCAAAATTTCAGATCTAGCA

ACTAGGGTTCAGCGCCGTGTCCGACTGCAATTTGAAGGATCCGACACGTATTTCATACCC

GAGTCGGTGTCGTGTCGTACCGACCCGGGTATTTGGCGAAAAATGCCGAGTCGAGGT

>uc|9.2

AGGTGCGCACATGTACATGGCTAACAAGCCAAATGTACATGAATAACAAGTCAAATGAAC

ACATGTACATGGCTAACAAGCCAAATGAACATATGTACATCTCTGTTAGTATATGTTCAT

TGGTATTGGTCTTTATGTTCATAACCTAGTGCTTGATGTTCATCGTGCGCACCTTCCTCA

CCATACGGACGTGT

>uc|11.2

CCAGGGTTACCTAATTCGCCCCTACCCCCCACGAATCATGTTTCGATTCGTCCGTTCCGA

AACGCAATTCGTTCGCGCACCAAATCGGTAGGGAACGCGACTTAGATCGTTAAAATTGGC

GTTCCAAATGGTGGGTAGAGATTTCGAGATTTAGGGTTTGAATAAACCCTAATTTTGTTA

ATTGAAGGGGAAACATAACAAAATTTGTGCTTTAATTATGGGGCTTTTGATAGGAATTTA

AAATCGAGTTGATTGAAGTGTTTAGGGTTACCTATTCTCCTTCGGTTGTCTTCTCCGGTT

GTCTTCTCCTCATCTGGTGGCTGCGGTGGTTGTGGGCGCCTTGAGGTGTTTTTGTTTTTT

TTTTTTACGGGTAGGCAAGAGGTTGAAGATGAATGTTCATCTTCATGAACTTTTATTAGG

CAATGCAATGAGTGTTTTGGTTGATAGAGACTTGGTGGTTCACTGGCTCGTTGATATTAG

AGAAAGTGGAAATGAATGAGTGAGGAAAATGAAGGGTGAGGATTAGTCTAGAGAATGTTA

CTATATACTCACCATTCATTTTCCTCACCATTCACTGTATTAAGATTTTTTTTTAGGGAA

TTGGGGTACATGGGAAAAATAAGAACAATAACTTACTATTTCAAGTTTCAACCTTTCTTC

CTTTCCCCATAATTAAAGAGCCACGTTTTGGGTTAATTATCATTTGTTTATTGTGGCATT

GTAATTGATTCTCAAACTTCTTTTTTTTTAACGACTGAATATTATTAAAAAAATTATAGT

CAAATGAGTTCCTTTTTTTTATAATTATTAGTGCGTATTATGTTCCATCGAATTATGTTC

CTAAAATCCGTTCCGAAAAAAAATCCCATCACGATCCAAATTCTTTCGACTAGCGAATCG

CGTTCCCGTTCCCGTTCCGCGATCCAAGACGATCCGCGAATTAGGTAACCCTGTTTTG

>uc|12.2

AATTGTTGGAATAAAAGGTTCGCAAAGGCAGAAATTAAGCTTTCAAAAACTGTGCCGTGA

TAGGAGATCACTGACTTGAAAGCAATTACGGCCCGACAATGGTGGTTATCGATCTTGGCC

GGAATGCTCCCCAAGGTTTACACAGCAGCTAAGAATGCCACCACTAGCAAACTGAGCTTT

AGAGAATAGCACGAAAACCCAGGAAGAGAGAAGAGAAGAGAAGAGAGAAGAGTGTTTGTT

TTTGTGTGTTTCTGACTGAACAGAAGGCACTGATTTTATAGGGAAAAATCAGGCCAAAAA

CGGGCAACAATCAAGACAGAATTAATTGGGCGGATAATTAGGGATTGCCATGAATAATGT

AACGGTCAAAACGTTACAGAATTCCAACAGCCACACACGCATAGCTCCCGGGTCCAAAAC

TTTAGTTAAGTTAAGTTTATTCCCCAAAGCCCAAAGCCCAAGGCCCATGGCCCTGGCCCG

GTCCGGGCGGGCGGCGCGCGCGTGTGTGATAGCAAGCCCAAACCAAACATATCATCCAAC

CACAAAAGCATCTCTAGTCACAACTCAATAGATGGAAGGAGGGAGGAAGTATATAAACAT

GAAGAAATTCCCTCCCACAAGCAATGTGGGACAAACAAGAAAATGAGCCAAGGCTTTTCC

AAGCCTTAACTCCAACAAT

>uc|15.2

ACCCACCGGGTAGACTACCCGCTACTAATAGACAATTAAAATATTAGTATTTAAGTCTAG

TTGGATTCCACTATCATAATTACCACAACCTTATATTATGGGGTGGGTAGTCTACCCGGT

GGGTT
